# Postharvest Storage Practices of Maize in Rift Valley and Lower Eastern Regions of Kenya: A Cross-Sectional Study

**DOI:** 10.1155/2020/6109214

**Published:** 2020-01-31

**Authors:** Peter Koskei, Christine C. Bii, Protus Musotsi, Simon Muturi Karanja

**Affiliations:** ^1^School of Public Health, Moi University, P.O. Box 4606-30100, Eldoret, Kenya; ^2^School of Public Health, Jomo Kenyatta University of Agriculture and Technology, P.O. Box 62000-00200, Nairobi, Kenya; ^3^Kenya Medical Research Institute, P.O. Box 54840 00200, Mbagathi Road, Nairobi, Kenya

## Abstract

An assessment of local farmers' knowledge, attitude, and practices on postharvest maize storage and management was carried out with a view of understanding its role in maize contamination with mycotoxins and postharvest losses in Rift Valley and Lower Eastern Regions of Kenya among 165 and 149 farmers, respectively. Differences between the two regions were analyzed using the Chi-square test, Fisher exact test, and two-sample *t*-test. The median quantity of maize harvested by farmers in the two regions after shelling was 585 kg. A median of 20 kg of maize was put aside as a result of rotting before shelling, and there was a significant mean difference in maize set aside as a result of rotting between the two regions (107.88 kg vs. 31.96 kg; *t* (306.25) = 5.707, *P* value <0.001). The quantity of discoloured and mouldy maize consumed ranged from 0 to 90 kg; 7 (2.2%) respondents consumed mouldy maize, 36 (11.5%) fed it to cows, and 19 (6.1%) fed it to poultry. A small percentage (3.5%) believed mouldy maize is safe for human consumption, 23.6% for animal consumption, while 15.0% considered it safe for brewing, with the differences between the two regions being statistically significant (*P* value <0.05). Nearly half of the respondents (48.4%) kept maize on cobs indoors, 47.1% left it in the field without covering, and 33.1% consumed and sold maize while still green, with more farmers from Lower Eastern practicing this. The results of the study suggest that there were poor postharvest practices and low awareness levels among maize farmers and that this can lead to postharvest losses due to *Fusarium* spp. infection and mycotoxin contamination that poses a threat to human and animal food safety. This calls for interventions on better postharvest practices.

## 1. Introduction

Agriculture is the backbone of the economy in most Sub-Saharan African (SSA) countries, contributing significantly to their Gross Domestic Product (GDP) [1]. In this sector, grains are its major product [2], of which maize is the main contributor.

Maize is a vital food crop cultivated in most parts of the world, especially in low- and middle-income countries (LMICs). Globally, it is the third most grown cereal crop that serves as the primary source of food to more than one billion people [3]. It is a staple food in many LMICs, especially in SSA, with the annual per capita consumption varying from country to country [4, 5].

Most of the consumed calories in East and West Africa are from maize [6]. In Kenya, maize and its products are the staple food to the majority of the population [7], with an annual per capita consumption of approximately 77 kg [8]. Most of the maize harvested is stored in many parts of the country to ensure continuous supply between seasons. Traditional storage methods are mainly used by farmers [9] but have the disadvantage of being susceptible to insects and pest attacks [10], among other unfavourable conditions that might lead to contamination and spoilage.

More than 34 million hectares of maize were grown in SSA in the season of 2014/2015, producing approximately 70 million metric tonnes. Even though the area under maize cultivation in Africa has increased, the production and supply have declined [11]. This has been attributed partly to climate changes and heavy postharvest losses [12].

These losses have been estimated at 20–30% of the total staple maize harvested [13], valued at approximately US$4 billion annually [14]. The high postharvest losses are associated with poor maize postharvest practices, informal marketing systems, and unfavourable physical and environmental factors. Other factors include pests and fungal attacks [15, 16]. In particular, insect pests have been associated with high maize grain damage and losses. Approximately 30% of the losses in maize grains in Africa are a result of postharvest insect pests, which cause nearly 40% weight loss in maize grains [16, 17].

The postharvest losses affect food security and food safety situation in Africa. They contribute to high food prices due to scarcity and hunger [13]. The limited food supply has further escalated many conflicts, armed wars, and political instability in some parts of Africa.

With a lack of proper effective and affordable storage methods, farmers resort to crude storage methods. Maize stored using such methods experience substantial pest damage, and rodent attack [18]. Poor postharvest practices also result in mould growth, dry matter loss in the grains, and reduced grain quality [19].

Maize is also susceptible to fungal infestation, especially of the *Aspergillus* and *Fusarium* species. This is common from the period of its growth to harvest, transport, and storage, which exposes it to mycotoxin contamination associated with these fungal species, especially aflatoxins and fumonisins [20]. Maize and its products have been frequently cited to be contaminated with high aflatoxin and fumonisin levels [21, 22]. Mycotoxin contamination has been reported to be a major problem in SSA. This is enhanced by poor farming practices, climatic conditions, and postharvest handling practices, which provide a conducive environment for fungal growth and subsequent production of mycotoxins, as well as insect infestation [23]. Mycotoxin contamination is associated with huge losses to the farmers and to human health [24, 25].

Mycotoxins are described as “silent killers” as they are hard to detect, and some are extremely toxic to both humans and animals [26] because of the damage they cause to the immune system [27]. The mycotoxins that often occur in maize grain, which are of public health concern, include aflatoxins, zearalenone, deoxynivalenol, fumonisins, and ochratoxin [28, 29]. However, in SSA, the most prevalent classes of mycotoxins are aflatoxins and fumonisins [30, 31].

There is a scarcity of information on how local farmers manage their maize after harvest, with only a few studies carried out on the same. In a study in Kaiti district of the Lower Eastern part of Kenya, most farmers were observed to apply traditional practices in determining the time for maize harvesting and subsequent storage. Maize was mainly stored in polypropylene bags in granaries and living houses. This was associated with high cases of rodent attacks, mould growth, and insect damage. *Aspergillus* species and aflatoxin contamination were high in the maize of farmers who relied on such traditional postharvest practices [32].

With limited data available, this study sought to assess the farmers' knowledge, attitude, and postharvest practices with a view of understanding their role in maize contamination and making policy recommendations to reduce postharvest losses and maize contamination, especially by mycotoxins. The findings of this study will also be useful in informing the design of intervention measures that would create postharvest management awareness among farmers.

## 2. Materials and Methods

A comparative cross-sectional study was carried out in Rift Valley and Lower Eastern regions of Kenya. Three counties from each of the regions were selected and interviews conducted among maize farmers. In the Rift Valley region, the study was carried out in Bomet, Nakuru, and Trans-Nzoia counties, which are the grain baskets of the country, while in the Lower Eastern region, the research was conducted in Machakos, Makueni, and Kitui counties that are considered aflatoxin hotspots in Kenya [33].

A random sample of 314 farmers was used in the study, of which 165 respondents were from the Rift Valley region and 149 were from the Lower Eastern region. To be eligible to participate in the study, the respondent had to be a household head above 18 years of age who was a primary decision-maker on household maize storage practices.

A pretested semi-structured questionnaire, divided into three sections, was used to collect data from the respondents. The first section was on respondents' demographic information. The second section was on maize consumption practices, while the last part was on the respondents' knowledge, attitude, and practices on maize postharvest handling and storage. The questionnaires were administered with the help of trained research assistants. The collected data were entered into MS Excel, where cleaning was done. It was then imported into Statistical Package for Social Sciences (SPSS) version 24.0 for analysis. Descriptive and comparative statistics were used for the analysis. For comparative analysis between the two regions, the Chi-square test and Fisher exact test were used for categorical data, such as gender and level of education of respondents, quality of harvested maize, insect control practices, maize storage practices, and other maize postharvest practices. A two-sample *t*-test was used to compare the means of continuous variables, such as age of respondents, acres of land, quantity of maize taken from storage for consumption that was discoloured, quantity of maize after shelling, and the average maize selling price. A *P* value of less than 0.05 was considered statistically significant.

### 2.1. Ethical Considerations

Ethical approval for the study was granted by the Institutional Research and Ethics Committee (IREC) of Moi University and Moi Teaching and Referral Hospital, approval no. FAN: IREC 1829 on 2 March 2017. We obtained permission for the study from the respective county governments through the assistance of the National Commission of Science, Technology, and Innovation (NACOSTI) Research Clearance Permit No. 14093 on 12^th^ May 2017. Kiswahili language was used to explain the study details to all respondents who spoke and understood it, being a national language. Both oral and written informed consent was obtained from the respondents.

## 3. Results

### 3.1. Characteristics of the Farmers

Of the 314 respondents in the study, 165 (52.5%) were from the Rift Valley region, while 149 (47.5%) were from the Lower Eastern region. The distribution of respondents per each of the region and counties included is shown in Table 1.

The mean age of the respondents was 42 years. Farming was the main economic activity for 233 (74.2%) of the respondents. The median acreage of land owned in the previous year and cultivated land in the last 12 months was 2.0 acres and 1.4 acres, respectively. The demographic information of the respondents is presented in Table 2.

### 3.2. Quantity of Maize Harvested from the Study Sites

The median quantity of maize harvested after shelling was 6.5 90 kg bags (585 kg) per season. Most farmers harvested one 90 kg bag (90 kg) of maize, while the maximum quantity after shelling was 270 (90 kg) bags (24,300 kg) per harvesting season. There was no statistically significant mean difference in quantity of maize harvested for the Rift Valley (*M* = 17. 18, SD = 20.99) and Lower Eastern (*M* = 12.74, SD = 33.99) regions; *t* (276) = 1.333, *P* value <0.184.

The median amount of maize disposed before shelling as a result of rotting was 20 kg. The maximum amount was 900 kg. There was a statistically significant mean difference in the maize disposed before shelling due to rotting for Rift Valley region (*M* = 107.88, SD = 131.70) and Lower Eastern region (*M* = 31.96, SD = 103.45); *t* (306.25) = 5.707, *P* value <0.001.

### 3.3. Household Maize Consumption

More than half (62.4%) of the respondents' households consumed more than 30 kg of maize grains in 30 days, while 118 (37.6%) consumed less than 30 kg in the same period. Of the consumed maize, 178 (56.7%) of respondents reported that all of it was from their production, 75 (23.9%) said that half of the consumed maize was from their production, while 61 (19.4%) reported having bought all of the consumed maize. There was a statistically significant difference in the quantity of maize consumed from own production between the two regions, with many farmers from the Rift Valley consuming maize from their production as compared to those from the Lower Eastern region (*X*^2^ (2) = 55.709, *P* value <0.001) (Table 3).

The median number of days the maize stayed in storage was 90 days. The mean difference in the number of days the maize lasted in storage for Rift Valley (*M* = 140.04, SD = 76.73) and Lower Eastern (*M* = 71.24, SD = 51.69) regions was statistically significant; *t* (219.82) = 7.981, *P* value <0.001.

The quantity of maize grains taken from storage for human consumption in the month of the study ranged from 0 to 1,350 kg, with a median of 30 kg. Of this maize, only four (1.3%) of the respondents reported that it was of bad quality. The quantity of discoloured maize consumed ranged from 0 to 90 kg, with a mean of 3.6 kg. There was a significant difference in the mean of discoloured or damaged maize for Rift Valley (*M* = 4.62 kg, SD = 13.86) and Lower Eastern (*M* = 1.94 kg, SD = 5.96) regions; *t* (215.37) = 2.110, *P* value = 0.036.

Mouldy maize was reported to be disposed by 98 (31.2%) respondents, while 36 (11.5%) use it to feed the cows, 19 (6.1%) feed it to poultry, 7 (2.2%) consumed it, 2 (0.6%) sold it, and 2 (0.6%) used it for brewing.

The respondents' beliefs and perceptions regarding mouldy maize are presented in Table 4. There was a significant difference between the two regions with regard to perceptions on mouldy maize (*P* value <0.05), except on the perceptions on the consumption of wet/smelling but good-looking maize (Table 4).

### 3.4. Storage Insect Control Methods

Chemical insecticides were the most widely used method of insect control by 222 (70.7%) of the farmers, while 102 (32.5%) used sun-drying and 25 (8.0%) used ash. On chemical insecticide use, 108 (48.6%) respondents used Actellic Super, 99 (44.6%) used Actellic Dust, 6 (2.7%) used Skana Super, while 2 (0.6%) used Malathion Dust. Many farmers from the Lower Eastern region used Actellic Dust compared to those from the Rift Valley (*X*^2^ (3) = 29.622, *P* value <0.001), while Actellic Super was used by a high proportion of farmers from the Rift Valley (*X*^2^ (3) = 53.645, *P* value <0.001). The differences were statistically significant (Table 5).

### 3.5. Other Postharvest Maize Storage Practices

The mean number of days the maize was kept on cobs was 33 days, with a range of 0–150 days. The difference in mean number of days the maize was kept on cobs in Rift Valley (*M* = 37.82, SD = 37.15) and Lower Eastern (*M* = 27.30, SD = 20.23) regions was statistically significant; *t* (258.53) = 3.157, *P* value = 0.002. The median number of days of drying the maize after shelling and before storage was seven days. The mean number of days the maize was dried for Rift Valley (*M* = 7.02, SD = 6.51) and Lower Eastern (*M* = 13.00, SD = 11.42) regions was significantly different; *t* (229.82) = − 5.62, *P* value <0.001.

Less than half, 148 (47.1%), left the maize in the field without covering after harvesting, with more farmers from the Lower Eastern region leaving their maize on cobs in the field without covering than those from the Rift Valley region (88 (59.1%) vs 60 (36.4%)) (*X*^2^ (1) = 16.187, *P* value <0.001).

Only eleven respondents (3.5%) took maize to commercial storage facilities, while the rest stored it at home. The practice of consuming and selling the maize while still green was practiced by 104 (33.1%) of the respondents, with a high proportion of respondents from the Lower Eastern region practicing it (71 (47.7%) vs 33 (20.0%)) (*X*^2^ (1) = 27.025, *P* value <0.001).

All farmers interviewed used sun-drying and airing to dry their maize. One hundred eight (34.4%) respondents practiced storage of maize on dehusked cobs, 52 (26.6%) practiced the storage of maize in husks, and 307 (97.8%) stored maize after shelling. Most of the respondents, 306 (97.5%), reported that their grains were dry last season. For those who reported that their grains were not dry, they said that it was due to poor weather, poor seed quality, and short drying season. The majority, 309 (98.4%), reported that their storage facilities were cleaned before storing the new maize grains (Table 6).

Among the respondents, 120 (38.2%) knew their maize grains were dry when they were hard to chew, 95 (30.3%) by listening to the grains' sound and measuring its weight, 49 (15.6%) by touching the grains, 17 (5.4%) by observing changes in colour, and 16 (5.1%) when the maize cobs are hit during shelling. Other methods used to know when the maize grains were dry included observation during shelling as reported by 8 (2.5%) respondents, and storage in store for one week as reported by 4 (1.3%), after sun-drying as reported by 2 (0.6%) and during milling as was reported by 2 (0.6%) of the respondents.

### 3.6. Perceptions on Factors Attributable to Maize Grain Spoilage

Of the respondents, 98 (31.2%) thought that poor soil condition contributes to the spoilage of the maize grains. Higher proportions of the respondents (88.5%, 89.8%, 91.4%, and 82.2%) thought that bad weather, wetness of the piles of the harvested maize, dampness of the storage place, and harvesting maize earlier than usual, respectively, were the main reasons for the spoilage. A small proportion (16.9%) thought that drying maize longer than usual can lead to maize spoilage. There were significant differences in perceptions on poor soils, wet weather, and wetness in maize pile, dampness in the storage places, and the presence of insects and pests in the storage places between the farmers in the Rift Valley and Lower Eastern regions. The proportion of farmers who thought that these caused spoilage of maize grains was higher among Rift Valley respondents than that of the Lower Eastern region (*P* value <0.05) (Table 7).

### 3.7. Perceptions on Minimizing Mould Infestation

Up to 186 (59.2%) of the participants thought that spreading chemical insecticides over the grains prior to storage would reduce mould growth, with the proportion being significantly higher in the Rift Valley than in the Lower Eastern region (110 (66.7%) vs. 76 (51.0%)), (*X*^2^ (1) = 7.952, *P* value = 0.005). Two hundred ninety-three (93.3%) thought that the storage of completely dry maize only would reduce mould growth. There were smaller proportions of participants who believed that storage of maize in plastic bags (70 (22.3%)), plastic containers (39 (12.4%)), metallic silos (96 (30.6%)), and clay pots (107 (34.1%)) would help minimize mould growth. There was a significantly higher proportion of respondents from the Lower Eastern than Rift Valley region who thought that maize storage in plastic bags (*X*^2^ (1) = 18.367, *P* value <0.001) and containers (*X*^2^ (1) = 31.941, *P* value <0.001) would minimize mould infestation (Table 8).

### 3.8. Maize Disposal Practices

Among the farmers interviewed, 99 (31.5%) had taken maize from their storage for sale one month before the study. The median quantity of maize sold was 630 kg, with the minimum amount being 90 kg, while the maximum amount was 12,600 kg. There was no significant difference in the mean of quantity of maize sold for Rift Valley (*M* = 1229.27 kg, SD = 1568.52) and Lower Eastern (*M* = 540 kg, SD 756.35) regions; *t* (97) = 1.764, *P* value = 0.081.

The median selling price of a 90-kg bag of maize was Ksh 2,500 (US$ 25), the minimum price of a bag of maize was Ksh 1,000 (US$ 10), while the maximum price was Ksh 7,500 (US$ 75). There was a significant difference in the mean of selling prices per bag of maize for Rift Valley (*M* = Ksh 2531.22, SD 761.99) and Lower Eastern regions (*M* = Ksh 3470.59, SD = 1766.62); *t* (17.25) = -2.151, *P* value = 0.046. Business people were the largest buyers, 74.2% (69), followed by local consumers, 15 (16.1%). Other buyers were cereal dealers, 6 (6.5%), and schools 3 (3.2%). Fourteen farmers (14.7%) who sold maize had mouldy maize in their sale (Table 9).

## 4. Discussion

The low education level among the majority of the respondents contributed negatively to their postharvest practices and, by extension, their lack of awareness of the consequences of maize contamination [34]. Most of the respondents had low income, a likely indicator of the low economic status of most respondents who depended on farming.

Most farmers harvested 90 kg of maize per season. With the per capita maize consumption in Kenya having been reported to be 77 kg [8], the majority of the farmers' production in our study could not meet the requirements for the household consumption per season, using the national per capita consumption as the reference.

The majority of the farmers in this study exhaust their maize from the stores by the ninth month after harvest, with only 39.3% having maize in their stores by the next harvest season. This was also the case in a study by Thamaga-Chitja et al. [35] where the average length the maize lasted ranged from 5.6 months to 8.6 months, showing that the maize is exhausted before the next harvest. In case the maize was exhausted before the next harvest, the farmers bought maize to cater for deficits [35], as was shown in our study. This could be as a result of low maize production. It could also be due to the farmers selling off most of the harvested maize, thus leading to food insecurity.

Some respondents believed that mouldy maize is safe for human and animal consumption. The belief, as mentioned above, was also reported in the AfloSTOP survey carried out in North Rift and eastern parts of Kenya, where 25 of the 27 farmers with mouldy or discoloured maize fed it to their livestock while some mixed them with the mould-free maize for human consumption [36]. In Ghana, Akowuah et al. found that the majority of maize farmers and traders believed that there were no health effects in consuming mouldy maize due to the rigorous cooking process of maize-based foods [37].

### 4.1. Storage Insect Control Methods

Insect attack has been reported to contribute significantly to the loss of maize grains. In a study by Midega and colleagues, insect pests were reported to result in approximately 40% grain loss, with the maize weevil and grain borer being perceived to be the most dangerous [38]. The majority of the farmers (70.7%) in our study areas practiced some form of insect control with the use of chemical insecticides, while 32.5% used sun-drying and 8.0% used ash. However, the effectiveness of traditional insect control methods such as the use of ash is not known and needs to be evaluated further.

Our study found out that the use of chemical insecticides was the primary insect control method followed by sun-drying. This was different from the study by Midega et al., who found out that in Kenya, aeration/sun-drying were the leading insect mitigation practice in their study areas, as was practiced by 88.8% of the study participants [38].

While chemical insecticides were the most widely used storage insect control method in our study areas, their effectiveness has been shown to reduce with the period of storage [38]. In the study by Midega and colleagues, the addition of chemical insecticides kept the insect damage at 25% during the first four months. However, by the sixth month, the damage increased up to 80% in the chemical insecticides added maize. The damage was more in insecticides added maize stored as grains than those stored in the unshelled form [38].

### 4.2. Other Postharvest Maize Storage Practices

Postharvest maize management practices, which include drying, cleaning, and storage, among others, are essential and critical along the maize value chain. Farmers dry their maize either during storage or when the maize is still in the field. Kaaya and Kyamuhangire [39] reported that any delays in maize drying while in the field could result in losses during the period of storage.

The current study findings are related to what was found in the AfloSTOP survey, where the farmers in both Rift Valley and Eastern parts of Kenya dried maize on cobs for a median of seven days per season [36].

According to Addo and colleagues [40], the majority of farmers in SSA make use of different storage practices, including the use of wooden baskets, jute bags, polyethylene bags, thatched structures, and raised platforms. These methods of maize grain storage are associated with *Fusarium* spp. [41] and *Aspergillus* spp. infestation leading to fumonisin and aflatoxin contamination.

According to Potter and Hotchkiss [42], the storage of maize and other farm produce on the farm is widely practiced in Africa, accounting for nearly 85% of the national storage. However, such storage methodologies are ineffective and are associated with maize contamination with fumonisins, aflatoxins, insect damage, and moulding.

According to the findings of a study by Thamaga-Chitja and colleagues, most farmers left their maize in the field to dry before harvesting [35]. This was also the case in Ghana, where most farmers heaped and left the maize on the field after harvesting [37]. The process of leaving harvested maize in the field after harvest before storage provides a suitable environment for insect and fungal infestation [15]. Udoh and colleagues [43] reported this to be a widely used practice in SSA, and this has been attributed to labour challenges and the requirement to allow the maize to dry well before it is stored.

Only eleven respondents (3.5%) took maize to commercial storage facilities, with the rest storing it at home. Poor postharvest practices and storage have been reported to play a critical role in the maize contamination with aflatoxin and fumonisins. In the study by Kamala et al., storing maize without the addition of chemical insecticides and drying the maize on bare grounds were significantly associated with aflatoxin and fumonisin contamination [44].

With large quantities of maize consumed while still green, the harvests are significantly reduced. With the small farm sizes of the majority of the farmers, the harvested maize is exhausted early. Consuming or selling the maize while still green leaves the household with low yields which cannot last till the next season. This practice is rampant in Kenya, with media reports in the country highlighting its effects of reducing the final dry maize output and hence impacting negatively on food security, with stakeholders requiring the government to come up with a policy to regulate the practice [45].

As was the case in this study, a study in Tanzania found that most farmers cleaned their storage facilities and cleared it of old maize grain stock before loading them with a new stock [44]. This was also the practice by the majority of the farmers in Guatemala, where 98% of the farmers were reported to clean their maize storage facility before storing freshly harvested stock [46]. According to a study by Mwangi and colleagues, cleaning the stores and drying the maize grains before storage were associated with minimal losses [47].

Similar to the findings of our study, Kamala and colleagues reported that farmers in Tanzania tested for grain dryness by biting or listening to the sound produced by the maize grains [44]. In Ghana, the farmers were reported to check for maize dryness using their teeth by biting [37]. In Guatemala, farmers also used these and other traditional practices, where they made use of fingernail tests (32%), mouth test (16.9%), and a combination of visualization and sound (45.4%) tests to check for maize dryness before storage [46]. Such traditional practices are not accurate and might lead to maize being stored while still having high moisture content, hence making it susceptible to fumonisin and aflatoxin contamination [48].

## 5. Conclusion

Inadequate knowledge on better postharvest practices among farmers has been reported to be among the challenges that farmers have to overcome to be able to reduce losses associated with postharvest practices [49, 50]. However, training of farmers on the same practices was not associated with reduced losses since trained farmers were noted to experience a similar level of losses as those who did not have any training on the same. These results point to the fact that farmers might not necessarily apply what they learnt, and this might be attributed to the lack of the required equipment and technologies and the necessary capital [51].

A large percentage of food products are lost during postharvest practices in SSA [8]. Hence, there is a need to invest in better postharvest practices to reduce losses. Furthermore, the demonstration of cost-benefit by putting in place mitigation measures is necessary, and hence the need to provide evidence of the effects of postharvest losses and the quantities involved [52]. Reducing postharvest losses can go a long way in the achievement of food security, hence motivating the farmers' interest in postharvest losses mitigation.

Farmers interviewed had inadequate knowledge of proper postharvest practices, with some of their practices being associated with postharvest losses. This affects the quality of maize as poor postharvest practices expose the maize grains to contamination that might cause health effects on consumers. This calls for urgent interventions on better maize postharvest management practices.

## Figures and Tables

**Table 1:** Distribution of respondents by region and county.

Region	County	Frequency (*n*)	Percent
Rift Valley	Bomet	50	30.3
	Nakuru	65	39.4
	Trans-Nzoia	50	30.3
Total		**165**	**100**

Lower Eastern	Kitui	49	32.9
	Machakos	50	33.6
	Makueni	50	33.6
Total		**149**	**100**

**Table 2:** Demographic information of the respondents.

No	Demographic information	Total (*n* = 314)	Rift Valley region (*n* = 165)	Lower Eastern region (*n* = 149)
1	Gender			
	Male	130 (41.4%)	68 (41.2%)	62 (41.6%)
	Female	184 (58.6%)	97 (58.8%)	87 (58.4%)

2	Education level			
	None	34 (10.8%)	13 (7.9%)	21 (14.1%)
	Primary school	132 (42.0%)	69 (41.8%)	63 (42.3%)
	Secondary school	99 (31.5%)	51 (30.9%)	48 (32.2%)
	Tertiary	49 (15.6%)	32 (19.4%)	17 (11.4%)

3	Occupation			
	Business person	23 (7.3%)	14 (8.5%)	9 (6.0%)
	Permanent employment	43 (13.7%)	27 (16.4%)	16 (10.8%)
	Full-time farmer	241 (76.8%)	123 (74.5%)	118 (79.2%)
	Others	7 (2.2%)	1 (0.6%)	6 (4.0%)

4	Monthly income			
	Ksh 0–5,000	198 (63.0%)	87 (52.7%)	111 (74.5%)
	Ksh 5,001–10,000	64 (20.4%)	42 (25.5%)	22 (14.8%)
	Ksh 10,001–15,000	21 (6.7%)	16 (9.7%)	5 (3.3%)
	Over Ksh 15,000	31 (9.9%)	20 (12.1%)	11 (7.4%)

**Table 3:** Household maize consumption practices by region.

No		Total (*n* = 314)	Region		*X* ^2^	d*f*	*P* value
			Rift Valley (*n* = 165)	Lower Eastern (*n* = 149)			
1	*Household consumption in the last 30 days*						
	≤15 Gorogoros (30 kgs)	118 (37.6%)	56 (33.9%)	62 (41.6%)	20.727	3	<0.001
	Over 15 Gorogoros (30 kgs)	196 (62.4%)	109 (66.1%)	87 (58.4%)			

2	*Quantity of consumed maize from own production*						
	All	178 (56.7%)	125 (75.8%)	53 (35.6%)	55.709	2	<0.001
	Half	75 (23.9%)	28 (17.0%)	47 (31.5%)			
	None	61 (19.4%)	12 (7.3%)	49 (32.9%)			

**Table 4:** Participants' perceptions on mouldy maize.

No	Participants' beliefs	Total (*n* = 314)	Region		d*f*	*X* ^2^	*P* value
			Rift Valley (*n* = 165)	Lower Eastern (*n* = 149)			
1	Mouldy maize is safe for human consumption	11 (3.5%)	2 (1.2%)	9 (6.0%)	1	5.399	0.020
2	Mouldy maize is safe for animal consumption	74 (23.6%)	60 (36.4%)	14 (9.4%)	1	31.611	<0.001
3	Consuming milk from cow fed on mouldy maize is safe	87 (27.7%)	61 (37.0%)	26 (17.4%)	1	14.894	<0.001
4	It is safe to mix wet and dry maize for storage	12 (3.8%)	1 (0.6%)	11 (7.4%)	1	9.782	0.002
5	It is safe for human to consume good-looking but wet/bad smelling maize	7 (2.2%)	3 (1.8%)	4 (2.7%)	1	0.270	0.604
6	It is safe to sell mouldy maize to local brewers	47 (15.0%)	41 (24.8%)	6 (4.0%)	1	26.670	<0.001

**Table 5:** Insect control measures used in the study sites.

	Insect control measures	Total (*n* = 314)	Region		d*f*	*X* ^2^	*P* value
			Rift Valley (*n* = 165)	Lower Eastern (*n* = 149)			
1	Use of chemical insecticides	222 (70.7%)	117 (70.9%)	105 (70.5%)	1	0.007	0.932
2	Sun-drying/airing	102 (32.5%)	68 (41.2%)	34 (22.8%)	1	12.078	0.001
3	Ash	25 (8.0%)	1 (0.6%)	24 (16.1%)	1	25.674	<0.001
4	None	3 (1.0%)	2 (0.6%)	1 (0.3%			0.224^*∗*^
5	*Chemical insecticides used*						
	Actellic Dust	99 (44.6%)	33 (28.2%)	66 (62.9%)	3	29.622	<0.001
	Actellic Super	108 (48.6%)	77 (65.8%)	31 (29.5%)	3	53.645	<0.001
	Skana Super	6 (2.7%)	6 (5.1%)	0 (0.0%)			0.031^*∗*^
	Malathion Dust	2 (0.6%)	1 (0.9%)	1 (1.0%)			1.0^*∗*^

^*∗*^Results are for Fisher's exact test.

**Table 6:** Postharvest maize storage practices.

No	Postharvest maize storage practices	Total (*n* = 314)	Region		*X* ^*2*^	d*f*	*P* value
			Rift Valley (*n* = 165)	Lower Eastern (*n* = 149)			
*Postharvest maize cob management methods*							
1	Leave maize pile in the field without covering	148 (47.1%)	60 (36.4%)	88 (59.1%)	16.187	1	<0.001
2	Bring home and pile in a separate room	152 (48.4%)	102 (61.8%)	50 (33.6%)	25.039	1	<0.001
3	Leave maize pile in the field covered	106 (33.8%)	24 (14.5%)	82 (55.0%)	57.397	1	<0.001
4	Dry off the ground on tarpaulin	125 (39.8%)	28 (17.0%)	97 (65.1%)	75.697	1	<0.001
5	Consume and sell as green maize	104 (33.1%)	33 (20.0%)	71 (47.7%)	27.025	1	<0.001
6	Take to commercial storage facility	11 (3.5%)	3 (1.8%)	8 (5.4%)	2.920	1	0.087
7	Method of drying – sun-drying	314 (100.0%)	165 (100.0%)	149 (100.0%)			
8	Cleans storage facility of all previous year remnants prior to storing	309 (98.4%)	165 (100.0%)	144 (96.6%)			0.023^*∗*^
9	*Frequency of storing maize on dehusked cobs*						
	Never	206 (65.6%)	117 (70.9%)	89 (59.7%)			0.037^*∗*^
	Always	108 (34.4%)	48 (29.1%)	60 (40.3%)			
10	*Frequency of storing maize as grains*						
	Never	7 (2.2%)	7 (4.2%)	0 (0.0%)			0.011^*∗*^
	Always	307 (97.8%)	158 (95.8%)	149 (100.0%)			
11	*Frequency of storing maize in the husk*						
	Never	262 (83.4%)	134 (81.2%)	128 (85.9%)			0.264^*∗*^
	Always	52 (26.6%)	31 (18.8%)	21 (14.1%)			
12	*Agrees that own grains were dry last season*						
	Yes	306 (97.5%)	161 (97.6%)	145 (97.3%)			1.000^*∗*^
	No	7 (2.2%)	4 (2.4%)	3 (2.0%)			

^*∗*^Results are for Fischer's exact test.

**Table 7:** Factors farmers attributed to maize spoilage in the study regions.

No	Perceptions on factors attributable to maize grain spoilage	Total (*n* = 314)	Region		*X* ^*2*^	d*f*	*P* value
			Rift Valley (*n* = 165)	Lower Eastern (*n* = 149)			
1	Poor soil	98 (31.2%)	60 (36.4%)	38 (25.5%)	4.301	1	0.038
2	Dampness in storage place	287 (91.4%)	159 (96.4%)	128 (85.9%)	10.895	1	0.001
3	Wetness in piles of harvested maize	282 (89.8%)	155 (93.9%)	127 (85.2%)	6.482	1	0.011
4	Harvesting maize earlier than usual	258 (82.2%)	141 (85.5%)	117 (78.5%)	2.569	1	0.109
5	Wet weather during harvest	278 (88.5%)	153 (92.7%)	125 (83.9%)	6.020	1	0.014
6	Insects/pests in storage place	159 (50.6%)	101 (61.2%)	58 (38.9%)	15.557	1	<0.001
7	Drying maize longer than average	53 (16.9%)	29 (17.6%)	24 (16.1%)	0.120	1	0.729

**Table 8:** Practices to minimize mould infestations of maize in the study sites.

No		Total (*n* = 314)	Region		*X* ^*2*^	d*f*	*P* value
			Rift Valley (*n* = 165)	Lower Eastern (*n* = 149)			
1	Spreading insecticides over the grains prior to storage	186 (59.2%)	110 (66.7%)	76 (51.0%)	7.952	1	0.005
2	Storage of completely dry maize only	293 (93.3%)	158 (95.8%)	135 (90.6%)	3.332	1	0.068
3	Grain storage in a plastic bag	70 (22.3%)	21 (12.7%)	49 (32.9%)	18.367	1	<0.001
4	Grain storage in a plastic container	39 (12.4%)	4 (2.4%)	35 (23.5%)	31.941	1	<0.001
5	Grain storage in a metallic silo	96 (30.6%)	46 (27.9%)	50 (33.6%)	1.189	1	0.275
6	Grain storage in a clay pot	107 (34.1%)	48 (29.1%)	59 (39.6%)	4.09	2	0.100

**Table 9:** Maize disposal practices in the study sites.

No	Maize disposal practices	Total (*n* = 314)	Region		*X* ^*2*^	d*f*	*P* value
			Rift Valley (*n* = 165)	Lower Eastern (*n* = 149)			
1	Participants who took maize from storage for sale in the month of harvest	99 (31.5%)	82 (49.7%)	17 (11.5%)	53.167	1	<0.001

2	Median selling price	2500	2400	3250			

3	*Quality of grains sold* (*n* = 99)						
	Poor	3 (3.0%)	3 (3.7%)	0 (0.0%)	0.864	2	0.642
	Fair	1 (1.0%)	1 (1.2%)	0 (0.0%)			
	Good	95 (96.0%)	78 (95.1%)	17 (100.0%)			

4	*Buyer of the largest sale in the month* (*n* = 93)						
	Business people	69 (74.2%)	58 (71.8%)	15 (86.7%)	3.021	3	0.388
	Cereals dealer	6 (6.5%)	8 (7.7%)	0 (0.0%)			
	Local consumer	15 (16.1%)	13 (16.7%)	2 (13.3%)			
	School	3 (3.2%)	3 (3.8%)	0 (0.0%)			

5	Participants who had discoloured, damaged, or mouldy maize in their sale	14 (14.7%)	12 (14.6%)	2 (15.4%)			

## Data Availability

The raw SPSS data used in this study are included within the supplementary information file.

## Additional Points

*Limitations*. The study depended on the respondents' recall of some aspects; a method found to be ineffective as a result of distortion and recall bias. The study was descriptive, making it practically hard to generalize the findings even though it provides an insight into postharvest practices of farmers that might apply to many parts of the country and beyond. The survey was carried out during one harvest season. Hence, it might not represent the general practices as they might vary from one season to the next.

## Supplementary Materials

Supplementary Materials SPSS template of the raw data used in the manuscript.

## References

[1] AGRA (2017). *Africa Agriculture Status Report 2017: The Business of Smallholder Agriculture in Sub-saharan Africa*.

[2] World Bank (2011). Missing food: the case of post-harvest grain losses in sub-saharan africa.

[3] IITA (2009). *International Institute of Tropical Agriculture (IITA)—Maize (Zea mays) Crop*.

[4] Hassan R. M., Mekuria M., Mwangi W. (2001). Maize breeding research in Eastern and Southern Africa: current status and impact of past investments made by the public and private Sectors 1966–97.

[5] Pingali P. L. (2001). CIMMYT 1999/2000 world maize facts and trends. *Meeting World Maize Needs: Technological Opportunities and Priorities for the Public Sector*.

[6] Macauley H. Cereal crops: rice, maize, millet, sorghum, wheat. An action plan for African agricultural transformation.

[7] Ransom J. K., Palmer A. F. E., Zambezi B. T. Maize productivity gains through research and technology dissemination.

[8] FAO (2011). *FAO Statistical Databases (FAOSTAT)*.

[9] Nukenine E. N. (2010). Stored product protection in Africa: past, present and future. *Julius-Kühn-Archiv*.

[10] Lathiya S. B., Ahmed S. M., Pervez A., Rizvi S. W. A. (2008). Food habits of rodents in grain godowns of Karachi, Pakistan. *Journal of Stored Products Research*.

[11] De Groote H., Hall M. D., Spielman D. J. (2011). Options for pro-poor maize seed market segmentation in kenya. *African Journal of Biotechnology*.

[12] Auffhammer M. (2011). Weather dilemma for African maize. *Nature Climate Change*.

[13] Yusuf B. L., He Y. (2011). Design, development and techniques for controlling grains post-harvest losses with metal silo for small and medium scale farmers. *African Journal of Biotechnology*.

[14] FAO (2010). *Reducing Post-harvest Losses in Grain Supply Chains in Africa: Lessons Learned and Practical Guidelines*.

[15] Hell K., Mutegi C. (2011). Aflatoxin control and prevention strategies in key crops of Sub-Saharan Africa. *African Journal of Microbiology Research*.

[16] Tefera T., Mugo S., Likhayo P. (2011). Effects of insect population density and storage time on grain damage and weight loss in maize due to the maize weevil *Sitophilus zeamais* and the larger grain borer *Prostephanus truncatus*. *African Journal of Agricultural Research*.

[17] Boxall R. A (2002). Damage and loss caused by the larger grain borer. *Integrated Pest Management Reviews*.

[18] Kadjo D., Ricker-Gilbert J., Alexander C. (2016). Estimating price discounts for low-quality maize in sub-Saharan Africa: evidence from Benin. *World Development*.

[19] Magan N., Aldred D. (2007). Post-harvest control strategies: minimizing mycotoxins in the food chain. *International Journal of Food Microbiology*.

[20] Shephard G. S. (2008). Impact of mycotoxins on human health in developing countries. *Food Additives & Contaminants: Part A*.

[21] Matumba L., Sulyok M., Njoroge S. M. C. (2015). Uncommon occurrence ratios of aflatoxin B_1_, B_2_, G_1_, and G_2_ in maize and groundnuts from Malawi. *Mycotoxin Research*.

[22] Matumba L., Van Poucke C., Biswick T., Monjerezi M., Mwatseteza J., De Saeger S. (2014). A limited survey of mycotoxins in traditional maize based opaque beers in Malawi. *Food Control*.

[23] Kumar V., Basu M. S., Rajendran T. P. (2008). Mycotoxin research and mycoflora in some commercially important agricultural commodities. *Crop Protection*.

[24] Wagacha J. M., Muthomi J. W. (2008). Mycotoxin problem in Africa: current status, implications to food safety and health and possible management strategies. *International Journal of Food Microbiology*.

[25] Darwish W. S., Ikenaka Y., Nakayama S. M. M., Ishizuka M. (2014). An overview on mycotoxin contamination of foods in Africa. *Journal of Veterinary Medical Science*.

[26] Haladi S., Mycotoxin G. (2014). Why poultry breeders need to be concerned about emerging mycotoxins. *International Hatchery Practice*.

[27] Mboya R., Bogale A. (2012). Mycotoxin contamination of food and its possible implications on sustainable development in Rungwe district, Tanzania. *OIDA International Journal of Sustainable Development*.

[28] Owaga E., Muga R., Mumbo H., Aila F. (2011). Chronic dietary aflatoxins exposure in Kenya and emerging public health concerns of impaired growth and immune suppression in children. *International Journal of Biological and Chemical Sciences*.

[29] Kimanya M. E., Shirima C. P., Magoha H. (2014). Co-exposures of aflatoxins with deoxynivalenol and fumonisins from maize based complementary foods in Rombo, Northern Tanzania. *Food Control*.

[30] Kimanya M. E., De Meulenaer B., Tiisekwa B. (2008). Co-occurrence of fumonisins with aflatoxins in home-stored maize for human consumption in rural villages of Tanzania. *Food Additives & Contaminants: Part A*.

[31] Lewis L., Onsongo M., Njapau H. (2005). Aflatoxin contamination of commercial maize products during an outbreak of acute aflatoxicosis in eastern and central Kenya. *Environmental Health Perspectives*.

[32] Maina A. W., Wagacha J. M., Mwaura F. B., Muthomi J. W., Woloshuk C. P. (2016). Postharvest practices of maize farmers in Kaiti district, Kenya and the impact of hermetic storage on populations of *Aspergillus* spp. and aflatoxin contamination. *Journal of Food Research*.

[33] Daniel J. H., Lewis L. W., Redwood Y. A. (2011). Comprehensive assessment of maize aflatoxin levels in eastern Kenya, 2005–2007. *Environmental Health Perspectives*.

[34] Onemolease E. A., Aghanenu A. S., Adisa T. (2001). Effect of formal school education on farmers output and adoption of innovations: a case study of rubber farmers in Ikpoba-Okha local government area of Edo State. *Journal of Teacher Education*.

[35] Thamaga-Chitja J. M., Hendriks S. L., Ortmann G. F., Green M. (2004). Impact of maize storage on rural household food security in Northern Kwazulu-Natal. *Journal of Consumer Sciences*.

[36] AflaSTOP (2013). *Storage Surveys in Kenya: North Rift and Eastern*.

[37] Akowuah J. O., Lena D. M., Chian C., Anthony R. (2015). Effects of practices of maize farmers and traders in Ghana on contamination of maize by aflatoxins: case study of Ejura-Sekyeredumase municipality. *African Journal of Microbiology Research*.

[38] Midega C. A. O., Murage A. W., Pittchar J. O., Khan Z. R. (2016). Managing storage pests of maize: farmers’ knowledge, perceptions and practices in western Kenya. *Crop Protection*.

[39] Kaaya A. N., Kyamuhangire W. (2006). The effect of storage time and agroecological zone on mould incidence and aflatoxin contamination of maize from traders in Uganda. *International Journal of Food Microbiology*.

[40] Addo S., Birkinshaw L. A., Hodges R. J. (2002). Ten years after the arrival in Ghana of larger grain borer: farmers’ responses and adoption of IPM strategies. *International Journal of Pest Management*.

[41] Atukwase A., Kaaya A. N., Muyanja C. (2012). Dynamics of *Fusarium* and fumonisins in maize during storage–a case of the traditional storage structures commonly used in Uganda. *Food Control*.

[42] Potter N. N., Hotchkiss J. H. (1995). Milk and milk products. *Food Science*.

[43] Udoh J. M., Cardwell K. F., Ikotun T. (2000). Storage structures and aflatoxin content of maize in five agroecological zones of Nigeria. *Journal of Stored Products Research*.

[44] Kamala A., Kimanya M., Haesaert G. (2016). Local post-harvest practices associated with aflatoxin and fumonisin contamination of maize in three agro ecological zones of Tanzania. *Food Additives & Contaminants: Part A*.

[45] Netya W. (2017). *Farmers-Want-Government-To-Control-Consumption-Of-Green-Maize*.

[46] Mendoza J. R., Sabillón L., Martinez W., Campabadal C., Hallen-Adams H. E., Bianchini A. (2017). Traditional maize post-harvest management practices amongst smallholder farmers in Guatemala. *Journal of Stored Products Research*.

[47] Mwangi J. K., Mutungi C. M., Midingoyi S.-K. G., Faraj A. K., Affognon H. D. (2017). An assessment of the magnitudes and factors associated with postharvest losses in off-farm grain stores in Kenya. *Journal of Stored Products Research*.

[48] Hell K., Fandohan P., Ranajit B., Kiewnick S., Sikora R., Cotty P. J. (2008). Pre-and post-harvest management of aflatoxin in maize: an African perspective. *Mycotoxins: Detection Methods, Management, Public Health and Agricultural Trade*.

[49] Abass A. B., Ndunguru G., Mamiro P., Alenkhe B., Mlingi N., Bekunda M. (2014). Post-harvest food losses in a maize-based farming system of semi-arid Savannah area of Tanzania. *Journal of Stored Products Research*.

[50] Kitinoja L., Saran S., Roy S. K., Kader A. A. (2011). Postharvest technology for developing countries: challenges and opportunities in research, outreach and advocacy. *Journal of the Science of Food and Agriculture*.

[51] Kaminski J., Christiaensen L. (2014). Post-harvest loss in sub-Saharan Africa—what do farmers say?. *Global Food Security*.

[52] Affognon H., Mutungi C., Sanginga P., Borgemeister C. (2015). Unpacking postharvest losses in sub-Saharan Africa: a meta-analysis. *World Development*.

