# Supplementary material for: Postharvest Storage Practices of Maize in Rift Valley and Lower Eastern Regions of Kenya: A Cross-Sectional Study
Source: Int J Microbiol. 2020 Jan 31;2020:6109214. doi: 10.1155/2020/6109214 (PMC7016483; doi:10.1155/2020/6109214)
Supplement: Supplementary Materials — SPSS template of the raw data used in the manuscript. [file 6109214.f1.pdf]

id	qno	date	region	county	village	gps	direction	degree
150	35	2-May-17	Rift valley	Trans-nzoia	Sinendet	1.0177		
264	48	20-Apr-17	Lower East	Kitui	UAE	-1.80374	South	1
100	50	22-Apr-17	Rift valley	Nakuru	Saptet	-0.26169	South	0
1	1	29-Apr-17	Rift valley	Bomet	Kapchumbe	-0.88603	South	0
2	2	29-Apr-17	Rift valley	Bomet	Kapchumbe	-1.02864	South	1
9	9	29-Apr-17	Rift valley	Bomet	Kapchumbe	-0.94487	South	0
11	11	29-Apr-17	Rift valley	Bomet	Kapchumbe	-0.88606	South	0
12	12	29-Apr-17	Rift valley	Bomet	Kapchumbe	-0.87197	South	0
13	13	29-Apr-17	Rift valley	Bomet	Kapchumbe	-0.82735	South	0
15	15	29-Apr-17	Rift valley	Bomet	Kapchumbe	-0.83756	South	0
16	16	29-Apr-17	Rift valley	Bomet	Kapchumbe	-0.80862	South	0
17	17	29-Apr-17	Rift valley	Bomet	Kapchumbe	-0.8443	South	0
19	19	29-Apr-17	Rift valley	Bomet	Kapchumbe	-0.87268	South	0
20	20	29-Apr-17	Rift valley	Bomet	Kapchumbe	-0.94868	South	0
25	25	30-Apr-17	Rift valley	Bomet	Chesambai	-0.96405	South	0
26	26	30-Apr-17	Rift valley	Bomet	Chesambai	-0.77989	South	0
27	27	30-Apr-17	Rift valley	Bomet	Chesambai	-0.96405	South	0
29	29	30-Apr-17	Rift valley	Bomet	Chesambai	-0.92295	South	0
31	31	1-May-17	Rift valley	Bomet	Kapolesobe	-0.96134	South	0
35	35	1-May-17	Rift valley	Bomet	Kapolesobe	-0.91222	South	0
36	36	1-May-17	Rift valley	Bomet	Kapolesobe	-0.91821	South	0
38	38	1-May-17	Rift valley	Bomet	Kapolesobe	-0.94139	South	0
39	39	1-May-17	Rift valley	Bomet	Kapolesobe	-0.93933	South	0
42	42	1-May-17	Rift valley	Bomet	Kaplombe	-0.83735	South	0
43	43	1-May-17	Rift valley	Bomet	Kaplombe	-0.87197	South	0
46	46	29-Apr-17	Rift valley	Bomet	Kapchumbe	-0.84435	South	0
78	28	22-Apr-17	Rift valley	Nakuru	Saptet	-0.26099	South	0
82	32	22-Apr-17	Rift valley	Nakuru	Saptet	-0.26037	South	0
95	45	22-Apr-17	Rift valley	Nakuru	Saptet	-0.26357		
99	49	22-Apr-17	Rift valley	Nakuru	Saptet	-0.26158	South	0
115	15	1-May-17	Rift valley	Nakuru	Chepseon	-0.20498	South	0
119	4	2-May-17	Rift valley	Trans-nzoia	Chepkaitit	1.00604	North	1
126	11	2-May-17	Rift valley	Trans-nzoia	Chepkaitit	1.00533	North	1
130	15	2-May-17	Rift valley	Trans-nzoia	Chepkaitit	1.00223	North	1
141	26	2-May-17	Rift valley	Trans-nzoia	Chepkaitit	1.00486	North	1
151	36	2-May-17	Rift valley	Trans-nzoia	Sinendet	1.01799		
161	46	2-May-17	Rift valley	Trans-nzoia	Laboot	1.01203	North	1
3	3	29-Apr-17	Rift valley	Bomet	Kapchumbe	-1.00305	South	1
5	5	29-Apr-17	Rift valley	Bomet	Kapchumbe	-0.97369	South	0
6	6	29-Apr-17	Rift valley	Bomet	Kapchumbe	-0.97758	South	0
7	7	29-Apr-17	Rift valley	Bomet	Kapchumbe	-0.9521	South	0
8	8	29-Apr-17	Rift valley	Bomet	Kapchumbe	-0.9474	South	0
10	10	29-Apr-17	Rift valley	Bomet	Kapchumbe	-0.92506	South	0
14	14	29-Apr-17	Rift valley	Bomet	Kapchumbe	-0.886	South	0
18	18	29-Apr-17	Rift valley	Bomet	Kapchumbe	-0.88492	South	0
21	21	30-Apr-17	Rift valley	Bomet	Chesambai	-0.83735	South	0

22	22	30-Apr-17	Rift valley	Bomet	Chesambai	-0.96405	South		0
23	23	30-Apr-17	Rift valley	Bomet	Chesambai	-0.886	South		0
24	24	30-Apr-17	Rift valley	Bomet	Chesambai	-0.96134	South		5
28	28	30-Apr-17	Rift valley	Bomet	Chesambai	-0.94239	South		0
30	30	30-Apr-17	Rift valley	Bomet	Chesambai	-0.94868	South		0
33	33	1-May-17	Rift valley	Bomet	Kaplombe	-0.9986	South		0
34	34	1-May-17	Rift valley	Bomet	Kapolesobe	-0.96405	South		0
41	41	1-May-17	Rift valley	Bomet	Kaplombe	-96155	South		0
44	44	1-May-17	Rift valley	Bomet	Kaplombe	-0.78333	South		0
53	3	22-Apr-17	Rift valley	Nakuru	Waldai	-0.25122	South		0
76	26	22-Apr-17	Rift valley	Nakuru	Waldai	-0.2629	South		0
81	31	22-Apr-17	Rift valley	Nakuru	Saptet	-0.25941	South		0
104	4	29-Apr-17	Rift valley	Nakuru	Chepseon	-0.20391	South		0
107	7	29-Apr-17	Rift valley	Nakuru	Chepseon	-0.20461	South		0
108	8	29-Apr-17	Rift valley	Nakuru	Chepseon	-0.20452	South		0
109	9	30-Apr-17	Rift valley	Nakuru	Chepseon	-0.20668	South		0
120	5	2-May-17	Rift valley	Trans-nzoia	Chepkaitit	1.00604	North		1
121	6	2-May-17	Rift valley	Trans-nzoia	Chepkaitit	1.0005	North		1
127	12	2-May-17	Rift valley	Trans-nzoia	Chepkaitit	1.00508	North		1
128	13	2-May-17	Rift valley	Trans-nzoia	Chepkaitit	1.00535	North		1
132	17	2-May-17	Rift valley	Trans-nzoia	Chepkaitit	1.00235	North		1
134	19	2-May-17	Rift valley	Trans-nzoia	Chepkaitit	1.00205	North		1
137	22	2-May-17	Rift valley	Trans-nzoia	Chepkaitit	1.00274	North		1
138	23	2-May-17	Rift valley	Trans-nzoia	Chepkaitit	1.00092	North		1
142	27	2-May-17	Rift valley	Trans-nzoia	Sinendet	1.01715			
144	29	2-May-17	Rift valley	Trans-nzoia	Sinendet	1.01484			
145	30	2-May-17	Rift valley	Trans-nzoia	Sinendet	1.01574			
146	31	2-May-17	Rift valley	Trans-nzoia	Sinendet	1.0161			
147	32	2-May-17	Rift valley	Trans-nzoia	Sinendet	1.01664			
152	37	2-May-17	Rift valley	Trans-nzoia	Sinendet				
153	38	2-May-17	Rift valley	Trans-nzoia	Sinendet				
154	39	2-May-17	Rift valley	Trans-nzoia	Laboot	1.01197			
155	40	2-May-17	Rift valley	Trans-nzoia	Laboot	1.0103	North		1
156	41	2-May-17	Rift valley	Trans-nzoia	Laboot	1.01087	North		1
157	42	2-May-17	Rift valley	Trans-nzoia	Laboot	1.01092	North		1
158	43	2-May-17	Rift valley	Trans-nzoia	Laboot	1.01129	North		1
159	44	2-May-17	Rift valley	Trans-nzoia	Laboot	1.01181	North		1
165	50	2-May-17	Rift valley	Trans-nzoia	Laboot	1.01131	North		1
254	38	20-Apr-17	Lower East	Kitui	UAE	-1.80272	South		1
219	2	20-Apr-17	Lower East	Kitui	Kiteta	-1.75619			
231	14	20-Apr-17	Lower East	Kitui	Kiteta	-1.75428			
221	4	20-Apr-17	Lower East	Kitui	Kiteta	-1.7542			
171	4	########	Lower East	Machakos	Uvalini	-1.40416	South		1
173	6	########	Lower East	Machakos	Uvalini	-1.48651	South		1
175	8	########	Lower East	Machakos	Uvalini	-0.1415	South		1
189	22	########	Lower East	Machakos	Uvalini		South		1
191	24	########	Lower East	Machakos	Uvalini		South		1

201	37	########	Lower East Machakos	Kithima	-1.40416	South	1
202	38	########	Lower East Machakos	Kithima	-1.4058	South	1
208	44	########	Lower East Machakos	Kithima	-1.40378	South	1
209	45	########	Lower East Machakos	Kithima	-1.4048	South	1
212	48	########	Lower East Machakos	Kithima	-1.40285	South	1
213	49	########	Lower East Machakos	Kithima	-1.39178	South	1
226	9	20-Apr-17	Lower East Kitui	Kiteta	-17567		
232	15	20-Apr-17	Lower East Kitui	Kiteta	-1.75261		
233	17	20-Apr-17	Lower East Kitui	Kiteta	-1.75385		
235	19	20-Apr-17	Lower East Kitui	Kiteta	-1.75607		
236	20	20-Apr-17	Lower East Kitui	Kiteta	-1.75288		
243	27	20-Apr-17	Lower East Kitui	Kiteta	-1.80142	South	1
244	28	20-Apr-17	Lower East Kitui	UAE			
247	31	20-Apr-17	Lower East Kitui	UAE			
249	33	20-Apr-17	Lower East Kitui	UAE	-1.80365	South	1
250	34	20-Apr-17	Lower East Kitui	UAE	-1.83645	South	1
253	37	20-Apr-17	Lower East Kitui	UAE	-1.8491	South	1
258	42	20-Apr-17	Lower East Kitui	UAE	-1.80252	South	1
259	43	20-Apr-17	Lower East Kitui	UAE			
260	44	20-Apr-17	Lower East Kitui	UAE			
261	45	20-Apr-17	Lower East Kitui	UAE			
262	46	20-Apr-17	Lower East Kitui	UAE			
265	49	20-Apr-17	Lower East Kitui	UAE			
271	33	18-Apr-17	Lower East Makueni	Kiatine	-1.761		
273	2	18-Apr-17	Lower East Makueni	Nthangu	-1.76027	South	1
282	15	18-Apr-17	Lower East Makueni	Nthangu	-1.75858	South	1
293	10	18-Apr-17	Lower East Makueni	Thangu	-1.75978	South	1
295	13	18-Apr-17	Lower East Makueni	Kyumu	-1.74074		
296	26	18-Apr-17	Lower East Makueni	Kyumu	-1.78235		
298	28	18-Apr-17	Lower East Makueni	Kyumu	-1.76819		
303	45	18-Apr-17	Lower East Makueni	Kyumu	-1.76243		
304	46	18-Apr-17	Lower East Makueni	Kyumu	-1.7934		
315	29	18-Apr-17	Lower East Makueni	Kisyungii	-1.75821		
169	2	########	Lower East Machakos	Kithima	-1.40496	South	1
177	10	########	Lower East Machakos	Mithini	-1.39164	South	1
178	11	########	Lower East Machakos	Mithini	-1.39556	South	1
179	12	########	Lower East Machakos	Mithini	-1.39164	South	1
180	13	########	Lower East Machakos	Uvalini		South	1
181	14	########	Lower East Machakos	Uvalini		South	1
182	15	########	Lower East Machakos	Uvalini		South	1
183	16	########	Lower East Machakos	Uvalini		South	1
184	17	########	Lower East Machakos	Uvalini		South	1
185	18	########	Lower East Machakos	Uvalini		South	1
186	19	########	Lower East Machakos	Mithini		South	1
187	20	########	Lower East Machakos	Mithini		South	1
188	21	########	Lower East Machakos	Mithini		South	1
190	23	########	Lower East Machakos	Uvalini		South	1

193	26	########	Lower East Machakos	Kithima	-1.40389	South	1
194	27	########	Lower East Machakos	Mithini	1.392	South	1
195	28	########	Lower East Machakos	Mithini	-1.39228	South	1
196	29	########	Lower East Machakos	Mithini	-1.39524	South	1
198	34	########	Lower East Machakos	Mithini	-1.39584	South	1
199	35	########	Lower East Machakos	Mithini	-1.39578	South	1
200	36	########	Lower East Machakos	Mithini	-1.38888	South	1
203	39	########	Lower East Machakos	Kithima	-1.40426	South	1
204	40	########	Lower East Machakos	Kithima	-1.4042	South	1
205	41	########	Lower East Machakos	Kithima	-1.40568	South	1
206	42	########	Lower East Machakos	Kithima	-1.4059	South	1
210	46	########	Lower East Machakos	Kithima	-1.40694	South	1
211	47	########	Lower East Machakos	Kithima	-1.40357	South	1
214	50	########	Lower East Machakos	Mithini		South	1
215	51	########	Lower East Machakos	Mithini		South	
216	52	########	Lower East Machakos	Mithini		South	1
217	53	########	Lower East Machakos	Mithini		South	1
218	1	20-Apr-17	Lower East Kitui	Kiteta	-1.75421		
220	3	20-Apr-17	Lower East Kitui	Kiteta	-1.75685		
222	5	20-Apr-17	Lower East Kitui	Kiteta	-1.75618		
223	6	20-Apr-17	Lower East Kitui	Kiteta	-1.75617		
224	7	20-Apr-17	Lower East Kitui	Kiteta	-1.75616		
225	8	20-Apr-17	Lower East Kitui	Kiteta	-1.75614		
227	10	20-Apr-17	Lower East Kitui	Kiteta	-1.75613		
228	11	20-Apr-17	Lower East Kitui	Kiteta	-1.75284		
229	12	20-Apr-17	Lower East Kitui	Kiteta	-1.7561		
230	13	20-Apr-17	Lower East Kitui	Kiteta	-1.75609		
234	18	20-Apr-17	Lower East Kitui	Kiteta	-1.75286		
237	21	20-Apr-17	Lower East Kitui	Kiteta	-1.75298		
238	22	20-Apr-17	Lower East Kitui	kiteta	-1.75406		
239	23	20-Apr-17	Lower East Kitui	Kiteta	-1.75302		
240	24	20-Apr-17	Lower East Kitui	Kiteta	-1.75326		
242	26	20-Apr-17	Lower East Kitui	Kiteta	-1.75606	South	1
246	30	20-Apr-17	Lower East Kitui	UAE			
248	32	20-Apr-17	Lower East Kitui	UAE			
251	35	20-Apr-17	Lower East Kitui	UAE	-1.84921	South	1
252	36	20-Apr-17	Lower East Kitui	UAE	-1.79282	South	1
255	39	20-Apr-17	Lower East Kitui	UAE	-1.825	South	1
256	40	20-Apr-17	Lower East Kitui	UAE	-1.83645	South	1
257	41	20-Apr-17	Lower East Kitui	UAE			
267	51	20-Apr-17	Lower East Kitui	UAE			
268	52	20-Apr-17	Lower East Kitui	UAE	-1.82491	South	1
272	1	18-Apr-17	Lower East Makueni	Nthangu	-1.75892	South	1
274	3	18-Apr-17	Lower East Makueni	Nthangu	-1.76023	South	1
275	4	18-Apr-17	Lower East Makueni	Nthangu	-1.75772	South	1
276	5	18-Apr-17	Lower East Makueni	Nthangu	-1.75702	South	1
277	6	18-Apr-17	Lower East Makueni	Nthangu	-1.75704	South	1

278	7	18-Apr-17	Lower East	Makueni	Nthangu	-1.75966	South	1
279	11	18-Apr-17	Lower East	Makueni	Nthangu	-1.76057	South	1
280	13	18-Apr-17	Lower East	Makueni	Nthangu	-1.75905	South	1
281	14	18-Apr-17	Lower East	Makueni	Nthangu	-1.75895	South	1
283	16	18-Apr-17	Lower East	Makueni	Nthangu	-1.75977	South	1
285	18	18-Apr-17	Lower East	Makueni	Nthangu	-1.75982	South	1
286	19	18-Apr-17	Lower East	Makueni	Nthangu	-1.76176	South	1
287	20	18-Apr-17	Lower East	Makueni	Nthangu	-1.76182	South	1
288	21	18-Apr-17	Lower East	Makueni	Nthangu	-1.76242	South	1
289	22	18-Apr-17	Lower East	Makueni	Nthangu	-1.76286	South	1
290	23	18-Apr-17	Lower East	Makueni	Nthangu	-1.76319	South	1
292	9	18-Apr-17	Lower East	Makueni	Thangu	-1.7593	South	1
294	12	18-Apr-17	Lower East	Makueni	Thangu	-1.75974	South	1
297	27	18-Apr-17	Lower East	Makueni	Kyumu	-1.78256		
299	29	18-Apr-17	Lower East	Makueni	Kyumu	-1.76485		
300	42	18-Apr-17	Lower East	Makueni	Kyumu	-1.78235		
302	44	18-Apr-17	Lower East	Makueni	Kyumu	-1.76245		
305	47	18-Apr-17	Lower East	Makueni	Kyumu	-1.78351		
306	48	18-Apr-17	Lower East	Makueni	Kyumu	-1.7835		
307	49	18-Apr-17	Lower East	Makueni	Kyumu			
308	50	18-Apr-17	Lower East	Makueni	Kyumu	-1.74184		
310	8	18-Apr-17	Lower East	Makueni	Kisyongai	-1.781		
312	35	18-Apr-17	Lower East	Makueni	Kisyongai	-1.77847		
313	1	18-Apr-17	Lower East	Makueni	Kisyungii			
314	5	18-Apr-17	Lower East	Makueni	Kisyungii	-1.7528		
316	36	18-Apr-17	Lower East	Makueni	Kisyungii	-1.781		
317	37	18-Apr-17	Lower East	Makueni	Kisyungii	-2.0655		
318	38	18-Apr-17	Lower East	Makueni	Kisyungii	-1.76555		
197	33	########	Lower East	Machakos	Mithini		South	
68	18	22-Apr-17	Rift valley	Nakuru	Waldai	-0.24926	South	0
52	2	22-Apr-17	Rift valley	Nakuru	Waldai	-0.25023	South	0
57	7	22-Apr-17	Rift valley	Nakuru	Waldai	-0.24782	South	0
70	20	22-Apr-17	Rift valley	Nakuru	Waldai	-0.24867	South	0
74	24	22-Apr-17	Rift valley	Nakuru	Waldai	-2.24852	South	0
80	30	22-Apr-17	Rift valley	Nakuru	Saptet	-0.25847	South	0
87	37	22-Apr-17	Rift valley	Nakuru	Saptet	-0.2602	South	0
102	2	28-Apr-17	Rift valley	Nakuru	Chepseon	-0.20697	South	0
112	12	1-May-17	Rift valley	Nakuru	Chepseon	-0.20823	South	0
114	14	1-May-17	Rift valley	Nakuru	Chepseon	-0.20313	South	0
192	25	########	Lower East	Machakos	Kithima	-1.40422	South	1
32	32	2-May-17	Rift valley	Bomet	Kapolesobe	-0.92294	South	0
67	17	22-Apr-17	Rift valley	Nakuru	Waldai	-0.24888	South	0
75	25	22-Apr-17	Rift valley	Nakuru	Waldai	-0.25111	South	0
162	47	2-May-17	Rift valley	Trans-nzoia	Laboot	1.01302		
164	49	2-May-17	Rift valley	Trans-nzoia	Laboot	1.01134	North	1
263	47	20-Apr-17	Lower East	Kitui	UAE	-1.82491	South	1
92	42	22-Apr-17	Rift valley	Nakuru	Saptet	-0.25916	South	0

50	50	29-Apr-17	Rift valley	Bomet	Chesambai	-0.8653		
60	10	22-Apr-17	Rift valley	Nakuru	Waldai	-0.24829	South	0
69	19	22-Apr-17	Rift valley	Nakuru	Waldai	-0.24952	South	0
101	1	28-Apr-17	Rift valley	Nakuru	Chepseon	-0.20516	South	0
105	5	29-Apr-17	Rift valley	Nakuru	Chepseon	-0.20342	South	0
110	10	1-May-17	Rift valley	Nakuru	Chepseon	-0.20725	South	0
133	18	2-May-17	Rift valley	Trans-nzoia	Chepkaitit	1.00397	North	1
117	2	2-May-17	Rift valley	Trans-nzoia	Chepkaitit	1.01604	North	1
71	21	22-Apr-17	Rift valley	Nakuru	Waldai	-0.24954	South	0
85	35	22-Apr-17	Rift valley	Nakuru	Saptet	-0.26026	South	5
89	39	22-Apr-17	Rift valley	Nakuru	Saptet	-0.26226	South	0
4	4	29-Apr-17	Rift valley	Bomet	Kapchumbe	-0.99871	South	0
64	14	22-Apr-17	Rift valley	Nakuru	Waldai	-0.2497	South	0
84	34	22-Apr-17	Rift valley	Nakuru	Saptet	-0.26078	South	0
125	10	2-May-17	Rift valley	Trans-nzoia	Chepkaitit	1.00604	North	1
135	20	2-May-17	Rift valley	Trans-nzoia	Chepkaitit	1.00456	North	1
160	45	2-May-17	Rift valley	Trans-nzoia	Laboot	1.01134	North	1
163	48	2-May-17	Rift valley	Trans-nzoia	Laboot	1.01207	North	1
207	43	########	Lower East	Machakos	Kithima	-1.40632	South	1
301	43	18-Apr-17	Lower East	Makueni	Kyumu	-1.76649		
47	47	29-Apr-17	Rift valley	Bomet	Chesambai	-0.8653		
63	13	22-Apr-17	Rift valley	Nakuru	Waldai			
66	16	22-Apr-17	Rift valley	Nakuru	Waldai	-0.24905	South	0
91	41	22-Apr-17	Rift valley	Nakuru	Saptet	-0.2576	South	0
98	48	22-Apr-17	Rift valley	Nakuru	Waldai	-0.25215	South	0
131	16	2-May-17	Rift valley	Trans-nzoia	Chepkaitit	1.00213	North	1
140	25	2-May-17	Rift valley	Trans-nzoia	Chepkaitit	1.00472	North	1
174	7	########	Lower East	Machakos	Mithini	-1.39163	South	1
116	1	2-May-17	Rift valley	Trans-nzoia	Chepkaitit	1.04605	North	1
37	37	1-May-17	Rift valley	Bomet	Kapolesobe	-0.93177	South	0
40	40	1-May-17	Rift valley	Bomet	Kapolesobe	-0.92421	South	0
168	1	########	Lower East	Machakos	Nzoweni	-1.48543	South	1
176	9	########	Lower East	Machakos	Mithini	-1.39209	South	1
319	39	18-Apr-17	Lower East	Makueni	Kisyungii	-1.74014		
73	23	22-Apr-17	Rift valley	Nakuru	Waldai	-0.25021	South	0
77	27	22-Apr-17	Rift valley	Nakuru	Saptet	-0.25225	South	0
129	14	2-May-17	Rift valley	Trans-nzoia	Chepkaitit	1.00491	North	1
79	29	22-Apr-17	Rift valley	Nakuru	Saptet	-0.25548	South	0
88	38	22-Apr-17	Rift valley	Nakuru	Saptet	-0.25424	South	0
94	44	22-Apr-17	Rift valley	Nakuru	Saptet	-0.25946	South	5
48	48	29-Apr-17	Rift valley	Bomet	Chesambai	-0.8553		
54	4	22-Apr-17	Rift valley	Nakuru	Waldai	-0.2506	South	0
61	11	22-Apr-17	Rift valley	Nakuru	Waldai	-0.24754	South	0
65	15	22-Apr-17	Rift valley	Nakuru	Waldai	-0.25017	South	0
86	36	22-Apr-17	Rift valley	Nakuru	Saptet	-0.22143		
93	43	22-Apr-17	Rift valley	Nakuru	Saptet	-0.2629	South	0
106	6	29-Apr-17	Rift valley	Nakuru	Chepseon	-0.20291	South	0

113	13	29-Apr-17	Rift valley	Nakuru	Chepseon	-0.20938	South	0
136	21	2-May-17	Rift valley	Trans-nzoia	Chepkaitit	1.00197	North	1
149	34	2-May-17	Rift valley	Trans-nzoia	Sinendet	1.01736		
270	32	18-Apr-17	Lower East	Makueni	Kiatine	-1.781		
55	5	22-Apr-17	Rift valley	Nakuru	Waldai	-0.25024	South	0
111	11	1-May-17	Rift valley	Nakuru	Chepseon	-0.20786	South	0
122	7	2-May-17	Rift valley	Trans-nzoia	Chepkaitit	1.00668	North	1
49	49	29-Apr-17	Rift valley	Bomet	Chesambai	-0.8643		
59	9	22-Apr-17	Rift valley	Nakuru	Waldai	-0.2482	South	0
143	28	2-May-17	Rift valley	Trans-nzoia	Laboot	1.01257		
148	33	2-May-17	Rift valley	Trans-nzoia	Sinendet	1.01705		
90	40	22-Apr-17	Rift valley	Nakuru	Saptet	-0.25546	South	0
170	3	########	Lower East	Machakos	Mithin	-1.39355	South	1
83	33	22-Apr-17	Rift valley	Nakuru	Saptet	-2.6085	South	0
45	45	1-May-17	Rift valley	Bomet	Kaplombe	-0.7816	South	0
309	31	18-Apr-17	Lower East	Makueni	Kisionge	-1.75631		
172	5	########	Lower East	Machakos	Mithini	-1.3924	South	1
266	50	20-Apr-17	Lower East	Kitui	UAE	-1.80414	South	1
284	17	18-Apr-17	Lower East	Makueni	Nthangu	-1.75923	South	1
291	8	18-Apr-17	Lower East	Makueni	Thangu	-1.75925	South	1
311	34	18-Apr-17	Lower East	Makueni	Kisyongai			
96	46	22-Apr-17	Rift valley	Nakuru	Saptet	-0.26232	South	0
97	47	22-Apr-17	Rift valley	Nakuru	Saptet	-0.2618	South	0
103	3	28-Apr-17	Rift valley	Nakuru	Chepseon	-0.20546	South	0
51	1	22-Apr-17	Rift valley	Nakuru	Waldai	-0.25143	South	0
56	6	22-Apr-17	Rift valley	Nakuru	Waldai	-0.2477	South	0
58	8	22-Apr-17	Rift valley	Nakuru	Waldai	-0.24804	South	0
62	12	22-Apr-17	Rift valley	Nakuru	Waldai	-0.24831	South	0
72	22	22-Apr-17	Rift valley	Nakuru	Waldai	-0.24977	South	0
118	3	2-May-17	Rift valley	Trans-nzoia	Chepkaitit	1.01513	North	1
123	8	2-May-17	Rift valley	Trans-nzoia	Chepkaitit	1.00787	North	1
166	51	2-May-17	Rift valley	Trans-nzoia	Laboot	1.00912	North	1
167	52	2-May-17	Rift valley	Trans-nzoia	Laboot	1.0095	North	1

gprsmin	gpsdoubleł	VAR00012	VAR00013	VAR00014	VAR00015	VAR00016	age	gender
		34.90983					43	Male
48	13.212	38.17234	East	38	10	20.418	37	Female
15	41.61	35.83393	East	35	50	2.136	27	Female
53	9.71412	35.20357	East	35	12	12.85452	34	Male
1	43.104	35.08676	East	35	5	12.35328	32	Female
56	55.23612	35.16771	East	35	10	3.747	38	Female
53	9.83292	35.20358	East	35	12	12.8916	25	Female
52	19.08552	35.23321	East	35	13	59.57148	32	Male
50	14.46576	35.09753	East	35	5	51.12564	53	Female
50	14.47872	35.09753	East	35	5	51.10548	33	Female
48	31.02588	35.18686	East	35	11	12.70356	36	Male
50	39.46848	35.09952	East	35	5	58.26624	30	Female
52	34.62564	35.22345	East	35	13	4.42288	27	Female
56	55.23612	35.16771	East	35	10	3.747	59	Female
57	50.5012	35.1225	East	35	7	20.09064	27	Male
46	47.6184	35.22194	East	35	13	18.98256	48	Female
57	50.5782	35.19029	East	35	11	25.05576	34	Male
55	22.61136	35.12246	East	35	7	20.8542	39	Female
57	40.8384	35.19273	East	35	11	33.81144	60	Male
54	43.97508	35.20348	East	35	12	12.52404	33	Male
55	5.56104	36.15993	East	35	9	35.76096	59	Female
56	29.01984	35.15268	East	35	9	9.65376	65	Female
56	21.59304	35.16046	East	35	9	37.6639	28	Female
59	14.47872	35.09753	East	35	5	57.10548	27	Male
52	19.08552	35.23321	East	35	13	59.57148	24	Female
50	39.6636	35.09956	East	35	11	12.7288	28	Male
15	39.57	35.83457	East	35	50	4.458	42	Male
15	37.326	35.83244	East	35	49	56.778	25	Female
		35.83371					39	Female
15	4.688	35.83415	East	35	50	2.934	54	Female
12	17	35.83401	East	35	50	2	63	Female
0	21.738	34.92139	East	34	55	16.932	41	Female
0	19.182	34.9211	East	34	55	15.966	36	Male
0	8.02162	34.92374	East	34	55	25.93576	58	Male
0	17.48872	34.92205	East	34	55	19.5915	25	Female
		34.9065					27	Male
0	43.296	34.91682	East	34	55	0.558	40	Female
0	10.97028	35.11092	East	35	6	39.32244	43	Female
58	25.2948	35.14037	East	35	8	25.34316	52	Female
58	39.27	35.08648	East	35	8	9.55284	57	Male
57	7.57008	35.16105	East	35	9	39.78324	53	Female
56	50.91468	35.16576	East	35	9	56.72376	50	Male
55	30.21528	35.19691	East	35	11	48.86664	22	Male
53	9.58272	35.20355	East	35	12	12.7668	32	Male
53	5.70624	35.20392	East	35	12	14.1246	29	Female
50	14.46576	35.09753	East	35	5	51.12564	42	Male

57	50.5782	35.19029	East	35	11	25.05516	34	Female
53	9.58272	35.20355	East	35	12	12.76668	29	Male
57	40.8384	35.19273	East	35	11	33.81144	49	Female
56	32.61624	35.15535	East	35	9	19.24884	34	Female
56	55.3742	35.1227	East	35	8	9.3743	42	Female
59	54.96648	35.11311	East	35	6	47.19816	37	Female
57	50.5782	35.19029	East	35	11	25.05576	22	Female
57	41.56452	35.19307	East	35	11	35.0592	64	Female
46	59.9736	35.2775	East	35	16	38.99172	26	Male
17	2.431	35.62991		35	49	45.106	35	Female
15	46.44	35.83352	East	35	50	0.666	30	Female
15	33.87	35.83232	East	35	49	56.346	25	Female
12	14	35.83233	East	35	49	56	44	Female
12	16	35.83293	East	35	49	58	74	Female
12	15	35.83348	East	35	50	0	37	Female
12	24	35.83106	East	35	49	51	32	Male
0	21.732	34.92136	East	34	55	16.902	18	Male
0	1.818	34.92878	East	34	55	42.18	32	Female
0	18.306	34.92164	East	34	55	17.922	31	Female
0	19.25998	34.92282	East	34	55	22.16949	51	Female
0	8.46018	34.92349	East	34	55	24.51986	70	Female
0	7.38	34.92411	East	34	55	26.808	69	Female
0	9.85588	34.92269	East	34	55	21.69931	37	Female
0	3.92435	34.92435	East	34	55	29.66256	54	Female
		34.9135					37	Male
		34.9115					35	Female
		34.91114					37	Male
		34.91096					44	Male
		34.91063					40	Male
							45	Male
							50	Male
		34.91242					46	Female
0	37.098	34.91799	East	34	55	4.77	65	Female
0	39.12	34.91772	East	34	55	3.798	27	Male
0	39.324	34.41724	East	34	55	2.076	33	Male
0	40.632	34.9174	East	34	55	2.172	33	Female
0	42.516	34.91736	East	34	55	2.484	45	Male
0	39.48	34.9205	East	34	55	13.794	50	Male
48	9.798	38.16785	East	38	10	4.266	49	Female
		38.1076					65	Female
		38.10665					43	Female
		38.1064					32	Female
24	14.964	37.24895	East	37	14	36.232	46	Female
30	7.81	37.26015	East	37	15	38.433	73	Male
24	18.949	37.27117	East	37	21	4.514	65	Male
30	6.21864		East	37	15	23.24376	39	Female
30	6.21864		East	37	15	23.24376	55	Female

24	14.838		East	37	15	12.114	91	Female
24	20.88		East	37	15	9.522	50	Female
24	13.62	37.25245	East	37	15	10.632	28	Female
24	28.134		East	37	26	11.844	26	Female
24	10	2.722	East	37	15	8.718	30	Female
23	30.44	37.25172	East	37	15	6.142	33	Male
		38.10762					26	Male
		38.10532					35	Male
		38.1064					37	Female
		38.10754					41	Male
		38.10552					61	Female
45	18	38.15739	East	38	9	26.598	33	Male
							35	Female
							44	Female
48	14.886	38.6535	East	38	9	55.48	80	Female
50	11.22684	38.19915	East	38	11	56.93892	82	Female
50	57.16716	38.20754	East	38	12	27.35	18	Female
48	9.096	38.16768	East	38	10	3.63	21	Male
							42	Female
							21	Male
							32	Female
							18	Female
							25	Female
		42.6402					35	Male
45	36.972	37.61088	East	37	36	39.174	65	Female
45	30.876	37.61594	East	37	36	57.402	43	Male
45	35.208	37.61384	East		37.36	49.806	26	Male
		37.58128	East				19	Female
		37.62151					73	Male
		37.55459					60	Male
		37.66252					50	Female
		37.6521					65	Female
		37.56549					40	Female
24	17.868	37.25117	East	37	15	4.218	32	Male
23	29.91	37.25267	East	37	15	9.577	28	Female
23	44.016	37.25214	East	37	15	7.11	29	Female
23	29.91	37.25266	East	37	15	9.588	34	Female
30	6.21864	37.15				15.24376	70	Male
30	16.2186		East	37		15.24376	60	Female
30	6		East	37	15	23	58	Female
30	6.21864		East	37	15	23	60	Female
30	6.21864		East	37	15	23	28	Female
30	6		East	37	15	23	28	Male
30	6.21864		East	37	15	23	46	Female
30	6.21864		East	37	15	23.2476	54	Female
30	6.21864		East	37	15	23.24376	65	Female
30	6		East	37	15	23	27	Female

24	13.992	37.25181	East	37	15	6.504	28	Female
23	31.194	37.25194	East	37	15	6.996	35	Male
23	32.208	37.25153	East	37	15	5.496	28	Male
23	42.882	37.25199	East	37	15	7.17	70	Male
23	45.904	37.25206	East	37	15	7.484	35	Female
23	44.826	37.25202	East	37	15	7.254	70	Male
23	19.98	37.24363	East	37	14	37.011	29	Female
24	15.3	37.25346	East	37	15	12.444	65	Male
24	15.12	37.2303	East	37	15	10.902	55	Male
24	20.466	37.25252	East	37	15	4.762	34	Male
24	21.234	37.25234	East	37	15	8.24	36	Male
24	24.972		East	37	15	2.208	36	Female
24	12.84		East	37	15	8.146	70	Male
30	6.21864		East	37	15	23.24316	63	Female
							33	Female
30	6.21864		East	37.1523			34	Female
31	6.3151		East	37.1824			39	Male
		38.1066					68	Female
		38.10769					45	Female
		38.10756					30	Female
		38.10755					42	Male
		38.10756					39	Male
		38.10755					33	Male
		38.10755					38	Female
		38.10471					32	Male
		38.10755					80	Male
		38.10755					60	Male
		38.10545					82	Male
		38.10575					53	Female
		38.10653					46	Female
		38.10581					30	Male
		38.1059					23	Male
45	21.834	38.10759	East	38	6	27.318	71	Male
							40	Male
							30	Female
50	57.17	38.20759	East	38	12	27.33	27	Female
47	34.146				38.16	24	31	Male
49	29.68	38.1899	East	38	11	23.62	27	Male
50	11.227	38.14915	East	38	11	56.94	37	Female
							22	Male
							66	Female
49	29.68428	38.18989	East	38	11	23.6202	30	Female
45	32.118	37.60972	East	37	36	2	45	Female
45	37.494	37.61132	East	37	36	40.788	40	Female
45	27.788	37.61202		37	36	43.278	45	Male
45	25.44	37.61252	East	37	36	45.084	22	Female
45	25.332	37.61247	East		37.36	44.796	26	Female

45	34.782	37.61226	East	37	36	44.148	55	Male
45	38.136	37.61501	East	37	36	54.042	53	Female
45	32.592	37.61552	East	37	36	55.812	86	Female
45	32.208	37.6162	East	37	36	58.308	64	Male
45	35.172	37.61533	East	37	36	55.248	45	Female
45	35.382	37.61531	East	3	36	55.104	23	Male
45	42.288	37.61545	East	37	36	5.626	65	Female
45	45.9	37.61524	East	37	36	54.984	27	Female
45	44.694	37.61496	East	37	36	53.874	32	Female
45	46.278	37.61448	East	37	36	52.128	38	Female
45	47.99	37.61507	East	37	36	54.114	38	Female
45	33.48	37.61375	East	37	36	49.488	30	Male
45	35.082	37.615	East	37	36	54.006	44	Female
		37.63162					41	Female
		37.58482					30	Female
		37.62151					35	Female
		37.63456					45	Male
		37.63215					35	Female
		37.6223					46	Male
							26	Male
		37.56134					37	Female
		37.62146					52	Male
		37.59807					65	Male
							59	Male
		37.58305					34	Male
		37.62146					29	Female
		37.56527					40	Female
		37.56527					89	Male
							35	Female
14	57.546	35.82878	East	35	49	43.602	18	Female
15	0.834	35.82998		35	49	47.94	51	Male
14	51.846	35.82936		35	49	45.678	44	Male
14	55.386	35.82898	East	35	49	44.346	31	Female
14	54.672	35.82905	East	35	49	44.586	47	Male
15	27.354	35.833	East	35	49	0.078	26	Female
15	36.732	35.83522	East	35	50	6.792	44	Female
12	25	35.83115	East	35	49	52	30	Female
12	29	35.83323	East	35	49	59	42	Female
12	11	35.84088	East	35	50	27	65	Female
24	5.306	37.25006	East	37	15	0.222	27	Male
55	22.60128	35.1225	East	35	7	20.0964	49	Female
14	56.142	35.82892	East	35	49	44.13	34	Female
15	3.984	35.82969	East	35	49	46.89	48	Female
		34.91876					26	Male
0	41.478	34.92049	East	34	55	13.554	38	Female
49	29.68428	38.18989	East	38	11	23.6202	40	Male
15	32.982	35.83355	East	35	50	0.78	50	Male

		36.09651					51	Female
14	53.844	35.82194		35	49	47.631	51	Male
14	58.266	35.82871	East	35	49	43.368	71	Male
12	18	35.83381	East	35	50	1	42	Female
12	12	35.83194	East	35	49	54	30	Female
12	26	35.83162	East	35	49	53	58	Male
0	14.28368	34.92206	East	34	55	19.23914	34	Female
0	57.762	34.324	East	34	55	55.356	20	Male
14	58.536	35.8287		35	49	43.308	54	Male
		35.83273					65	Male
15	44.124	35.8339	East	35	50	2.058	37	Male
59	55.35384	35.11304	East	35	6	46.93968	56	Female
14	58.92	35.82863		35	49	43.068	24	Male
15	38.814	35.83478	East	35	50	5.19	26	Female
0	21.744		East	34	55	13.536	46	Female
0	16.41296	34.92173	East	34	55	18.22449	43	Female
0	40.818	34.91728	East	34	55	2.214	24	Female
0	43.464	34.91956	East	34	55	10.548	34	Female
24	22.77	37.25197	East	37	15	7.026	32	Male
		37.5697					22	Male
		36.19651					62	Male
							26	Female
14	56.586	35.82886	East	35	49	43.878	21	Female
15	27.466	35.83326	East	35	49	39.724	43	Female
15	7.728	35.83192	East	35	49	54.924	53	Male
0	7.65599	34.92346	East	34	55	24.45723	30	Female
0	16.43712	34.92188	East	34	55	18.75944	54	Female
23	28.9	37.2526	East	37	15	8.58	42	Female
0	30.636	34.92255	East	34	55	21.18	56	Male
55	54.3742	35.13843	East	35	8	18.33072	35	Male
55	27.14736	35.11639	East	35	6	58.99752	31	Male
29	7.56	37.26015	East	37	15	36.546	40	Female
23	31.524	37.25199	East	37	15	7.17	55	Male
		37.58128					42	Female
15	0.678	35.83002	East	35	49	47.06	28	Male
15	8.106	35.83493	East	35	50	6.468	43	Male
0	17.66829	34.9224	East	34	55	20.65324	38	Male
15	16.722	35.83194	East	35	50	13.302	94	Male
15	15.252	35.83505	East	35	50	6.174	19	Male
15	34.86	35.83176	East	35	49	59.604	20	Female
		35.08651					49	Male
15	0.918	35.82992		33	49	47.718	30	Male
14	50.76	35.82946		35	49	46.056	38	Female
15	1.014	35.82842	East	35	49	42.33	38	Male
		35.77465					54	Female
15	42.49	35.83372	East	35	50	1.386	40	Female
12	10	35.83155	East	35	49	53	61	Female

12	33	35.84195	East	35	50	31	53	Male
0	7.08	34.92284	East	34	55	25.812	50	Female
		34.91017					24	Female
		42.65008					54	Female
15	0.87	35.83001		35	49	48.024	29	Female
12	28	35.83243	East	35	49	56	35	Female
0	24.03	34.91998	East	34	55	11.94	40	Female
		36.08651					63	Female
14	53.532	35.82922		35	49	45.204	43	Female
		34.91713					32	Male
		34.91031					42	Male
15	14.65	35.83422	East	35	50	1.824	29	Female
23	36.792	37.25166	East	37	15	5.616	22	Female
15	39.06	35.83479	East	35	50	5.238	40	Female
46	53.77332	35.27685	East	35	16	36.64688	23	Male
		37.58728					45	Female
23	31.19	37.25193	East	37	15	6.99	35	Male
48	14.886	38.16535	East	38	9	55.248	37	Male
45	33.24	37.61475	East	37	36	53.094	42	Female
45	33.24	37.6138	East		37.36	49.674	29	Female
							63	Male
15	39.48	35.8336	East	35	50	0.9818	30	Female
15	45.48	35.83416	East	35	50	50.297	23	Female
12	19	35.83369	East	35	50	1	30	Male
15	5.04			35	49	49.194	46	Female
14	51.402	35.82941		35	49	45.876	42	Female
14	52.518	35.82928		35	49	45.426	36	Male
14	53.91	35.82904		35	49	44.898	52	Male
14	59.376	35.8286	East	35	49	42.978	37	Female
0	34.93307	54.45614	East	34	55	50.61	30	Male
0	28.326	34.91934	East	34	55	9.606	34	Male
0	32.826	34.92154	East	34	55	17.382	30	Female
0	34.206	34.92234	East	34	55	20.43	22	Female

education	occupation	others	produce	land	cultivated	consumpti	own_produ	maize_pur
Tertiary	Employed	N/A	Yes	5	3	Over 15 Gc	All	None
Tertiary	Full time fa	N/A	Yes	5	3	Over 15 Gc	All	None
Primary	Full time fa	N/A	Yes	0.25	0.25	Over 15 Gc	None	All
Tertiary	Employed	N/A	Yes	1.1	0.7	Over 15 Gc	All	None
Primary	Full time fa	N/A	Yes	3	2	Over 15 Gc	All	None
Primary	Full time fa	N/A	Yes	1	0.8	Over 15 Gc	Half	Half
Secondary	Employed	N/A	Yes	2	2	Over 15 Gc	All	None
Secondary	Business m	N/A	Yes	1	1	Over 15 Gc	All	None
Tertiary	Employed	N/A	Yes	4	3	Over 15 Gc	All	None
Primary	Full time fa	N/A	Yes	2	1.5	Over 15 Gc	All	None
Secondary	Full time fa	N/A	Yes	2	2	Over 15 Gc	Half	Half
Primary	Full time fa	N/A	Yes	3	2	Over 15 Gc	All	None
Primary	Full time fa	N/A	Yes	1	0.5	Over 15 Gc	All	None
Primary	Full time fa	N/A	Yes	3	1	Over 15 Gc	All	None
Secondary	Business m	N/A	Yes	1	1	11-15 Gorc	All	None
None	Full time fa	N/A	Yes	3	2	Over 15 Gc	Half	Half
Primary	Full time fa	N/A	Yes	1	1	Over 15 Gc	All	None
Primary	Business m	N/A	Yes	2	0.5	11-15 Gorc	All	None
Primary	Full time fa	N/A	Yes	2	1	Over 15 Gc	Half	Half
Primary	Full time fa	N/A	Yes	1	0.7	Over 15 Gc	All	None
None	Full time fa	N/A	Yes	2	2	Over 15 Gc	All	None
None	Full time fa	N/A	Yes	3	3	Over 15 Gc	All	None
Primary	Full time fa	N/A	Yes	2	1.5	Over 15 Gc	All	None
Secondary	Employed	N/A	Yes	0.5	0.5	11-15 Gorc	All	None
Secondary	Employed	N/A	Yes	2	2	11-15 Gorc	All	None
Primary	Full time fa	N/A	Yes	1	1	Over 15 Gc	All	None
Secondary	Full time fa	N/A	Yes	0.5	1	Over 15 Gc	Half	Half
Primary	Full time fa	N/A	Yes	0.5	0.5	Over 15 Gc	All	None
Primary	Full time fa	N/A	Yes	0.25	0	6-10 Gorog	None	All
Primary	Full time fa	N/A	Yes	1.5	0.5	11-15 Gorc	Half	None
Primary	Full time fa	N/A	Yes	0.75	0.25	11-15 Gorc	Half	Half
Primary	Full time fa	N/A	Yes	0.5	0.5	Over 15 Gc	Half	Half
Primary	Full time fa	N/A	Yes	0.5	0.5	Over 15 Gc	Half	Half
Secondary	Full time fa	N/A	Yes	1	0.5	Over 15 Gc	None	All
Secondary	Full time fa	N/A	Yes	1	0.25	11-15 Gorc	None	All
Tertiary	Full time fa	N/A	Yes	2	1	Over 15 Gc	All	None
Primary	Full time fa	N/A	Yes	1.5	1	Over 15 Gc	All	None
Primary	Full time fa	N/A	Yes	4	3	Over 15 Gc	All	None
None	Full time fa	N/A	Yes	3	2	Over 15 Gc	All	None
Secondary	Business m	N/A	Yes	5	3	Over 15 Gc	Half	Half
Primary	Full time fa	N/A	Yes	3	2	11-15 Gorc	All	None
Secondary	Employed	N/A	Yes	6	4	Over 15 Gc	All	None
Primary	Business m	N/A	Yes	0.5	0.5	11-15 Gorc	All	None
Tertiary	Employed	N/A	Yes	5	3	Over 15 Gc	All	None
Primary	Full time fa	N/A	Yes	4	3	Over 15 Gc	Half	Half
Secondary	Employed	N/A	Yes	3	2	Over 15 Gc	All	None

Primary	Full time fa	N/A	Yes	1.5	1	Over 15 Gc	All	None
Primary	Full time fa	N/A	Yes	1	0.8	11-15 Gorc	All	None
Primary	Full time fa	N/A	Yes	4	2	Over 15 Gc	All	None
Tertiary	Full time farmer		Yes	1	0.5	11-15 Gorc	Half	Half
Primary	Full time fa	N/A	Yes	1	0.5	11-15 Gorc	All	None
Primary	Full time fa	N/A	Yes	6	4	Over 15 Gc	All	None
Secondary	Business m	N/A	Yes	2	1	11-15 Gorc	All	None
Secondary	Employed	N/A	Yes	1	1	11-15 Gorc	All	None
Tertiary	Full time fa	Self employ	Yes	0	0	11-15 Gorc	None	All
Secondary	Full time fa	N/A	Yes	3	3	Over 15 Gc	All	None
Primary	Full time fa	N/A	Yes	0.5	0.25	Over 15 Gc	Half	Half
Tertiary	Employed	N/A	Yes	1	1	6-10 Gorog	All	None
Primary	Full time fa	N/A	Yes	1	0.5	Over 15 Gc	Half	Half
Primary	Full time fa	N/A	Yes	1	0.75	6-10 Gorog	All	None
Secondary	Full time fa	N/A	Yes	1	0.75	6-10 Gorog	All	None
Secondary	Business m	N/A	Yes	1.5	1	6-10 Gorog	All	None
Secondary	Full time fa	N/A	Yes	0.25	0.25	Over 15 Gc	Half	Half
Primary	Full time fa	N/A	Yes	0.25	0.25	Over 15 Gc	Half	Half
Primary	Full time fa	N/A	Yes	0.25	0.25	Over 15 Gc	Half	Half
Primary	Full time fa	N/A	Yes	0.13	0.13	Over 15 Gc	None	All
None	Full time fa	N/A	Yes	0.5	0	Over 15 Gc	None	All
Tertiary	Full time fa	N/A	Yes	1.5	1.5	Over 15 Gc	Half	Half
Tertiary	Full time fa	N/A	Yes	2	1.25	Over 15 Gc	All	None
Primary	Full time fa	N/A	Yes	2.5	1.75	Over 15 Gc	All	None
Tertiary	Employed	N/A	Yes	3	3	Over 15 Gc	All	All
Tertiary	Full time fa	N/A	Yes	2	1.5	Over 15 Gc	All	None
Tertiary	Employed	N/A	Yes	2.5	2	Over 15 Gc	All	None
Secondary	Full time fa	N/A	Yes	2	1.5	6-10 Gorog	All	None
Tertiary	Full time fa	N/A	Yes	3	2	Over 15 Gc	All	None
Tertiary	Employed	N/A	Yes	4	3.5	Over 15 Gc	All	All
Tertiary	Full time farmer		Yes	3	2.5	Over 15 Gc	All	All
Primary	Full time fa	N/A	Yes	1.5	0.75	Over 15 Gc	All	None
Primary	Full time fa	N/A	Yes	1	0.5	6-10 Gorog	All	None
Tertiary	Other		Yes	1.5	0.75	Over 15 Gc	All	None
Secondary	Employed	N/A	Yes	1	0.5	Over 15 Gc	All	None
Primary	Full time fa	N/A	Yes	0.75	0.5	Over 15 Gc	All	None
Primary	Full time fa	N/A	Yes	2	1	Over 15 Gc	All	None
Secondary	Employed	N/A	Yes	1.5	1	Over 15 Gc	All	None
None	Full time fa	N/A	Yes	2	1	Over 15 Gc	Half	Half
None	Full time fa	N/A	Yes	6	5	Over 15 Gc	None	All
None	Full time fa	N/A	Yes	2	2	Over 15 Gc	None	All
Primary	Full time fa	N/A	Yes	2	1	Over 15 Gc	None	All
Tertiary	Full time fa	N/A	Yes	1.5	1	Over 15 Gc	Half	Half
None	Full time fa	N/A	Yes	1	0.25	6-10 Gorog	None	All
Primary	Full time fa	N/A	Yes	1	0.25	6-10 Gorog	None	All
Primary	Full time fa	N/A	Yes	0.5	0.5	Over 15 Gc	None	All
Secondary	Full time fa	N/A	Yes	0.25	0.25	Over 15 Gc	All	None

None	Full time fa	N/A	Yes	0.75	0.75	Over 15 Gc	None	All
Primary	Full time fa	N/A	Yes	4	4	Over 15 Gc	All	None
Primary	Full time fa	N/A	Yes	1	0.5	1-5 Gorogc	All	None
Secondary	Full time fa	N/A	Yes	1	0.5	6-10 Gorog	None	All
Secondary	Full time fa	N/A	Yes	0.5	0.5	6-10 Gorog	All	None
Tertiary	Business m	N/A	Yes	1	0.5	Over 15 Gc	Half	Half
Primary	Full time fa	N/A	Yes	0.5	0.5	Over 15 Gc	None	All
Primary	Full time fa	N/A	Yes	1	0.5	Over 15 Gc	None	All
Primary	Full time fa	N/A	Yes	8	3	Over 15 Gc	None	All
Primary	Full time fa	N/A	Yes	6	5	Over 15 Gc	None	All
None	Full time fa	N/A	Yes	5	2	Over 15 Gc	None	All
Tertiary	Business m	N/A	Yes	4	2	Over 15 Gc	None	All
Primary	Full time fa	N/A	Yes	3	1	Over 15 Gc	None	All
Primary	Full time fa	N/A	Yes	3	1	Over 15 Gc	Half	Half
None	Full time fa	N/A	Yes	4	2	Over 15 Gc	All	None
None	Full time fa	N/A	Yes	11	5	Over 15 Gc	All	None
Secondary	Full time fa	N/A	Yes	4	2	11-15 Gorc	Half	Half
Secondary	Full time fa	N/A	Yes	1	1	Over 15 Gc	Half	Half
None	Full time fa	N/A	Yes	1	1	Over 15 Gc	None	All
Tertiary	Employed	N/A	Yes	15	10	Over 15 Gc	Half	Half
None	Full time fa	N/A	Yes	7	6	Over 15 Gc	All	None
Secondary	Full time fa	N/A	Yes	3	2	Over 15 Gc	Half	Half
None	Full time fa	N/A	Yes	4	3	Over 15 Gc	None	All
Primary	Full time fa	N/A	Yes	0.5	0.25	Over 15 Gc	Half	Half
Primary	Other		Yes	1.5	1.5	Over 15 Gc	None	All
Secondary	Full time fa	N/A	Yes	4	4	11-15 Gorc	All	None
Primary	Full time fa	N/A	Yes	0.25	0.25	1-5 Gorogc	Half	Half
Secondary	Full time fa	N/A	Yes	2	1	1-5 Gorogc	None	All
Primary	Full time fa	N/A	Yes	2	0.5	1-5 Gorogc	None	All
Secondary	Full time fa	N/A	Yes	3	2	Over 15 Gc	All	None
Primary	Full time fa	N/A	Yes	1	0.25	6-10 Gorog	None	All
Primary	Full time fa	N/A	Yes	1	0.25	1-5 Gorogc	None	All
Secondary	Full time fa	N/A	Yes	2.5	2	1-5 Gorogc	All	None
Secondary	Full time fa	N/A	Yes	0.25	0.13	6-10 Gorog	Half	Half
Secondary	Full time fa	N/A	Yes	2	1.5	Over 15 Gc	Half	Half
Primary	Full time fa	N/A	Yes	0.25	0.13	11-15 Gorc	Half	Half
Secondary	Full time fa	N/A	Yes	0.25	0.13	11-15 Gorc	Half	Half
Primary	Employed	N/A	Yes	0.25	0.25	6-10 Gorog	None	All
Primary	Full time fa	N/A	Yes	0.25	0.25	6-10 Gorog	None	All
Secondary	Full time fa	N/A	Yes	5	5	Over 15 Gc	All	None
Secondary	Full time fa	N/A	Yes	1	1	Over 15 Gc	All	None
Secondary	Full time fa	N/A	Yes	1.5	1.5	6-10 Gorog	All	None
Secondary	Full time fa	N/A	Yes	3	3	6-10 Gorog	All	None
Primary	Full time fa	N/A	Yes	0.5	0.5	1-5 Gorogc	All	None
Secondary	Full time fa	N/A	Yes	0.5	0.5	Over 15 Gc	None	All
None	Full time fa	N/A	Yes	1	1	Over 15 Gc	All	None
Tertiary	Full time fa	N/A	Yes	0.5	0.5	Over 15 Gc	All	None

Primary	Full time fa	N/A	Yes	1	0.5	Over 15 Gc	Half	Half
Secondary	Full time fa	N/A	Yes	1	0.5	11-15 Gorc	Half	Half
Secondary	Full time fa	N/A	Yes	1	0.5	Over 15 Gc	Half	Half
Primary	Full time fa	N/A	Yes	2	1	Over 15 Gc	Half	Half
Primary	Full time fa	N/A	Yes	0.5	0.25	11-15 Gorc	Half	Half
Primary	Full time fa	N/A	Yes	0.25	0.25	Over 15 Gc	Half	Half
Primary	Full time fa	N/A	Yes	0.25	0.13	11-15 Gorc	Half	Half
Secondary	Full time fa	N/A	Yes	4	2	Over 15 Gc	All	None
Primary	Full time fa	N/A	Yes	1	0.5	Over 15 Gc	None	All
Tertiary	Full time fa	N/A	Yes	7	3	Over 15 Gc	All	None
Tertiary	Employed	N/A	Yes	1	0.5	11-15 Gorc	All	None
Secondary	Full time fa	N/A	Yes	3	0.25	6-10 Gorog	All	None
Primary	Full time fa	N/A	Yes	4	2	11-15 Gorc	None	All
Primary	Full time fa	N/A	Yes	1	1	Over 15 Gc	All	None
Secondary	Full time fa	N/A	Yes	2	2	11-15 Gorc	All	None
Secondary	Full time fa	N/A	Yes	0.25	0.25	Over 15 Gc	All	None
Secondary	Full time fa	N/A	Yes	1	0.75	Over 15 Gc	All	None
None	Full time fa	N/A	Yes	2	1	Over 15 Gc	None	All
Primary	Full time fa	N/A	Yes	3	2	Over 15 Gc	None	All
Primary	Full time fa	N/A	Yes	6	5	Over 15 Gc	None	All
Primary	Full time fa	N/A	Yes	6	5	Over 15 Gc	None	All
Primary	Full time fa	N/A	Yes	6	5	Over 15 Gc	None	All
Primary	Full time fa	N/A	Yes	1	0.5	Over 15 Gc	None	All
Primary	Full time fa	N/A	Yes	6	5	Over 15 Gc	None	All
Primary	Employed	N/A	Yes	5	0.5	Over 15 Gc	None	All
None	Full time fa	N/A	Yes	3	5	Over 15 Gc	None	All
Primary	Full time fa	N/A	Yes	6	5	Over 15 Gc	None	All
Primary	Full time fa	N/A	Yes	5	2	11-15 Gorc	None	All
Secondary	Business m	N/A	Yes	12	7	Over 15 Gc	Half	Half
Primary	Full time fa	N/A	Yes	8	3	Over 15 Gc	None	All
Primary	Full time fa	N/A	Yes	5	4	Over 15 Gc	None	All
Secondary	Employed	N/A	Yes	3	1	6-10 Gorog	None	All
None	Full time fa	N/A	Yes	4	2	Over 15 Gc	None	All
Secondary	Full time fa	N/A	Yes	2	1	Over 15 Gc	Half	Half
Secondary	Full time fa	N/A	Yes	2	1	Over 15 Gc	Half	Half
Primary	Full time fa	N/A	Yes	3	2.5	Over 15 Gc	None	All
Primary	Other	Artisan	Yes	1	0.5	Over 15 Gc	Half	Half
Tertiary	Employed	N/A	Yes	5	5	Over 15 Gc	Half	Half
Secondary	Employed	N/A	Yes	7	3	Over 15 Gc	Half	Half
Secondary	Full time fa	N/A	Yes	10	8	Over 15 Gc	Half	Half
None	Full time fa	N/A	Yes	4	2	Over 15 Gc	None	All
Primary	Full time fa	N/A	Yes	1.5	0.5	Over 15 Gc	All	None
Tertiary	Employed	N/A	Yes	1.5	1.5	6-10 Gorog	All	None
Secondary	Business m	N/A	Yes	1.5	1.5	1-5 Gorogc	All	All
Primary	Full time fa	N/A	Yes	10	10	Over 15 Gc	All	None
Secondary	Business m	N/A	Yes	2	2	1-5 Gorogc	None	All
Tertiary	Business m	N/A	Yes	2	1	Over 15 Gc	All	None

Primary	Full time fa	N/A	Yes	1.5	1.5	11-15 Gorc	None	All
Primary	Full time fa	N/A	Yes	3	3	Over 15 Gc	Half	Half
None	Full time fa	N/A	Yes	2	2	11-15 Gorc	Half	Half
Tertiary	Employed	N/A	Yes	2.5	2.5	1-5 Gorogc	All	None
Primary	Full time fa	N/A	Yes	1.5	0	Over 15 Gc	None	All
Primary	Employed	N/A	Yes	2.5	2.5	11-15 Gorc	None	All
None	Full time fa	N/A	Yes	2	2	11-15 Gorc	Half	Half
Primary	Other	Dry cleanei	Yes	1	1	6-10 Gorog	All	None
Secondary	Other	Casual labc	Yes	0.5	0.5	1-5 Gorogc	All	None
Secondary	Other	Casual labc	Yes	0.5	0.5	Over 15 Gc	Half	Half
Tertiary	Employed	N/A	Yes	4	4	6-10 Gorog	All	None
Tertiary	Employed	N/A	Yes	2	2	1-5 Gorogc	All	None
Tertiary	Employed	N/A	Yes	1.5	1.5	6-10 Gorog	All	None
Primary	Business m	N/A	Yes	1	1	Over 15 Gc	All	None
Tertiary	Employed	N/A	Yes	2	2	6-10 Gorog	None	All
Secondary	Full time fa	N/A	Yes	2	1	1-5 Gorogc	Half	Half
Primary	Full time fa	N/A	Yes	2	1	11-15 Gorc	Half	Half
Secondary	Full time fa	N/A	Yes	2	0.5	11-15 Gorc	All	None
Secondary	Full time fa	N/A	Yes	2	1	6-10 Gorog	All	None
Primary	Full time fa	N/A	Yes	2	0.25	6-10 Gorog	All	None
Primary	Full time fa	N/A	Yes	4	3	Over 15 Gc	All	None
Secondary	Full time fa	N/A	Yes	3	2	Over 15 Gc	All	None
Primary	Other	Dependant	Yes	1	0.5	1-5 Gorogc	Half	Half
Secondary	Full time fa	N/A	Yes	12	0.5	Over 15 Gc	All	None
Primary	Full time fa	N/A	Yes	3	2	6-10 Gorog	All	None
Secondary	Full time fa	N/A	Yes	3	3	6-10 Gorog	Half	None
Primary	Full time fa	N/A	Yes	1	1	6-10 Gorog	All	None
None	Full time fa	N/A	Yes	2	2	6-10 Gorog	Half	None
Secondary	Business m	N/A	Yes	3	1	Over 15 Gc	All	None
Secondary	Business m	N/A	Yes	8	8	Over 15 Gc	All	None
Primary	Full time fa	N/A	Yes	4	4	Over 15 Gc	All	None
Secondary	Full time fa	N/A	Yes	7	5	Over 15 Gc	All	None
Secondary	Full time fa	N/A	Yes	5	5	Over 15 Gc	All	None
Tertiary	Full time fa	N/A	Yes	3.5	3.5	Over 15 Gc	All	None
Secondary	Full time fa	N/A	Yes	1	1	6-10 Gorog	All	None
Primary	Full time fa	N/A	Yes	1	1	Over 15 Gc	All	None
Secondary	Full time fa	N/A	Yes	2	2	6-10 Gorog	All	None
Primary	Full time fa	N/A	Yes	3	2.5	11-15 Gorc	All	None
Primary	Full time fa	N/A	Yes	5	3	Over 15 Gc	All	None
Tertiary	Full time fa	N/A	Yes	30	15	Over 15 Gc	All	None
None	Full time fa	N/A	Yes	8	4	Over 15 Gc	Half	Half
Primary	Full time fa	N/A	Yes	2	1	11-15 Gorc	All	None
Secondary	Full time fa	N/A	Yes	2	2	11-15 Gorc	All	None
None	Employed	N/A	Yes	1.5	7	Over 15 Gc	All	None
Primary	Full time fa	N/A	Yes	1.25	0.75	Over 15 Gc	All	None
None	Business m	N/A	Yes	4	1	Over 15 Gc	All	None
Primary	Full time fa	N/A	Yes	2	1.5	Over 15 Gc	All	None

Primary	Full time fa	N/A	Yes	8	5	6-10 Gorog	All	None
Secondary	Employed	N/A	Yes	8	5	11-15 Gorc	All	None
Secondary	Full time fa	N/A	Yes	6.5	5	Over 15 Gc	All	None
Tertiary	Employed	N/A	Yes	4	4	Over 15 Gc	All	None
Secondary	Full time farmer		Yes	2	1.5	6-10 Gorog	All	None
Tertiary	Full time fa	N/A	Yes	4	3.25	6-10 Gorog	All	None
Secondary	Full time fa	N/A	Yes	2	1.5	Over 15 Gc	All	None
Secondary	Full time fa	N/A	Yes	1.5	1.5	Over 15 Gc	All	None
Primary	Full time fa	N/A	Yes	4.5	3.5	Over 15 Gc	All	None
Secondary	Employed	N/A	Yes	2	2	Over 15 Gc	All	None
Primary	Full time fa	N/A	Yes	0.75	0.75	11-15 Gorc	Half	Half
None	Full time fa	N/A	Yes	6	4	Over 15 Gc	All	None
Tertiary	Full time fa	N/A	Yes	2.75	2	11-15 Gorc	All	None
Primary	Full time fa	N/A	Yes	1	1	Over 15 Gc	All	None
Primary	Full time fa	N/A	Yes	1	1	Over 15 Gc	Half	Half
Tertiary	Employed	N/A	Yes	1.75	0.5	Over 15 Gc	All	None
Tertiary	Employed	N/A	Yes	2	0.5	Over 15 Gc	Half	Half
None	Full time fa	N/A	Yes	1.5	0.5	Over 15 Gc	All	None
Primary	Full time fa	N/A	Yes	1	0.25	6-10 Gorog	All	None
Secondary	Full time fa	N/A	Yes	2	2	6-10 Gorog	Half	Half
Primary	Full time fa	N/A	Yes	10	8	6-10 Gorog	All	None
Tertiary	Employed	N/A	Yes	3	2	Over 15 Gc	All	None
Secondary	Business m	N/A	Yes	1	1	6-10 Gorog	All	None
Primary	Full time fa	N/A	Yes	1.5	1	11-15 Gorc	All	None
Primary	Full time fa	N/A	Yes	3	3	Over 15 Gc	All	None
Primary	Full time fa	N/A	Yes	6	5	Over 15 Gc	None	All
Secondary	Full time fa	N/A	Yes	3	2.5	Over 15 Gc	All	None
Secondary	Full time fa	N/A	Yes	1	0.5	11-15 Gorc	Half	Half
None	Full time fa	N/A	Yes	2	2	Over 15 Gc	Half	Half
Primary	Business m	N/A	Yes	5	4	Over 15 Gc	All	None
Tertiary	Employed	N/A	Yes	4	4	11-15 Gorc	All	None
Primary	Full time fa	N/A	Yes	1	0.5	Over 15 Gc	Half	Half
Primary	Full time fa	N/A	Yes	2	0.5	6-10 Gorog	Half	Half
Primary	Full time fa	N/A	Yes	3	2	1-5 Gorogc	Half	Half
Secondary	Full time fa	N/A	Yes	2.5	2	11-15 Gorc	All	None
Secondary	Full time fa	N/A	Yes	5	2.5	11-15 Gorc	All	None
Secondary	Full time fa	N/A	Yes	5	1	Over 15 Gc	None	All
None	Employed	N/A	Yes	0.5	0.5	11-15 Gorc	All	None
Secondary	Full time fa	N/A	Yes	3	2	Over 15 Gc	All	None
Secondary	Full time fa	N/A	Yes	1	1	6-10 Gorog	All	None
Primary	Full time fa	N/A	Yes	3	1	11-15 Gorc	All	None
Secondary	Full time fa	N/A	Yes	2	2	Over 15 Gc	All	None
Secondary	Full time fa	N/A	Yes	2	2	Over 15 Gc	All	None
Secondary	Full time fa	N/A	Yes	3	1.73	11-15 Gorc	All	None
Primary	Full time fa	N/A	Yes	1.25	1	11-15 Gorc	All	None
Primary	Full time fa	N/A	Yes	5	4	11-15 Gorc	All	None
Primary	Full time fa	N/A	Yes	3	0.5	Over 15 Gc	All	None

Tertiary	Employed	N/A	Yes	2	1.5	11-15 Gorc	All	None
None	Full time fa	N/A	Yes	1	1	Over 15 Gc	None	All
Tertiary	Full time fa	N/A	Yes	10	4	6-10 Gorog	All	None
Secondary	Full time fa	N/A	Yes	12	1	Over 15 Gc	All	All
Primary	Full time fa	N/A	Yes	3.5	3.5	Over 15 Gc	All	None
Tertiary	Business m	N/A	Yes	2	1	11-15 Gorc	All	None
Secondary	Full time fa	N/A	Yes	1	1	Over 15 Gc	Half	Half
Secondary	Full time fa	N/A	Yes	12	7	6-10 Gorog	All	None
None	Business m	N/A	Yes	3	3	11-15 Gorc	All	None
Tertiary	Full time fa	N/A	Yes	2	1.5	6-10 Gorog	Half	Half
Secondary	Full time fa	N/A	Yes	2	1.5	6-10 Gorog	Half	Half
Primary	Full time fa	N/A	Yes	1.5	1	Over 15 Gc	All	None
Secondary	Full time fa	N/A	Yes	1	0.5	Over 15 Gc	Half	Half
Primary	Full time fa	N/A	Yes	0.5	0.5	Over 15 Gc	Half	Half
Tertiary	Full time fa	N/A	Yes	1	1	1-5 Gorogc	None	All
Primary	Full time fa	N/A	Yes	4	3.5	Over 15 Gc	All	None
Secondary	Full time fa	N/A	Yes	2	1.5	Over 15 Gc	Half	Half
None	Employed	N/A	Yes	4	4	Over 15 Gc	All	None
Secondary	Employed	N/A	Yes	1	1	1-5 Gorogc	Half	Half
Primary	Full time fa	N/A	Yes	1	1	6-10 Gorog	Half	Half
Primary	Full time fa	N/A	Yes	2.5	2	11-15 Gorc	Half	Half
Primary	Full time fa	N/A	Yes	1	0.5	Over 15 Gc	None	All
Primary	Full time fa	N/A	Yes	2.5	2	Over 15 Gc	All	None
Secondary	Full time fa	N/A	Yes	2	1.5	6-10 Gorog	All	None
Primary	Full time fa	N/A	Yes	2	2	Over 15 Gc	All	None
Tertiary	Full time fa	N/A	Yes	5	5	Over 15 Gc	All	None
Secondary	Business m	N/A	Yes	4	4	11-15 Gorc	All	None
Primary	Full time fa	N/A	Yes	5	4	Over 15 Gc	All	None
Secondary	Full time fa	N/A	Yes	3	2	11-15 Gorc	All	None
Tertiary	Employed	N/A	Yes	0.5	0.5	Over 15 Gc	All	None
Secondary	Business m	N/A	Yes	1	1	Over 15 Gc	Half	Half
Primary	Full time fa	N/A	Yes	2.5	2	Over 15 Gc	All	None
Secondary	Full time fa	N/A	Yes	8	7	Over 15 Gc	All	None

human_mc	animal_mo	milk_cowfe	mix_dry_w	goog_wet_	sell_mold_	home_cons	quality_gra	discolourec
No	Yes	Yes	No	No	No	30	Good	2
No	No	No	No	No	No	40	Good	0
No	No	Yes	No	No	No	0		
No	Yes	Yes	No	No	Yes	30	Good	0
No	Yes	Yes	No	No	Yes	30	Good	0
No	Yes	Yes	No	No	Yes	30	Good	0
No	No	No	No	No	Yes	30	Good	0
No	Yes	Yes	No	No	Yes	30	Good	0
No	No	No	No	No	No	30	Good	0
No	Yes	Yes	No	No	Yes	30	Good	0
No	Yes	Yes	No	No	Yes	30	Good	0
No	Yes	Yes	No	No	Yes	30	Good	0
No	Yes	Yes	No	No	Yes	30	Good	0
No	Yes	Yes	No	No	No	30	Good	0
No	No	No	No	No	Yes	30	Good	0
No	Yes	Yes	No	No	Yes	30	Good	0
No	Yes	Yes	No	No	Yes	30	Good	0
No	No	Yes	No	No	No	30	Good	0
No	Yes	Yes	No	No	Yes	30	Good	0
No	Yes	Yes	No	No	Yes	30	Good	0
No	No	No	No	No	No	450	Good	0
No	Yes	Yes	No	No	Yes	30	Good	0
No	Yes	Yes	No	No	Yes	30	Good	0
No	No	No	No	No	No	30	Good	0
No	No	No	No	No	No	540	Good	0
No	Yes	Yes	No	No	Yes	30	Good	0
No	No	Yes	No	No	No	90	Good	90
No	No	No	No	No	No	56	Good	0
No	No	No	No	No	No	0		
No	No	Yes	No	No	No	20	Good	0
No	No	No	No	No	No	24	Good	0
No	No	No	No	No	No	0		
No	No	No	No	No	No	0		
No	Yes	Yes	No	No	No	0		
No	Yes	Yes	No	No	Yes	0		
No	Yes	Yes	No	No	No	30	Good	4
No	Yes	Yes	No	No	No	40	Good	0
No	No	No	No	No	Yes	30	Good	0
No	No	No	No	No	No	30	Good	0
No	Yes	Yes	No	No	Yes	30	Good	0
No	Yes	Yes	No	No	Yes	30	Good	0
No	No	No	No	No	No	30	Good	0
No	No	No	No	No	No	30	Good	0
No	No	No	No	No	No	30	Good	0
No	Yes	Yes	No	No	Yes	30	Good	0
No	No	No	No	No	No	30	Good	0

No	Yes	Yes	No	No	Yes	30	Good	0
No	No	Yes	No	No	No	30	Good	0
No	Yes	Yes	No	No	Yes	30	Good	0
No	No	Yes	No	No	No	10	Good	0
No	No	No	No	No	No	30	Good	0
No	Yes	Yes	No	No	Yes	30	Good	0
No	No	No	No	No	Yes	30	Good	0
No	No	No	No	No	No	360	Good	0
No	No	No	No	No	Yes	0		
No	No	No	No	No	No	30	Good	0
Yes	Yes	Yes	Yes	Yes	Yes	40	Good	0
No	No	No	No	No	No	16	Good	0
No	No	No	No	No	No	56	Good	0
No	No	No	No	No	No	20	Good	0
No	Yes	No	No	No	No	20	Good	0
No	Yes	No	No	No	No	16	Good	0
No	No	No	No	No	No	0		
No	No	No	No	No	No	0		
No	No	No	No	No	No	0		
No	No	No	No	No	No	0		
No	Yes	Yes	No	No	No	0		
No	No	No	No	No	No	90	Good	45
No	No	No	No	No	No	90	Good	0
No	Yes	Yes	No	No	Yes	90	Good	0
No	Yes	Yes	No	No	No	30	Good	4
No	Yes	Yes	No	No	Yes	30	Good	2
No	Yes	Yes	No	No	Yes	30	Good	6
No	Yes	Yes	No	No	Yes	20	Good	45
No	Yes	Yes	No	No	Yes	30	Good	2
No	Yes	Yes	No	No	No	30	Good	4
No	Yes	Yes	No	Yes	No	30	Good	4
No	No	No	No	No	Yes	60	Good	15
No	Yes	Yes	No	No	Yes	15	Good	0
No	Yes	Yes	No	No	No	50	Bad	0
No	No	Yes	No	No	No	40	Good	0
No	No	No	No	No	No	40	Good	0
No	No	Yes	No	No	No	40	Good	0
No	No	No	No	No	No	90	Good	0
No	No	No	No	No	No	0		
No	No	No	No	No	No	0		
No	No	No	No	No	No	0		
No	No	No	No	No	No	0		
No	No	No	No	No	No	5	Good	0
No	Yes	Yes	No	No	No	0		
No	Yes	Yes	No	No	No	0		
No	No	No	No	No	No	0		
No	No	No	No	No	No	90	Good	0

No	No	No	No	No	No	0		
No	No	No	No	No	No	0		
No	No	No	No	No	No	10	Good	2
No	No	No	No	No	No	15	Good	1
No	No	No	No	No	No	90	Good	0
Yes	Yes	Yes	Yes	No	No	60	Good	5
No	No	No	No	No	No	10	Bad	5
No	No	No	No	No	No	0		
No	No	No	No	No	No	0		
No	No	No	No	No	No	0		
No	No	No	No	No	No	0		
No	No	No	No	No	No	0		
No	No	No	No	No	No	0		
Yes	Yes	Yes	Yes	Yes	Yes	0		
No	No	No	No	No	No	0		
No	No	No	No	No	No	1080	Good	0
No	No	No	No	No	No	90	Good	10
No	No	No	No	No	No	45	Good	0
No	No	No	No	No	No	0		
No	No	No	No	No	No	45	Good	0
No	No	No	No	No	No	50	Good	0
No	No	No	No	No	No	30	Good	0
No	No	No	No	No	No	0		
No	No	Yes	Yes	No	No	90	Good	0
No	No	No	No	No	No	55	Good	0
No	No	No	No	No	No	20	Good	0
No	No	No	No	No	No	0		
Yes	Yes	Yes	No	No	No	0		
Yes	Yes	Yes	Yes	Yes	Yes	0		
No	No	Yes	No	No	No	100	Good	0
No	No	No	No	No	No	0		
No	No	No	No	No	No	10	Good	0
No	No	No	No	No	No	4	Good	2
No	No	No	No	No	No	180	Good	0
No	No	No	No	No	No	180	Good	0
No	No	No	No	No	No	23	Good	0
No	No	No	No	No	No	17	Good	0
No	No	No	No	No	No	0		
No	No	No	No	No	No	0		
No	No	No	No	No	No	40	Good	0
No	No	No	No	No	No	60	Good	4
No	No	No	No	No	No	20	Good	2
No	No	No	No	No	No	20	Good	0
No	No	No	No	No	No	90	Good	2
No	No	No	No	No	No	0		
No	No	No	No	No	No	90	Good	10
No	No	No	No	No	No	270	Good	0

No	No	No	No	No	No	20	Good	23
No	No	No	No	No	No	15	Good	2
No	No	No	No	No	No	14	Good	0
No	No	No	No	No	No	18	Good	10
No	No	No	No	No	No	1	Good	0
No	No	No	No	No	No	0		
No	No	No	No	No	No	23	Good	0
No	No	No	No	No	No	35	Good	0
Yes	Yes	Yes	No	No	No	0		
No	Yes	Yes	No	No	No	35	Good	0
No	No	No	No	No	No	15	Good	0
No	No	No	No	No	No	16	Good	0
No	No	No	No	No	No	30	Good	0
No	No	No	No	No	No	60	Good	5
No	No	No	No	No	No	450	Good	18
No	No	No	No	No	No	0		
Yes	Yes	Yes	Yes	Yes	Yes	45	Good	7
No	No	No	No	No	No	0		
No	No	No	No	No	No	0		
No	No	No	No	No	No	0		
No	No	No	No	No	No	0		
No	No	No	No	No	No	0		
No	No	No	No	No	No	0		
No	No	No	No	No	No	0		
No	No	No	No	No	No	0		
No	No	No	No	No	No	0		
No	No	No	No	No	No	0		
No	No	No	No	No	No	0		
No	No	No	No	No	No	40	Good	5
No	No	No	No	No	No	0		
No	No	No	No	No	No	0		
No	No	No	No	No	No	0		
No	No	No	Yes	No	No	0		
No	No	No	No	No	No	0		
No	No	No	No	No	No	0		
No	No	No	No	No	No	50	Good	0
Yes	No	Yes	No	No	No	20	Good	5
No	No	No	Yes	No	No	30	Good	0
No	No	No	No	No	No	180	Good	0
No	Yes	Yes	No	No	Yes	40	Good	0
No	No	No	No	No	No	0		
Yes	Yes	Yes	Yes	Yes	Yes	80	Good	0
No	No	No	No	No	No	20	Good	0
No	No	No	Yes	No	No	12	Good	0
No	No	No	No	No	No	36	Good	0
No	No	No	No	No	No	0		
No	No	Yes	Yes	No	No	0		

No	No	No	No	No	No	30	Good	0
No	No	No	No	No	No	40	Good	0
No	No	No	No	No	No	21	Good	0
No	No	No	No	No	No	10	Good	2
No	No	No	No	No	No	0		
No	No	No	No	No	No	24	Good	0
No	No	No	No	No	No	24	Good	0
No	No	No	No	No	No	12	Good	0
No	No	No	No	No	No	2	Good	0
No	No	No	No	No	No	0		
No	No	Yes	No	No	No	20	Good	0
No	No	No	No	No	No	10	Good	0
No	No	Yes	Yes	No	No	20	Good	0
No	No	No	No	No	No	90	Good	0
No	No	No	No	No	No	0		
No	No	Yes	No	No	No	50	Good	0
No	No	Yes	No	No	No	20	Good	0
No	Yes	Yes	No	No	No	25	Good	0
No	No	Yes	No	No	No	20	Good	0
No	No	No	No	No	No	18	Good	0
No	No	No	No	No	No	40	Good	0
No	No	Yes	No	No	No	90	Good	0
No	No	No	No	No	No	12	Good	1
No	No	No	No	No	No	60	Good	0
No	No	Yes	No	No	No	40	Good	0
No	No	No	No	No	No	10	Good	0
No	No	No	No	No	No	20	Good	0
No	No	No	No	No	No	30	Good	0
No	No	No	No	No	No	12	Good	2
No	No	No	No	No	No	45	Good	4
No	No	No	No	No	No	90	Good	23
No	No	No	No	No	No	30	Good	0
No	No	No	No	No	No	35	Good	
No	No	No	No	No	No	30	Good	0
No	Yes	No	No	No	No	16	Good	0
No	Yes	No	No	No	No	40	Good	0
No	No	No	No	No	No	16	Good	0
No	No	No	No	No	No	28	Good	0
No	No	No	No	No	No	40	Good	0
No	No	No	No	No	No	40	Good	0
No	Yes	Yes	No	No	Yes	30	Good	0
No	No	No	No	No	No	1350	Good	10
No	No	No	No	No	No	24	Bad	23
No	Yes	Yes	No	No	No	40	Good	0
No	Yes	Yes	No	No	Yes	30	Good	0
No	No	No	No	No	No	35	Good	0
No	No	No	No	No	No	80	Good	0

No	No	No	No	No	No	30	Good	0
No	No	No	No	No	No	30	Good	0
No	No	No	No	No	No	90	Good	23
No	No	No	No	No	No	64	Good	0
No	Yes	No	No	No	No	20	Good	0
No	No	No	No	No	No	20	Good	0
No	No	Yes	No	No	No	90	Good	2
No	No	No	No	No	No	56	Good	8
No	No	No	No	No	No	90	Good	68
No	No	No	No	No	No	44	Good	0
No	No	No	No	No	No	900	Good	0
No	Yes	Yes	No	No	Yes	30	Good	0
No	No	No	No	No	No	30	Good	0
No	Yes	No	No	No	No	40	Good	0
No	No	No	No	No	No	0		
No	No	No	No	No	No	90	Good	0
No	Yes	Yes	No	No	No	40	Good	4
No	No	No	No	No	No	90	Good	0
No	No	No	No	No	No	10	Good	0
No	No	No	No	No	No	90	Good	0
No	No	No	No	No	No	30	Good	0
No	No	No	No	No	No	30	Good	0
No	No	No	No	No	No	30	Good	0
No	No	No	No	No	No	24	Good	0
No	No	No	No	No	No	80	Good	0
Yes	Yes	Yes	No	Yes	Yes	0		
No	No	Yes	No	No	No	50	Good	0
No	No	No	No	No	No	7	Good	1
No	No	No	No	No	No	120	Good	0
No	Yes	Yes	No	No	No	30	Good	0
No	No	No	No	No	No	30	Good	0
No	No	No	No	No	No	45	Good	0
No	No	No	No	No	No	45	Good	0
No	No	No	No	No	No	90	Good	20
No	No	No	No	No	No	30	Good	23
No	No	No	No	No	No	24	Good	0
No	No	No	No	No	No	36	Good	0
No	No	No	No	No	No	30	Good	0
No	No	No	No	No	No	45	Good	0
No	No	No	No	No	No	90	Good	45
No	No	No	No	No	No	30	Good	0
No	No	No	No	No	No	90	Good	0
No	No	No	No	No	No	30	Good	0
No	No	No	No	No	No	30	Good	0
No	No	No	No	No	No	24	Good	0
No	No	No	No	No	No	20	Good	0
No	Yes	No	No	No	No	40	Good	0

No	No	No	No	No	No	24	Good	0
No	No	No	No	No	No	0		
No	Yes	Yes	No	No	No	20	Good	2
No	No	No	No	No	No	45	Good	0
No	No	No	No	No	No	30	Good	45
No	Yes	No	No	No	No	30	Good	0
No	No	No	No	No	No	0		
No	No	No	No	No	No	30	Good	0
No	No	No	No	No	No	30	Good	0
No	Yes	Yes	No	No	Yes	8	Good	45
No	Yes	Yes	No	No	Yes	8	Good	45
No	No	No	No	No	No	40	Good	0
No	No	No	No	No	No	12	Good	0
No	No	No	No	No	No	72	Good	0
No	No	No	No	No	No	30	Good	0
Yes	No	No	No	No	Yes	60	Good	0
No	No	No	No	No	No	54	Good	0
No	No	No	No	No	No	30	Good	0
No	No	Yes	No	No	No	10	Good	0
No	Yes	Yes	No	No	No	20	Good	0
No	Yes	Yes	No	No	No	100	Bad	45
No	No	No	No	No	No	20	Good	0
No	No	No	No	No	No	35	Good	0
No	Yes	No	No	No	No	20	Good	0
No	No	No	No	No	No	60	Good	45
No	No	No	No	No	No	30	Good	0
No	No	No	No	No	No	30	Good	0
No	No	No	No	No	No	30	Good	0
No	No	No	No	No	No	30	Good	0
No	No	No	No	No	No	45	Good	6
No	No	No	No	No	No	90	Good	0
No	Yes	Yes	No	No	No	30	Good	4
No	Yes	Yes	No	No	No	90	Good	45

do_with_d	chemical	insect	ash	airing	actel_dust	act_supper	malathion_	skana_supp
Fed Poultry	Yes	No	No	No	No	Yes	No	No
	Yes	No	No	No	Yes	No	No	No
	No	No	No	Yes	N/A	N/A	N/A	N/A
	No	No	No	Yes	N/A	N/A	N/A	N/A
	No	No	No	Yes	N/A	N/A	N/A	N/A
	No	No	No	Yes	N/A	N/A	N/A	N/A
	No	No	No	Yes	N/A	N/A	N/A	N/A
	No	No	No	Yes	N/A	N/A	N/A	N/A
	No	No	No	Yes	N/A	N/A	N/A	N/A
	No	No	No	Yes	N/A	N/A	N/A	N/A
	No	No	No	Yes	N/A	N/A	N/A	N/A
Fed cows	No	No	No	Yes	N/A	N/A	N/A	N/A
	No	No	No	Yes	N/A	N/A	N/A	N/A
	No	No	No	Yes	N/A	N/A	N/A	N/A
	No	No	No	Yes	N/A	N/A	N/A	N/A
	No	No	No	Yes	N/A	N/A	N/A	N/A
	No	No	No	Yes	N/A	N/A	N/A	N/A
	No	No	No	Yes	N/A	N/A	N/A	N/A
	No	No	No	Yes	N/A	N/A	N/A	N/A
	No	No	No	Yes	N/A	N/A	N/A	N/A
	No	No	No	Yes	N/A	N/A	N/A	N/A
Fed cows	No	No	No	Yes	N/A	N/A	N/A	N/A
	No	No	No	Yes	N/A	N/A	N/A	N/A
	No	No	No	Yes	N/A	N/A	N/A	N/A
	No	No	No	Yes	N/A	N/A	N/A	N/A
	No	No	No	Yes	N/A	N/A	N/A	N/A
Disposed/E	No	Yes	No	No	Yes	No	No	No
Fed cows	No	No	No	Yes	N/A	N/A	N/A	N/A
	No	No	No	Yes	N/A	N/A	N/A	N/A
Disposed/E	No	No	No	Yes	N/A	N/A	N/A	N/A
Disposed/E	No	No	No	Yes	N/A	N/A	N/A	N/A
	No	No	Yes	No	N/A	N/A	N/A	N/A
	No	No	No	No	No	N/A	No	No
Fed cows	No	No	No	Yes	N/A	No	N/A	N/A
Fed cows	No	No	No	Yes	No	No		No
Fed Poultry	No	No	No	No	N/A	No	N/A	N/A
Fed Poultry	No	No	No	Yes	N/A	No	N/A	N/A
	Yes	No	No	No	No	Yes	No	No
	Yes	No	No	No	No	Yes	No	No
	Yes	No	No	No	No	Yes	No	No
	Yes	No	No	No	No	Yes	No	No
	Yes	No	No	No	No	Yes	No	No
	Yes	No	No	No	No	Yes	No	No
	Yes	No	No	No	No	Yes	No	No
	Yes	No	No	No	No	Yes	No	No
	Yes	No	No	No	No	Yes	No	No

	Yes	No	No	No	No	Yes	No	No
	Yes	No	No	No	No	Yes	No	No
	Yes	No	No	No	No	Yes	No	No
Consume	Yes	No	No	No	No	No	Yes	No
	Yes	No	No	No	Yes	No	No	No
	Yes	No	No	No	No	Yes	No	No
	Yes	No	No	No	No	Yes	No	No
	Yes	No	No	No	No	Yes	No	No
	Yes	No	No	No	No	Yes	No	No
	Yes	No	No	Yes	No	Yes	No	No
Disposed/E	Yes	No	No	No	Yes	No	No	No
Disposed/E	Yes	No	No	No	No	No	No	Yes
Fed cows	Yes	No	No	No	No	Yes	No	No
Disposed/E	Yes	No	No	Yes	No	Yes	No	No
Fed cows	Yes	No	No	No	No	Yes	No	No
Fed cows	Yes	No	No	No	No	No	No	Yes
Fed cows	Yes	No	No	No	No	Yes	No	No
	Yes	No	No	No	No	Yes	No	No
Fed cows	Yes	No	No	No	Yes	No	No	No
	Yes	No	No	No	No	Yes	No	No
Fed cows	Yes	No	No	No	Yes	No	No	No
Fed cows	Yes	No	No	No	No	Yes	No	No
Fed cows	Yes	No	No	No	No	Yes	No	No
Fed cows	Yes	No	No	No	No	Yes	No	No
Disposed/E	Yes	No	No	No	No	Yes	No	No
Fed Poultry	Yes	No	No	No	No	Yes	No	No
Fed Poultry	Yes	No	No	No	No	Yes	No	No
Fed Poultry	Yes	No	No	No	No	Yes	No	No
Fed Poultry	Yes	No	No	No	No	Yes	No	No
Disposed/E	Yes	No	No	No	No	Yes	No	No
Disposed/E	Yes	No	No	No	No	Yes	No	No
Consume	Yes	No	No	No	Yes	No	No	No
Sold	Yes	No	No	No	Yes	No	No	No
Fed cows	Yes	No	No	No	Yes	No	No	No
Disposed/E	Yes	No	No	No	No	Yes	No	No
Fed Poultry	Yes	No	No	No	No	Yes	No	No
Used for Br	Yes	No	No	No	No	Yes	No	No
Fed cows	Yes	No	No	No	Yes	No	No	No
Disposed/E	No	No	No	No	No	No	No	No
Disposed/E	No	No	Yes	No	Yes	No	No	No
Consume	No	No	No	No	N/A	No	N/A	N/A
Disposed/E	Yes	No	No	Yes	Yes	No	No	No
	No	No	No	Yes	N/A	N/A	N/A	N/A
	No	No	No	Yes	N/A	N/A	N/A	N/A
Disposed/E	No	No	No	Yes	N/A	N/A	N/A	N/A
	No	No	No	No	N/A	N/A	N/A	N/A
	No	No	No	No	N/A	N/A	N/A	N/A

	No	No	No	Yes	N/A	N/A	N/A	N/A
	No	No	No	Yes	N/A	N/A	N/A	N/A
Disposed/E	No	No	No	Yes	N/A	No	N/A	N/A
Disposed/E	No	No	No	Yes	N/A	No	N/A	N/A
	No	No	No	Yes	No	No	No	No
Consume	No	No	No	Yes	N/A	No	N/A	N/A
Disposed/E	No	No	Yes	No	N/A	N/A	N/A	N/A
	No	No	Yes	No	N/A	N/A	N/A	N/A
	No	No	Yes	No	N/A	N/A	N/A	N/A
Disposed/E	No	No	Yes	No	Yes	No	No	No
	No	No	Yes	No	N/A	N/A	N/A	N/A
	No	No	No	No	No	No	No	No
	No	No	No	No	No	No	No	No
	No	No	No	No	No	No	No	No
	No	No	No	Yes	No	No	No	No
Disposed/E	No	No	No	No	No	No	No	No
Disposed/E	No	No	No	Yes	N/A	No	N/A	N/A
	No	No	Yes	Yes	N/A	N/A	N/A	N/A
	No	No	Yes	Yes	N/A	N/A	N/A	N/A
	No	No	No	No	N/A	No	N/A	N/A
	No	No	Yes	No	N/A	N/A	N/A	N/A
	No	No	Yes	No	N/A	N/A	N/A	N/A
	No	No	Yes	No	No	N/A	N/A	N/A
	No	No	No	Yes	N/A	No	N/A	N/A
	No	No	Yes	No	N/A	N/A	N/A	N/A
	No	No	No	Yes	N/A	No	N/A	N/A
	No	No	No	Yes	N/A	No	N/A	N/A
	No	No	No	Yes	N/A	No	N/A	N/A
	No	No	No	Yes	N/A	No	N/A	N/A
	No	No	No	Yes	N/A	No	N/A	N/A
Disposed/E	No	No	No	Yes	N/A	No	N/A	N/A
Disposed/E	No	No	No	Yes	No	No	No	No
Disposed/E	No	No	No	Yes	No	No	No	No
	Yes	No	No	Yes	Yes	No	No	No
	Yes	No	No	No	No	Yes	No	No
	Yes	No	No	No	Yes	No	No	No
	Yes	No	No	No	Yes	No	No	No
Fed Poultry	Yes	No	No	No	Yes	No	No	No
	Yes	No	No	No	No		Yes	No
	Yes	No	No	No	Yes	No	No	No
Disposed/E	Yes	No	No	No	Yes	No	No	No
Fed Poultry	Yes	No	No	No	Yes	No	No	No
	Yes	No	No	No	Yes	No	No	No
Disposed/E	Yes	No	No	No	Yes	No	No	No
	Yes	No	No	No	Yes	No	No	No
	Yes	No	No	No	Yes	No	No	No
	Yes	No	No	No	Yes	No	No	No

Disposed/E	Yes	No	No	No	Yes	No	No	No
Disposed/E	Yes	No	No	No	Yes	No	No	No
	Yes	No	No	No	Yes	No	No	No
Disposed/E	Yes	No	No	No	Yes	No	No	No
	Yes	No	No	No	Yes	No	No	No
	Yes	No	No	No	Yes	No	No	No
	Yes	No	No	No	Yes	No	No	No
Disposed/E	Yes	No	No	No	No	Yes	No	No
Disposed/E	Yes	No	No	No	Yes	No	No	No
Disposed/E	Yes	No	No	Yes		Yes	No	No
Fed Poultry	Yes	No	No	No		Yes	No	No
Disposed/E	Yes	No	No	No	No	Yes	No	No
Disposed/E	Yes	No	No	No		No	No	No
Disposed/E	Yes	No	No	No	No	Yes	No	No
Disposed/E	Yes	No	No	Yes	No	Yes	No	No
	Yes	No	No	No	Yes	No	No	No
Consume	Yes	No	No	No	Yes	No	No	No
Disposed/E	Yes	No	No	No	Yes	No	No	No
Disposed/E	Yes	No	No	No	Yes	No	No	No
Disposed/E	Yes	No	Yes	No	Yes	No	No	No
Disposed/E	Yes	No	Yes	No	Yes	No	No	No
Disposed/E	Yes	No	Yes	No	Yes	No	No	No
Disposed/E	Yes	No	Yes	No	Yes	No	No	No
Disposed/E	Yes	No	Yes	No	Yes	No	No	No
Disposed/E	Yes	No	No	No	Yes	No	No	No
Disposed/E	Yes	No	Yes	No	Yes	No	No	No
	Yes	No	Yes	No	Yes	No	No	No
	Yes	No	Yes	No	No	No	No	No
Disposed/E	Yes	No	No	No	No	Yes	No	No
	Yes	No	Yes	No	No	Yes	No	No
	Yes	No	Yes	No	No	Yes	No	No
	Yes	No	No	No	Yes	No	No	No
	Yes	No	No	No	Yes	No	No	No
	Yes	No	Yes	Yes	Yes	No	No	No
	Yes	No	No	No	No	Yes	No	No
Disposed/E	Yes	No	No	No	Yes	No	No	No
Consume	Yes	No	No	No	Yes	No	No	No
	Yes	No	Yes	Yes	Yes	No	No	No
	Yes	No	No	No	Yes	No	No	No
	Yes	No	No	No	Yes	Yes	No	No
	Yes	No	No	No	Yes	No	No	No
Disposed/E	Yes	No	No	No	Yes	No	No	No
	Yes	No	No	No	Yes	No	No	No
Disposed/E	Yes	No	No	No	Yes	No	No	No
	Yes	No	No	No	Yes	No	No	No
	Yes	No	No	No	Yes	No	No	No
Disposed/E	Yes	No	No	No	No	Yes	No	No

Fed Poultry	Yes	No	No	No	No	Yes	No	No
	Yes	No	No	No	No	Yes	No	No
	Yes	No	No	No	No	Yes	No	No
Disposed/E	Yes	No	No	No	No	Yes	No	No
	Yes	No	No	No	No	Yes	No	No
Disposed/E	Yes	No	No	No	No	Yes	No	No
	Yes	No	No	No	No	Yes	No	No
	Yes	No	No	No	No	Yes	No	No
	Yes	No	No	No	No	Yes	No	No
	Yes	No	No	No	No	Yes	No	No
	Yes	No	No	No	No	Yes	No	No
	Yes	No	No	No	No	Yes	No	No
	Yes	No	No	No	No	Yes	No	No
Disposed/E	Yes	No	No	Yes	No	No	No	No
Disposed/E	Yes	No	No	No	Yes	No	No	No
	Yes	No	No	No	No	No	No	No
Disposed/E	Yes	No	No	No	No	No	No	No
Disposed/E	Yes	No	No	No	No	No	No	No
Disposed/E	Yes	No	No	No	No	Yes	No	No
Disposed/E	Yes	No	No	No	Yes	No	No	No
Disposed/E	Yes	No	No	No	Yes	No	No	No
	Yes	No	No	No	No	No	No	No
Disposed/E	Yes	No	No	No	Yes	No	No	No
	Yes	No	No	No	Yes	No	No	No
	Yes	No	No	No	Yes	No	No	No
	Yes	No	No	No	Yes	No	No	No
	Yes	No	No	No	Yes	No	No	No
	Yes	No	No	No	Yes	No	No	No
Disposed/E	Yes	No	No	No	Yes	No	No	No
Disposed/E	Yes	No	No	No	No	Yes	No	No
Disposed/E	Yes	No	No	No	Yes	No	No	No
	Yes	No	No	Yes	No	Yes	No	No
Disposed/E	Yes	No	No	Yes	No	No	No	Yes
	Yes	No	No	Yes	Yes	No	No	No
Fed cows	Yes	No	No	No	Yes	N/A	No	No
Disposed/E	Yes	No	No	No	Yes	No	No	No
Disposed/E	Yes	No	No	No	No	Yes	No	No
Disposed/E	Yes	No	No	Yes	No	Yes	No	No
Disposed/E	Yes	No	No	No	No	Yes	No	No
	Yes	No	No	Yes	No	Yes	No	No
	No	No	No	Yes	N/A	N/A	N/A	N/A
Fed cows	Yes	No	No	Yes	No	Yes	No	No
Mix with g	Yes	No	No	Yes	No	No	No	Yes
Fed cows	Yes	No	No	No	Yes	No	No	No
Fed cows	Yes	No	No	No	Yes	No	No	No
Disposed/E	No	No	No	Yes	N/A	No	N/A	N/A
Disposed/E	No	No	No	Yes	N/A	N/A	N/A	N/A

Disposed/E	Yes	No	No	No	Yes	No	No	No
	Yes	No	No	Yes	No	Yes	No	No
Disposed/E	Yes	No	No	No	No	Yes	No	No
Disposed/E	Yes	No	No	No	Yes	No	No	No
Fed Poultry	Yes	No	No	No	No	Yes	No	No
Disposed/E	Yes	No	No	No	No	Yes	No	No
Fed cows	Yes	No	No	No	No	Yes	No	No
Fed cows	Yes	No	No	No	Yes	No	No	No
Disposed/E	Yes	No	No	Yes	No	No	No	Yes
Disposed/E	No	No	No	Yes	N/A	N/A	N/A	N/A
	No	No	No	Yes	N/A	N/A	N/A	N/A
	Yes	No	No	No	No	Yes	No	No
Disposed/E	Yes	No	No	Yes	No	Yes	No	No
Fed cows	Yes	No	No	No	Yes	No	No	No
Fed cows	Yes	No	No	No	Yes	No	No	No
Fed cows	Yes	No	No	No	No	Yes	No	No
Fed cows	Yes	No	No	No	No	Yes	No	No
Fed cows	Yes	No	No	No	Yes	No	No	No
Disposed/E	Yes	No	No	No	Yes	No	No	No
	Yes	No	No	No	Yes	No	No	No
Disposed/E	Yes	No	No	No	Yes	No	No	No
	Yes	No	No	No	Yes	No	No	No
Disposed/E	Yes	No	No	Yes	No	No	No	No
Disposed/E	Yes	No	No	No	Yes	No	No	No
	Yes	No	No	Yes	No	No	No	No
Fed cows	Yes	No	No	No	No	Yes	No	No
Sold	Yes	No	No	No	No	Yes	No	No
Disposed/E	Yes	No	No	No	Yes	No	No	No
Fed cows	No	No	No	Yes	N/A	N/A	N/A	N/A
	Yes	No	No	No	No	Yes	No	No
	Yes	No	No	No	No	Yes	No	No
Disposed/E	Yes	No	No	No		Yes	No	No
	Yes	No	No	No	Yes	Yes	No	No
Disposed/E	Yes	No	No	No	Yes	No	No	No
Disposed/E	Yes	No	No	Yes	Yes	No	No	No
	Yes	No	No	No	Yes	No	No	No
	Yes	No	No	No	No	Yes	No	No
	No	No	No	Yes	N/A	N/A	N/A	N/A
Disposed/E	No	No	No	Yes	N/A	N/A	N/A	N/A
Disposed/E	No	No	No	Yes	N/A	N/A	N/A	N/A
Disposed/E	Yes	No	No	No	Yes	No	No	No
	Yes	No	No	Yes	Yes	No	No	No
	Yes	No	No	Yes	No	Yes	No	No
Disposed/E	Yes	No	No	Yes	No	Yes	No	No
Fed cows	Yes	No	No	No	No	Yes	No	No
Disposed/E	Yes	No	No	No	Yes	No	No	No
Fed Poultry	Yes	No	No	No	No	Yes	No	No

Disposed/E	Yes	No	No	Yes	No	No	No	Yes
	Yes	No	No	No	No	Yes	No	No
Fed cows	Yes	No	No	No	No	Yes	No	No
Disposed/E	No	No	No	Yes	N/A	No	N/A	N/A
	Yes	No	No	Yes	Yes	No	No	No
Fed Poultry	Yes	No	No	No	No	Yes	No	No
Fed cows	Yes	No	No	No	No	Yes	No	No
Disposed/E	Yes	No	No	No	Yes	No	No	No
	Yes	No	No	Yes	No	Yes	No	No
Fed Poultry	Yes	No	No	No	No	Yes	No	No
Fed Poultry	Yes	No	No	No	No	Yes	No	No
Disposed/E	No	No	No	Yes	N/A	N/A	N/A	N/A
	Yes	No	No	No	Yes	No	No	No
Fed cows	No	No	No	Yes	N/A	N/A	N/A	N/A
Fed cows	No	No	No	Yes	N/A	N/A	N/A	N/A
	No	No	No	Yes	N/A	No	N/A	N/A
	Yes	No	No	No	Yes	No	No	No
	Yes	No	No	No	No	No	No	No
Disposed/E	Yes	No	No	No	No	Yes	No	No
	Yes	No	No	Yes	No	No	No	No
Disposed/E	Yes	No	No	No	Yes	No	No	No
Disposed/E	Yes	No	No	No	No	Yes	No	No
Fed cows	No	No	No	Yes	N/A	N/A	N/A	N/A
Fed Poultry	No	No	No	Yes	N/A	N/A	N/A	N/A
Disposed/E	Yes	No	No	No	Yes	No	No	No
	Yes	No	No	Yes	No	Yes	No	No
	Yes	No	No	Yes	No	Yes	No	No
	Yes	No	No	Yes	No	Yes	No	No
Disposed/E	Yes	No	No	Yes	No	Yes	No	No
Fed Poultry	Yes	No	No	No	No	Yes	No	No
Used for Br	Yes	No	No	No	No	Yes	No	No
Consume	Yes	No	No	No	Yes	Yes	No	No
Fed cows	Yes	No	No	No	Yes	Yes	No	No

none_cont	other_cont	quantity_p	immediate	which_mor	many_mor	month_firs	month_last	not_coveri
No	N/A	0	26	November	30	January		Yes
No	N/A	90	12	February		February	Still in stor	Yes
No	N/A	0						No
No	N/A	90	3	January				No
No	N/A	90	5	Augast	180	August	December	No
No	N/A	45	2	Augast	90	August	November	No
No	N/A	90	8	September	270	September	May	No
No	N/A	45	5	Augast	210	August	March	No
No	N/A	90	11	Augast	270	August	April	No
No	N/A	90	5	Augast	150	August	January	No
No	N/A	45	4	July	150	July	December	No
No	N/A	45	7	July	240	July	February	No
No	N/A	0	1					No
No	N/A	90	7	Augast	270	September	May	No
No	N/A	90	4	Augast	270	September	May	No
No	N/A	90	7	Augast	240	August	February	No
No	N/A	45	4	Augast	150	August	December	No
No	N/A	90	3	Augast	90	August	November	No
No	N/A	90	3	Augast	150	August	December	No
No	N/A	90	3	Augast	150	August	December	No
No	N/A	90	5	Augast	180	August	February	No
No	N/A	90	9	Augast	240	August	March	No
No	N/A	90	7	July	270	July	March	No
No	N/A	45	2	July	150	July	December	No
No	N/A	45	6	July	240	July	March	No
No	N/A	45	3	Augast	120	August	December	No
No	N/A	275	2	October	60	October	November	No
No	N/A	90	1	November	150	December	Still in stor	No
No	N/A	45						No
No	N/A	10	5	November	90	November	February	No
No	N/A	720	3	October	30	November	March	No
No	N/A	0		November	60	December		Yes
No	Agroz bags	45	4	December	60	January	February	Yes
No	N/A	90	10	November	30	November	March	Yes
No	N/A	23	1	December	90	January	March	No
No	N/A	90	7	December	30	January		Yes
No	N/A	45	7	December		December	Still in stor	Yes
No	N/A	180	9	September	240	September	April	No
No	N/A	90	7	July	240	July	February	No
No	N/A	90	6	July	225	July	February	No
No	N/A	90	4	Augast	180	August	January	No
No	N/A	180	8	Augast	270	August	April	No
No	N/A	45	2	Augast	120	August	December	No
No	N/A	90	13	Augast	300	August	May	No
No	N/A	90	8	July	270	July	March	No
No	N/A	45	6	November	210	November	June	No

No	N/A	90	4	Augast	150	August	December	No
No	N/A	45	4	Augast	120	August	November	No
No	N/A	90	7	Augast	180	August	January	No
No	N/A	180						No
No	N/A	45	2	Augast	150	August	December	No
No	N/A	180	12	September	210	September	March	No
No	N/A	90	5	September	240	September	May	No
No	N/A	45	4	July	210	July	February	No
No	N/A	180	2	September	60	January		No
No	N/A	0	18	December	180	December		No
No	N/A	0	3	November	90	November	January	No
No	N/A	90	15	November	150	December	Still in store	No
No	N/A	90	3	November	30	December	February	No
No	N/A	16	5	October	30	November	Still in store	No
No	N/A	16	4	November	15	January	Still in store	No
No	N/A	90	8	November	120	January	Still in store	No
No	N/A	0						Yes
No	N/A	0						Yes
No	N/A	90	4	October	120			Yes
No	N/A	0						Yes
No	N/A	108	7	November	60		January	Yes
No	N/A	450	15	December	120	March		Yes
No	N/A	90	16	October	30	December	Still in store	Yes
No	N/A	540	14	October	30	January		Yes
No	N/A	180	10	January		February		Yes
No	N/A	90	12	December	30	January		Yes
No	N/A	180	19	November	60	January		Yes
No	N/A	180	19	November	30	January		Yes
No	N/A	90	12	December	30	January		Yes
No	N/A	180	10	January		February		Yes
No	N/A	180	10	January		February		Yes
No	Agroz bags	450	7	December		December	Still in store	No
No	N/A	45	3	December		December	Still in store	Yes
No	N/A	90	7	October		October	Still in store	Yes
No	N/A	90	10	November		November	Still in store	Yes
No	N/A	90	3	October	90	October	April	Yes
No	N/A	180	5	November	90	November	April	Yes
No	N/A	45	13	December		December	Still in store	Yes
No	Agroz bags	0	1	April	30	April	March	No
No	N/A	0						Yes
No	N/A	0						No
No	N/A	0	2	December	30	January	February	Yes
No	N/A	180	5	April	30	April	May	No
No	N/A	0	1		60			No
No	N/A	0	1					No
Yes	N/A	10	1	April	120	April	April	Yes
No	Agroz bags	0	2	March	120	April		No

No	N/A	45	16					No
No	Agroz bags	15	2	March	75			No
No	N/A	10	26		120		Still in stor	Yes
No	N/A	5	1		15			No
No	Agroz bags	0	90				January	No
No	N/A	113	1		7			Yes
No	N/A	450						Yes
No	N/A	0						No
No	N/A	0						Yes
No	N/A	0						Yes
No	N/A	0						No
No	N/A	0					March	No
No	N/A	0						No
No	N/A	0						Yes
No	N/A	0	30	April	90	April	January	No
No	Agroz bags	0	30	April	330	April	Still in stor	No
No	N/A	900	80	March	30	March	April	No
No	N/A	0	1		7			No
No	N/A	0	4					Yes
Yes	N/A	0	4		30			No
No	N/A	0	1		120			No
No	N/A	0	30		30			No
No	N/A	0	2					No
No	N/A	0	1	February		March	March	No
No	N/A	0	1	March		March	Still in stor	Yes
No	N/A	450	6	February	90	February	Still in stor	Yes
No	N/A	0	1	February	30	February	March	Yes
No	N/A	0	4	November	90	November	February	Yes
No	N/A	90	9					Yes
No	N/A	180	4	February	90	February	Still in stor	Yes
No	N/A	0	1	March	90			No
No	N/A	0	1	March	30	March	April	Yes
No	N/A	2	1	March	30	March	Still in stor	Yes
No	N/A	0	2	Augast	30	May		No
No	N/A	0	4	April	60	April	May	No
No	N/A	0						No
No	N/A	0	3	March	30	April	April	No
No	N/A	0						No
No	N/A	0	20					No
No	N/A	0	90	April	120	April	May	Yes
No	N/A	3	60	April	120	April		Yes
No	N/A	2	40	April	120	April		Yes
No	N/A	10	20	May	150	May		Yes
No	N/A	2	1	April	120	April	May	No
No	N/A	0	0					No
No	N/A	10	1	April	120	April		No
No	N/A	20	5	April	120	April		Yes

No	N/A	0	5	April	30	May		No
No	N/A	0	4	April	30	April	May	No
No	N/A	0	2	April	30	May		No
No	N/A	0						No
No	N/A	0	1					No
No	N/A	0	1					No
No	N/A	0						No
No	N/A	23	3		180			Yes
No	N/A	45	1		60			Yes
No	Agroz bags	0	6		75		Still in store	Yes
No	Agroz bags	3	1				Still in store	Yes
No	N/A	45	2				Still in store	Yes
No	Agroz bags	0	1		60			Yes
No	N/A	2	2	April	120			No
No	N/A	0	4	November	120	November	May	No
No	N/A	0			0			No
No	N/A	4	2		45			Yes
No	N/A	0	1	December	60	December	December	Yes
No	N/A	10	2	December	60	January	January	Yes
No	N/A	0		December	30	January	February	Yes
No	N/A	0						Yes
No	N/A	0						Yes
No	N/A	0		December	30	January	February	Yes
No	N/A	0						Yes
No	N/A	0	1	December	30	January		Yes
No	N/A	0	1					Yes
No	N/A	0						Yes
No	N/A	0						No
No	N/A	20	60	March	30	March	April	Yes
No	N/A	36	8					No
No	N/A	0	7					Yes
No	N/A	0	5					Yes
No	N/A	0	1					No
No	Agroz bags	0	12					Yes
No	Agroz bags	0	5					Yes
No	N/A	10	1	February	60	February	March	Yes
No	N/A	5	1	February	30	February	March	Yes
No	N/A	0	1	January	60	January	February	No
No	N/A	0	1	February	45	February	March	Yes
No	N/A	30	270	June	90	July	October	No
No	N/A	40	200					No
No	N/A	450	2	March	30	March	April	Yes
No	Agroz bags	5	2	February	180			Yes
No	N/A	5	2	February	90	February	Still in store	Yes
No	N/A	10	30		60		Still in store	Yes
No	N/A	90	4	February	60	February	April	No
No	N/A	270	12	February		February	Still in store	Yes

No	N/A	0	2	March	60	March	April	Yes
No	Agroz bags	45	4	March	60	March	Still in store	Yes
No	N/A	7	1	February	90	February	April	Yes
No	N/A	40	3	February	4	February	Still in store	Yes
No	N/A	36	3	February	60	February	Still in store	Yes
No	N/A	135	0	February	90	February	Still in store	Yes
No	N/A	24	8	February	60	February	Still in store	Yes
No	N/A	0	3	February	90	February	Still in store	Yes
No	Agroz bags	3	2	February	60	February	April	Yes
No	N/A	0	1	February	30	February	April	Yes
No	N/A	0	2	March		March	Still in store	Yes
No	N/A	90	1	February	30	February	Still in store	Yes
No	N/A	0	1	March	60	March	April	Yes
No	Agroz bags	5	2	February	60	February		Yes
No	N/A	20	1	February		February	December	Yes
No	Agroz bags	40	1	February	180	September	December	Yes
No	Agroz bags	45	2	March	90			Yes
No	Agroz bags	0	1	March			Still in store	Yes
No	N/A	270	2	February		February	Still in store	Yes
No	N/A	23	2	February		February	Still in store	Yes
No	N/A	0	4	March			Still in store	Yes
No	Agroz bags	0	1	February		February	Still in store	Yes
No	N/A	90	2	March	60	March	April	Yes
No	N/A	0	3	February	90	March	Still in store	Yes
No	N/A	0	2	February		February	March	Yes
No	N/A	0	5	February		April	September	Yes
No	Agroz bags	0	26	March	150		December	Yes
No	N/A	0	1	March	150		January	Yes
No	N/A	0						No
No	N/A	90	160	January	90	February		Yes
No	N/A	23	25	December	180	December		No
No	N/A	0	35	December	150	December	Still in store	No
No	N/A	360	50	December	270	February		Yes
No	N/A	0	54	December	210	January		No
No	N/A	90	20	November	150	December	Still in store	No
No	N/A	450	25	November	150	December	Still in store	No
No	N/A	68	20	November	150	January	Still in store	No
No	N/A	90	20	November	90	January	Still in store	No
No	N/A	135	28	November	120	January	Still in store	No
No	N/A	0	30	April	30		May	No
No	N/A	90	12	August	150	August	December	No
No	N/A	45	30	December	210	January	Still in store	Yes
No	N/A	90	10	January	120	January		Yes
No	N/A	180	10	November		November	Still in store	Yes
No	N/A	90	9	November		November	Still in store	Yes
No	N/A	0	2	March			Still in store	No
No	N/A	0	24	November	90	November	February	No

No	N/A	90	30					Yes
No	N/A	0	40	December	180	December	Still in stor	No
No	N/A	270	150	November	225	March	November	Yes
No	N/A	90	50	November	120	December	Still in stor	No
No	N/A	45	28	November	30	December	Still in stor	No
No	N/A	90	30	October	120	January	Still in stor	No
No	N/A	135	30	November	60	March		Yes
No	N/A	180	32	February	120	June		Yes
No	N/A	135	74	November	240	February		Yes
No	N/A	0	30	November	150	December	Still in stor	No
No	N/A	360	10	December	120	December	March	No
No	N/A	135	10	July	240	August	February	No
No	N/A	45	13	December	270	December		Yes
No	N/A	16	7	November	150	December	February	No
No	N/A	180	8	October	90		January	Yes
No	N/A	90	12	December		March		Yes
No	N/A	450	3	October		October	October	Yes
No	N/A	0	5	November		November	Still in stor	Yes
No	N/A	0					Still in stor	Yes
No	N/A	0	20	December	30	February	April	Yes
No	N/A	45	38					Yes
No	N/A	0	40	November	120	November	February	No
No	N/A	0						No
No	N/A	0	40	November	120	November	Still in stor	No
No	N/A	0						No
No	N/A	180	38	November	30		February	No
No	N/A	0	47	November	30	January		Yes
No	N/A	3	10	March		March	April	No
No	N/A	0		September	90	October	December	No
No	N/A	180	14	July	300	July	May	No
No	N/A	90	14	Augast	330	August	June	No
No	N/A	0	5	Augast	30	May	May	No
No	N/A	0	6	April	30	May	May	No
No	N/A	40	5	March	180	November	January	No
No	N/A	90	14	November	210	December		No
No	N/A	0	40	November		December	Still in stor	No
No	N/A	135	56	November	30	January	March	Yes
No	N/A	0	20	November	150	December	Still in stor	No
No	N/A	90	25	November	120	December	Still in stor	No
No	N/A	450	10	December	60	January	Still in stor	No
No	N/A	90	20					Yes
No	N/A	0	22	December	180	December		No
No	N/A	0	30	November	150	December	Still in stor	No
No	N/A	0	34	November	210	January		Yes
No	N/A	14	10	November	150	December	Still in stor	No
No	N/A	60	10	November	120	November	April	No
No	N/A	16		October	30	November	Still in stor	No

No	N/A	90	8	November	60	December	April	No
No	N/A	0						Yes
No	N/A	450	30	November	90	February	September	Yes
No	N/A	0	10	March	90		Still in stor	No
No	N/A	189	21	December	180	December		No
No	N/A	90	8	October	90	December	March	No
No	N/A	180	5	September	30		Still in stor	Yes
No	N/A	180	29					Yes
No	N/A	0	23	November	150	December	Still in stor	No
No	N/A	135	14	December	30	January		Yes
No	N/A	135	14	December	30	January		Yes
No	N/A	0	20	November	120	November	Still in stor	No
No	N/A	0	15	March	60	April	April	No
No	N/A	16	3	November	150	December		No
No	N/A	540	9			August	January	No
No	N/A	0	5	April		April	Still in stor	Yes
No	N/A	0	5	April	30	May	May	No
No	N/A	0	100		90			No
No	N/A	90	4	March	30	March	Still in stor	Yes
No	Agroz bags	0	3	April	30	April	Still in stor	Yes
No	N/A	68	10	March		March	Still in stor	Yes
No	N/A	450	8		120			Yes
No	N/A	60	20	November	90	November	January	No
No	N/A	16	15	November	150	January	Still in stor	No
No	N/A	0	30	December	120	December		No
No	N/A	0	30	December	150	November	Still in stor	No
No	N/A	0	26	December	150	December	Still in stor	No
No	N/A	0	40	November	150	November	Still in stor	No
No	N/A	0	40	November	270	December		Yes
No	N/A	270	5	December	150	March	June	Yes
No	N/A	90	20	December		December		Yes
No	N/A	90	10	November		November		Yes
No	N/A	720	50	December		December	Still in stor	Yes

covering_w	bring_hom	commercia	green_mai:	dry_off_grc	keep_maiz	shelling_dr	method_dr	dehusked_
No	No	No	No	No	30	4	Sun drying	Never
Yes	No	No	Yes	Yes	30	2	Sun drying	Frequent
No	No	No	Yes	No	7	15	Sun drying	Never
No	Yes	No	No	No	21	1	Airing	Never
No	Yes	No	No	No	14	1	Airing	Never
No	Yes	No	No	No	37	14	Airing	Never
No	Yes	No	No	No	7	7	Airing	Never
No	Yes	No	No	No	14	7	Airing	Never
No	Yes	No	No	No	14	7	Airing	Never
No	Yes	No	No	No	7	4	Airing	Never
No	Yes	No	No	No	7	2	Sun drying	One week
No	Yes	No	No	No	7	14	Sun drying	Never
No	Yes	No	No	No	7	1	Airing	Never
No	Yes	No	No	No	7	4	Sun drying	Never
No	Yes	No	No	No	7	14	Sun drying	Never
No	Yes	No	No	No	7	4	Airing	Never
No	Yes	No	No	No	1	3	Sun drying	Never
No	Yes	No	No	No	3	7	Airing	Never
No	Yes	No	No	No	4	7	Airing	Never
No	Yes	No	No	No	4	2	Airing	Never
No	Yes	No	No	No	7	7	Sun drying	Never
No	Yes	No	No	No	7	7	Sun drying	Never
No	Yes	No	No	No	14	7	Sun drying	Never
No	Yes	No	No	No	4	2	Sun drying	Never
No	Yes	No	No	No	7	4	Sun drying	Never
No	Yes	No	No	No	4	3	Airing	Never
No	Yes	No	Yes	No	30	21	Sun drying	Always
No	Yes	No	No	No	90	2	Sun drying	Never
No	Yes	No	No	No	30	3	Sun drying	Never
No	No	No	Yes	Yes	90	1	Sun drying	Frequent
No	Yes	No	No	No	30	2	Sun drying	Never
No	No	No	No	No	60	7	Sun drying	Never
No	No	No	No	No	30	3	Sun drying	Never
No	No	No	No	No	30	3	Sun drying	Never
Yes	No	No	No	No	60	7	Sun drying	One month
No	No	No	No	No	30	5	Sun drying	One month
Yes	No	No	Yes	Yes	30	14	Sun drying	Frequent
No	Yes	No	No	No	12	2	Airing	Never
No	Yes	No	No	No	35	4	Airing	Never
No	Yes	No	No	No	14	4	Airing	Never
No	Yes	No	No	No	28	1	Airing	Never
No	Yes	No	No	No	7	1	Airing	One week
No	Yes	No	No	No	7	21	Airing	Never
No	Yes	No	No	No	7	6	Airing	Never
No	Yes	No	No	No	21	7	Sun drying	One week
No	Yes	No	No	No	14	7	Airing	Never

No	Yes	No	No	No	7	4	Airing	Never
No	Yes	No	No	No	7	14	Sun drying	Never
No	Yes	No	No	No	7	7	Airing	Never
No	Yes	No	No	No	30	7	Sun drying	Never
No	Yes	No	No	No	1	7	Sun drying	Never
No	Yes	No	No	No	14	14	Airing	Never
No	Yes	No	No	No	21	7	Airing	Never
No	Yes	No	No	No	1	1	Sun drying	Never
No	Yes	No	No	No	7	14	Sun drying	Never
Yes	Yes	No	No	No	14	14	Sun drying	Never
No	No	No	Yes	Yes	7	7	Sun drying	Frequent
No	Yes	No	No	No	90	2	Sun drying	Never
No	Yes	No	No	No	30	2	Sun drying	Never
No	Yes	No	No	No	30	2	Sun drying	Never
No	Yes	No	No	No	14	3	Sun drying	Never
No	Yes	No	No	No	120	3	Sun drying	Never
No	No	No	No	No	30	14	Sun drying	Never
No	No	No	No	No	7	14	Sun drying	Never
No	No	No	No	No	14	14	Sun drying	Never
No	No	No	No	No	30	3	Sun drying	Never
No	No	No	No	No	60	7	Sun drying	Two Montl
No	No	No	No	No	14	30	Sun drying	Never
No	No	No	No	No	30	10	Sun drying	Never
No	No	No	No	No	7	7	Sun drying	One montl
No	No	No	No	No	2	5	Sun drying	Never
No	No	No	No	No	30	3	Sun drying	One montl
No	No	No	No	No	30	5	Sun drying	One week
No	No	No	No	No	30	4	Sun drying	One montl
No	No	No	No	No	30	3	Sun drying	One montl
No	No	No	No	No	5	5	Sun drying	Never
No	No	No	No	No	2	5	Sun drying	Never
No	Yes	No	Yes	Yes	30	5	Sun drying	Frequent
No	Yes	No	Yes	Yes	30	2	Sun drying	Never
No	No	No	Yes	Yes	14	3	Sun drying	Frequent
Yes	No	No	Yes	Yes	60	2	Sun drying	Frequent
No	No	No	Yes	Yes	2	14	Sun drying	Never
Yes	No	No	Yes	Yes	30	5	Sun drying	Frequent
Yes	No	No	Yes	Yes	30	2	Sun drying	Frequent
Yes	No	No	No	No	7	14	Sun drying	Never
No	No	No	No	No	0	30	Sun drying	Never
Yes	No	No	No	No	20	4	Sun drying	Never
No	No	No	No	No	60	30	Sun drying	One montl
No	Yes	No	No	No	14	30	Sun drying	Never
No	Yes	No	Yes	Yes	30	7	Sun drying	Never
No	Yes	No	Yes	Yes	30	7	Sun drying	Frequent
Yes	Yes	No	No	Yes	30	30	Airing	Never
Yes	Yes	No	No	Yes	30	30	Airing	Never

No	Yes	No	No	Yes	0	2	Sun drying	Never
No	No	No	No	Yes	60	3	Sun drying	Never
Yes	No	No	Yes	Yes	120	2	Sun drying	Frequent
No	No	No	Yes	Yes	30	7	Sun drying	Frequent
No	Yes	Yes	No	No	0	30	Sun drying	Never
Yes	No	No	Yes	Yes	30	3	Sun drying	Never
No	No	No	No	Yes	7	3	Airing	Frequent
No	No	No	Yes	No	14	2	Sun drying	Frequent
No	Yes	No	No	No	42	7	Sun drying	Frequent
No	No	No	No	No	21	7	Sun drying	One week
Yes	No	No	Yes	No	20	4	Sun drying	Frequent
Yes	Yes	No	No	Yes	21	2	Sun drying	Never
Yes	Yes	No	Yes	Yes	30	21	Sun drying	Frequent
No	Yes	No	Yes	Yes	7	21	Sun drying	Never
Yes	Yes	No	No	Yes	0	30	Sun drying	Never
Yes	Yes	No	No	Yes	0	30	Sun drying	Never
Yes	Yes	No	No	No	30	21	Sun drying	Never
Yes	No	No	No	No	7	7	Airing	Frequent
No	No	No	No	Yes	21	3	Sun drying	Frequent
Yes	No	No	No	Yes	14	7	Airing	Frequent
Yes	No	No	No	No	7	14	Airing	Frequent
Yes	No	No	No	No	7	14	Airing	Frequent
Yes	Yes	No	Yes	No	21	21	Airing	Frequent
No	No	No	Yes	Yes	14	14	Sun drying	Never
No	No	No	Yes	Yes	21	2	Sun drying	Never
Yes	No	No	Yes	Yes	30	7	Sun drying	Never
Yes	No	No	Yes	Yes	30	2	Sun drying	Never
Yes	No	No	Yes	Yes	14	7	Sun drying	Frequent
Yes	No	No	Yes	Yes	14	7	Sun drying	Frequent
No	No	No	Yes	Yes	60	7	Sun drying	Frequent
No	No	No	Yes	Yes	30	1	Sun drying	Never
No	No	No	Yes	Yes	30	1	Sun drying	Never
Yes	No	No	Yes	Yes	30	7	Sun drying	Always
No	Yes	No	No	No	1	1	Sun drying	Never
No	Yes	No	No	No	14	30	Sun drying	Never
No	Yes	No	No	No	0	7	Sun drying	Never
No	Yes	No	No	No	14	30	Sun drying	Never
Yes	No	No	No	No	30	14	Airing	Never
Yes	No	No	No	No	7	7	Airing	Never
Yes	Yes	No	No	Yes	30	30	Sun drying	Never
Yes	Yes	No	No	Yes	30	30	Sun drying	Never
Yes	Yes	No	No	Yes	30	30	Sun drying	Never
No	Yes	No	No	Yes	30	30	Sun drying	Never
Yes	Yes	No	No	Yes	30	30	Sun drying	Never
Yes	Yes	No	No	Yes	30	30	Sun drying	Never
Yes	No	No	Yes	Yes	30	30	Sun drying	Never
Yes	Yes	No	No	Yes	30	30	Airing	Never

No	Yes	No	No	No	30	30	Sun drying	Never
No	Yes	No	No	No	30	21	Sun drying	Never
No	Yes	No	No	No	30	14	Sun drying	Never
No	Yes	No	No	No	30	30	Sun drying	Never
No	Yes	No	No	No	45	21	Sun drying	Never
No	Yes	No	No	No	60	30	Sun drying	Never
No	Yes	No	No	No	0	0	Sun drying	Never
Yes	No	No	Yes	Yes	30	7	Sun drying	Never
Yes	No	No	Yes	Yes	30	7	Sun drying	Frequent
Yes	No	No	Yes	Yes	60	7	Sun drying	Frequent
Yes	No	No	Yes	Yes	90	30	Sun drying	Frequent
Yes	No	No	Yes	Yes	120	7	Sun drying	Frequent
No	No	No	Yes	Yes	30	60	Sun drying	Never
Yes	No	No	Yes	Yes	30	30	Sun drying	Never
No	No	No	No	Yes	30	30	Sun drying	Never
Yes	Yes	No	No	Yes	21	3	Sun drying	Never
Yes	No	No	Yes	Yes	45	30	Sun drying	Frequent
No	No	No	No	No	0	30	Sun drying	One montł
No	No	No	No	No	14	7	Sun drying	One week
No	No	No	No	No	7	7	Sun drying	One week
No	No	No	No	No	30	7	Sun drying	Never
No	No	No	No	No	21	4	Sun drying	Never
No	No	No	No	No	7	7	Sun drying	Frequent
No	No	No	No	No	21	4	Sun drying	Frequent
No	No	No	No	No	0	7	Sun drying	Never
No	No	No	No	No	7	30	Sun drying	Frequent
No	No	No	No	No	30	7	Sun drying	Never
Yes	No	No	Yes	No	21	3	Sun drying	Frequent
No	No	Yes	No	Yes	30	7	Sun drying	Frequent
No	Yes	No	No	No	60	2	Sun drying	Frequent
No	Yes	No	No	Yes	21	1	Sun drying	Frequent
No	No	No	Yes	No	14	7	Sun drying	Frequent
Yes	No	No	No	No	30	30	Sun drying	Frequent
Yes	Yes	No	No	Yes	7	21	Sun drying	Never
Yes	Yes	No	No	Yes	30	21	Sun drying	Frequent
No	No	No	Yes	Yes	14	0	Sun drying	Frequent
Yes	No	No	Yes	Yes	30	14	Sun drying	Never
Yes	No	No	No	Yes	7	2	Airing	Frequent
Yes	No	No	Yes	Yes	30	3	Sun drying	Never
Yes	No	Yes	No	Yes	7	3	Airing	Frequent
Yes	No	No	No	No	30	7	Sun drying	Frequent
Yes	No	No	Yes	Yes	30	7	Sun drying	Frequent
Yes	No	No	Yes	Yes	21	2	Sun drying	Never
Yes	No	No	Yes	Yes	21	3	Sun drying	Frequent
Yes	No	No	Yes	Yes	30	14	Sun drying	Never
Yes	No	No	No	No	2	2	Sun drying	Never
Yes	No	No	Yes	Yes	14	4	Sun drying	Never

Yes	No	No	Yes	Yes	30	7	Sun drying	Never
Yes	No	No	Yes	Yes	30	2	Sun drying	Never
Yes	No	No	Yes	Yes	60	3	Sun drying	Frequent
Yes	No	No	Yes	Yes	30	7	Sun drying	Never
Yes	No	No	Yes	Yes	30	7	Sun drying	Never
Yes	No	No	Yes	Yes	30	7	Sun drying	Never
Yes	No	No	Yes	Yes	30	7	Sun drying	Never
Yes	No	No	Yes	Yes	30	2	Sun drying	Never
Yes	No	No	Yes	Yes	21	4	Sun drying	Never
Yes	No	No	Yes	Yes	30	2	Sun drying	Never
Yes	No	No	Yes	Yes	30	3	Sun drying	Never
Yes	No	No	Yes	Yes	60	4	Sun drying	Never
Yes	No	No	Yes	Yes	30	7	Sun drying	Never
No	No	No	Yes	Yes	3	2	Sun drying	Frequent
Yes	No	Yes	Yes	Yes	7	7	Sun drying	Never
Yes	No	No	Yes	Yes	14	7	Sun drying	Never
No	Yes	No	Yes	Yes	30	7	Sun drying	Never
No	Yes	No	Yes	Yes	30	7	Sun drying	Frequent
No	No	No	Yes	Yes	30	7	Sun drying	Frequent
No	No	No	Yes	Yes	60	7	Sun drying	Frequent
No	Yes	No	Yes	Yes	60	7	Sun drying	Never
Yes	No	No	Yes	Yes	30	30	Sun drying	Frequent
Yes	No	No	Yes	Yes	14	3	Sun drying	Always
Yes	No	No	Yes	Yes	30	14	Sun drying	Never
Yes	No	No	Yes	Yes	7	7	Sun drying	Frequent
No	No	Yes	No	Yes	30	7	Sun drying	Frequent
No	No	Yes	No	Yes	14	7	Sun drying	Never
No	No	Yes	No	Yes	21	7	Sun drying	Frequent
No	No	No	No	Yes	21	21	Sun drying	Never
No	No	No	No	No	60	14	Sun drying	Never
No	Yes	No	No	No	14	7	Sun drying	Never
Yes	Yes	No	No	No	14	14	Sun drying	Never
No	No	No	No	No	30	30	Sun drying	Never
No	Yes	No	No	No	30	7	Sun drying	Never
No	Yes	No	No	No	120	1	Sun drying	Frequent
No	Yes	No	No	No	90	2	Sun drying	Never
No	Yes	No	No	No	150	4	Sun drying	Never
No	Yes	No	No	No	90	2	Sun drying	Never
No	Yes	No	No	No	120	3	Sun drying	Never
No	Yes	No	No	No	21	21	Sun drying	Never
No	Yes	No	No	No	14	7	Airing	Never
No	No	No	No	No	21	4	Sun drying	Never
No	No	No	Yes	No	30	7	Sun drying	Never
Yes	No	No	Yes	Yes	60	14	Sun drying	Frequent
Yes	No	No	Yes	Yes	30	14	Sun drying	Frequent
Yes	Yes	No	No	Yes	30	21	Sun drying	Never
No	No	No	Yes	Yes	60	1	Sun drying	Always

Yes	Yes	No	Yes	Yes	30	21	Sun drying	Frequent	
No	Yes	Yes	No	No	14	21	Sun drying	Never	
No	Yes	No	No	No	18	4	Sun drying	Never	
No	Yes	No	No	No	120	3	Sun drying	Never	
No	Yes	No	No	No	30	2	Sun drying	Never	
No	Yes	No	No	No	120	2	Sun drying	Never	
No	No	No	No	No	30	3	Sun drying	One month	
No	No	No	No	No	4	3	Sun drying	Never	
No	Yes	No	No	No	18	14	Sun drying	Never	
No	Yes	No	No	No	90	2	Sun drying	Never	
No	Yes	No	Yes	Yes	90	2	Sun drying	Never	
No	Yes	No	No	No	7	1	Airing	Never	
No	No	No	No	No	30	6	Sun drying	Never	
No	Yes	No	No	No	120	2	Sun drying	Never	
No	No	No	No	No	90	3	Sun drying	Never	
No	No	No	No	No	30	3	Sun drying	One month	
Yes	No	No	Yes	Yes	3	5	Sun drying	Never	
Yes	No	No	Yes	Yes	30	4	Sun drying	Frequent	
Yes	No	No	Yes	Yes	30	21	Sun drying	Frequent	
Yes	No	No	No	No	14	7	Sun drying	Frequent	
Yes	Yes	No	Yes	Yes	60	30	Sun drying	Frequent	
No	No	No	Yes	Yes	60	1	Sun drying	Frequent	
Yes	Yes	No	No	No	14	5	Sun drying	Never	
No	No	No	Yes	Yes	90	2	Sun drying	Always	
No	Yes	No	Yes	No	120	7	Sun drying	Never	
Yes	No	No	No	No	30	7	Sun drying	One month	
No	No	No	No	No	60	14	Sun drying	One month	
No	Yes	No	No	No	60	30	Sun drying	Never	
No	Yes	No	No	No	30	30	Sun drying	Never	
No	Yes	No	No	No	30	14	Sun drying	Never	
No	Yes	No	No	No	30	7	Sun drying	Never	
No	Yes	No	No	No	60	30	Sun drying	Never	
No	Yes	No	No	No	60	30	Sun drying	Never	
No	No	Yes	No	Yes	14	7	Sun drying	Frequent	
No	Yes	No	No	No	75	14	Sun drying	Never	
No	Yes	No	No	No	120	1	Sun drying	Always	
No	No	No	No	No	14	4	Sun drying	One month	
No	Yes	No	No	No	90	2	Sun drying	Frequent	
No	Yes	No	No	No	150	2	Sun drying	Frequent	
No	Yes	No	Yes	Yes	30	2	Sun drying	Always	
Yes	Yes	No	Yes	Yes	60	7	Sun drying	Frequent	
No	Yes	No	No	No	14	14	Sun drying	Never	
No	Yes	No	No	No	7	7	Sun drying	Never	
No	No	No	No	No	45	4	Sun drying	Never	
No	Yes	No	No	No	90	4	Sun drying	Never	
No	No	No	Yes	Yes	60	3	Sun drying	Always	
No	Yes	No	No	No	30	2	Sun drying	Never	

No	Yes	No	No	No	60	3	Sun drying	Never
No	No	No	No	No	14	7	Sun drying	Never
No	No	No	No	No	30	30	Sun drying	One week
Yes	Yes	No	Yes	Yes	90	30	Sun drying	Never
Yes	Yes	No	No	No	7	14	Sun drying	Never
No	Yes	No	No	No	90	2	Sun drying	Never
No	No	No	No	No	30	30	Sun drying	Never
Yes	Yes	No	Yes	Yes	44	14	Sun drying	Always
No	Yes	No	No	No	14	7	Sun drying	Never
No	No	No	No	No	14	5	Sun drying	One month
No	No	No	No	No	14	5	Sun drying	One month
No	Yes	No	Yes	Yes	120	3	Sun drying	Always
No	Yes	No	No	No	30	30	Sun drying	Never
No	Yes	No	No	No	90	2	Sun drying	Never
No	No	No	Yes	No	90	7	Sun drying	One week
Yes	Yes	No	Yes	Yes	30	7	Sun drying	Never
No	Yes	No	No	No	1	21	Sun drying	Never
Yes	No	No	No	No	30	7	Airing	Frequent
Yes	No	No	Yes	Yes	30	7	Sun drying	Never
Yes	No	No	Yes	Yes	60	3	Sun drying	Never
No	No	No	Yes	Yes	30	7	Sun drying	Frequent
No	Yes	Yes	No	No	90	4	Sun drying	Never
No	No	No	Yes	Yes	60	1	Sun drying	Frequent
No	Yes	No	No	No	150	2	Sun drying	Never
Yes	Yes	No	No	No	90	7	Sun drying	Never
Yes	Yes	No	No	No	14	7	Sun drying	Never
Yes	Yes	Yes	No	No	14	7	Sun drying	Never
Yes	Yes	No	No	No	21	14	Sun drying	Never
No	No	No	No	No	30	3	Airing	Never
No	No	No	No	No	150	14	Sun drying	Never
No	No	No	No	No	60	7	Sun drying	Never
Yes	No	No	Yes	Yes	7	7	Sun drying	Frequent
Yes	No	No	Yes	Yes	2	2	Sun drying	Never

husk_stora	grain_stora	know_grair	dry_last_se	why_not_d	clean_stora	sale_maize	bags_maize	quality_sto
Never	Frequent	Hard when	1		Yes	No		
Frequent	Always	During shel	1		Yes	No		
Frequent	Never	Observe ch	0	Consumed	Yes	No	0	
Never	Always	Touching tl	1		Yes	No	0	
Never	Always	Hard when	1		Yes	No	0	
Never	Always	Hard when	1		Yes	No	0	
Never	Always	Hard when	1		Yes	No	0	
Never	Always	Touching tl	1		Yes	No	0	
Never	Always	Touching tl	1		Yes	No	0	
Never	Always	Touching tl	1		Yes	No	0	
Never	Always	Touching tl	1		Yes	No	0	
Never	Always	Hard when	1		Yes	No	0	
Never	Never	Hard when	1		Yes	No	0	
Never	Always	Touching tl	1		Yes	No	0	
Never	Always	Touching tl	1		Yes	No	0	
Never	Always	Hard when	1		Yes	No	0	
Never	Always	Hard when	1		Yes	No	0	
Never	Always	Touching tl	1		Yes	No	0	
Never	Always	Hard when	1		Yes	No	0	
Never	Always	Hard when	1		Yes	No	0	
Never	Always	Touching tl	1		Yes	No	0	
Never	Always	Hard when	1		Yes	No	0	
Never	Always	After one v	1		Yes	No	0	
Never	Always	Touching tl	1		Yes	No	0	
Never	Always	Hard when	1		Yes	No	0	
Never	Always	Hard when	1		Yes	No	0	
Never	Frequent	Observe ch	1		Yes	No	0	
Never	Frequent	Sound reco	1		Yes	No	0	
Never	Frequent	Sound reco	1		Yes	No	0	
Never	Frequent	Sound reco	1		Yes	No	0	
Never	Frequent	Hard when	1		Yes	No	0	
Never	Always	Hard when	1		Yes	No	0	
Never	Always	Hard when	1		Yes	No	0	
Never	Always	Hard when	1		Yes	No	0	
One montl	One Montl	Observe ch	1		Yes	No	0	
One montl	Always	Hard when	1		Yes	No	0	
Never	Frequent	During shel	1		Yes	No	0	
Never	Always	After one v	1		Yes	No	0	
Never	Always	Hard when	1		Yes	No	0	
Never	Always	Hard when	1		Yes	No	0	
Never	Always	Touching tl	1		Yes	No	0	
Never	Always	Touching tl	1		Yes	No	0	
Never	Always	Touching tl	1		Yes	No	0	
Never	Always	Touching tl	1		Yes	No	0	
Never	Never	After one v	1		Yes	No	0	
Never	Always	After one v	1		Yes	No	0	

Never	Always	Touching th	1		Yes	No	0	
Never	Always	Hard when	1		Yes	No	0	
Never	Always	Hard when	1		Yes	No	0	
Always	Always	Touching th	1		Yes	No	0	
Never	Always	Hard when	1		Yes	No	0	
Never	Always	Hard when	1		Yes	No	0	
Never	Always	Touching th	1		Yes	No	0	
Never	Always	Touching th	1		Yes	No	0	
Never	Always	Observe ch	1		Yes	No	0	
Never	Always	Sound reco	1	N/A	Yes	No	0	
Never	Frequent	During mill	1		Yes	No	0	
Never	Frequent	Sound reco	1		Yes	No	0	Good
Never	Frequent	Sound reco	1		Yes	No	0	
Never	Frequent	Sound reco	1		Yes	No	0	
Never	Frequent	Sound reco	1		Yes	No	0	
Never	Frequent	Hard when	1		Yes	No	0	
Never	Always	Hard when	1		Yes	No	0	
Never	Always	Hard when	1		Yes	No	0	
Never	Always	Hard when	1		Yes	No	0	
Never	Always	Hard when	1		Yes	No	0	
One month	Always	Hard when	1		Yes	No	0	
Never	Always	Hard when	1		Yes	No	0	Good
Never	Always	Hard when	1		Yes	No	0	
One week	Always	Hard when	1		Yes	No	0	
Never	Always	Hard when	1		Yes	No	0	
One month	Always	Sound reco	1		Yes	No	0	
One week	One Month	Hard when	1		Yes	No	0	
One week	Always	Hard when	1		Yes	No	0	
One month	Always	Sound reco	1		Yes	No	0	
Never	Frequent	Hard when	1		Yes	No	0	
Never	Frequent	Hard when	1		Yes	No	0	
Never	Frequent	Hard when	1		Yes	No	0	
Never	Frequent	Sound reco	1		Yes	No	0	
Frequent	Frequent	Hard when	1		Yes	No	0	
Never	Frequent	Hard when	1		Yes	No	0	
Never	Frequent	Sound reco	1		Yes	No	0	
Never	Frequent	During shel	1		Yes	No	0	
Never	Frequent	During mill	1		Yes	No	0	
Never	Always	Touching th	1		No	No	0	
One month	Always	Hard when	0	Poor sun h	Yes	No	0	
One month	Always	Touching th	0	Short time	Yes	No	0	
One month	Always	Self shelling	0	Poor sun h	Yes	No	0	
Never	Frequent	Sound reco	1		Yes	No	0	
Never	Frequent	Hard when	1		Yes	No	0	
Never	Frequent	Hard when	1		Yes	No	0	
Never	Frequent	Sound reco	1		Yes	No	0	
Never	Frequent	Sound reco	1		Yes	No	0	

Never	Always	Sound reco	1		Yes	No	0
Never	Always	Hard when	1		Yes	No	0
Never	Frequent	Sound reco	1		Yes	No	0
Never	Frequent	Observe ch	1		Yes	No	0
Never	Always	Sound reco	1		Yes	No	0
Never	Frequent	Hard when	1		Yes	No	0
One month	Always	Self shelling	1		Yes	No	0
Frequent	Frequent	Touching th	1		Yes	No	0
Frequent	Frequent	Hard when	1		No	No	0
One week	One Month	Hard when	1	Poor sun h	Yes	No	0
Frequent	Frequent	During shel	1		Yes	No	0
Never	Always	Touching th	1		Yes	No	0
Never	Always	Touching th	1		Yes	No	0
Never	Always	Touching th	1		Yes	No	0
Never	Always	Hard when	1		Yes	No	0
Never	Always	Hard when	1		Yes	No	0
Never	Always	Touching th	1		Yes	No	0
Never	Always	Sound reco	1		Yes	No	0
Never	Always	Sound reco	1		Yes	No	0
Never	Always	Touching th	1		Yes	No	0
Never	Always	Observe ch	1		Yes	No	0
Never	Always	Sound reco	1		Yes	No	0
Never	Always	Observe ch	1		Yes	No	0
Never	Always	During shel	1		Yes	No	0
Never	Frequent	Self shelling	1		Yes	No	0
Never	Frequent	Self shelling	1		Yes	No	0
Never	Frequent	Self shelling	1		Yes	No	0
Never	Always	Self shelling	1		Yes	No	0
Never	Always	Touching th	1		Yes	No	0
Never	Frequent	Self shelling	1		Yes	No	0
Never	Frequent	Hard when	1		Yes	No	0
Never	Always	Self shelling	1		Yes	No	0
Never	Always	Touching th	1		Yes	No	0
Never	Frequent	Hard when	1		Yes	No	0
Never	Frequent	Hard when	1		Yes	No	0
Never	Frequent	Hard when	1		Yes	No	0
Never	Frequent	Hard when	1		Yes	No	0
Never	Frequent	Sound reco	1		Yes	No	0
Never	Frequent	Sound reco	1		Yes	No	0
Never	Frequent	Sound reco	1		Yes	No	0
Never	Frequent	Sound reco	1		Yes	No	0
Never	Frequent	Hard when	1		Yes	No	0
Never	Frequent	Sound reco	1		Yes	No	0
Never	Frequent	Sound reco	1		Yes	No	0
Never	Frequent	Sound reco	1		Yes	No	0
Never	Frequent	Sound reco	1		Yes	No	0
Never	Frequent	Sound reco	1		Yes	No	0

Never	Frequent	Sound reco	1	Yes	No	0
Never	Frequent	Hard when	1	Yes	No	0
Never	Always	Sound reco	1	Yes	No	0
Never	Always	Hard when	1	Yes	No	0
Never	Always	Sound reco	1	Yes	No	0
Never	Always	Sound reco	1	Yes	No	0
Never	Frequent	Sound reco	1	Yes	No	0
Never	Frequent	Sound reco	1	Yes	No	0
Never	Frequent	Hard when	1	Yes	No	0
Never	Frequent	Sound reco	1	Yes	No	0
Never	Frequent	Sound reco	1	Yes	No	0
Never	Frequent	Hard when	1	Yes	No	0
Never	Frequent	Sound reco	1	Yes	No	0
Never	Frequent	Sound reco	1	Yes	No	0
Never	Always	Hard when	1	Yes	No	0
Never	Frequent	After sun d	1	Yes	No	0
Never	Frequent	Hard when	1	Yes	No	0
One montl	Always	Self shellin	1	No	No	0
One montl	Always	Self shellin	1	Yes	No	0
One week	One week	Hard when	1	Yes	No	0
One montl	Always	Sound reco	1	Yes	No	0
Never	Always	Hard when	1	Yes	No	0
Never	Always	Hard when	1	Yes	No	0
Never	Always	Hard when	1	Yes	No	0
One montl	Always	Hard when	1	Yes	No	0
One montl	Always	Hard when	1	Yes	No	0
Never	Always	Hard when	1	Yes	No	0
Frequent	Frequent	Touching tl	1	Yes	No	0
Frequent	Frequent	Touching tl	1	Yes	No	0
Frequent	Frequent	Touching tl	1	No	No	0
Frequent	Frequent	Sound reco	1	Yes	No	0
Frequent	Frequent	Touching tl	1	Yes	No	0
Frequent	Frequent	Sound reco	1	No	No	0
Never	Always	Touching tl	1	Yes	No	0
Never	Always	Hard when	1	Yes	No	0
Never	Frequent	Observe ch	1	Yes	No	0
Never	Frequent	Self shellin	1	Yes	No	0
Never	Always	Sound reco	1	Yes	No	0
Never	Always	Sound reco	1	Yes	No	0
Never	Always	Sound reco	1	Yes	No	0
Never	Always	Observe ch	1	Yes	No	0
Never	Frequent	Observe ch	1	Yes	No	0
Never	Frequent	Sound reco	1	Yes	No	0
Never	Frequent	Touching tl	1	Yes	No	0
Never	Frequent	Sound reco	1	Yes	No	0
Never	Always	Sound reco	1	Yes	No	0
Never	Frequent	Sound reco	1	Yes	No	0

Never	Frequent	Sound reco	1		Yes	No	0	
Never	Frequent	Sound reco	1		Yes	No	0	
Never	Frequent	Sound reco	1		Yes	No	0	
Never	Frequent	Sound and	1		Yes	No	0	
Never	Frequent	Salting grai	1		Yes	No	0	
Never	Frequent	Sound reco	1		Yes	No	0	
Never	Frequent	Hard when	1		Yes	No	0	
Never	Frequent	Sound reco	1		Yes	No	0	
Never	Frequent	Sound reco	1		Yes	No	0	
Never	Frequent	Sound reco	1		Yes	No	0	
Never	Frequent	Self shelling	1		Yes	No	0	
Never	Frequent	Hard when	1		Yes	No	0	
Never	Frequent	Sound reco	1		Yes	No	0	
Never	Frequent	Sound reco	1		Yes	No	0	
Never	Always	Sound reco	1		Yes	No	0	
Never	Always	During shel	1		Yes	No	0	
Never	Frequent	Hard when	1		Yes	No	0	
Never	Always	Sound reco	1		Yes	No	0	
Never	Always	Hard when	1		Yes	No	0	
Never	Always	Hard when	1		Yes	No	0	
Never	Always	Hard when	1		Yes	No	0	
Never	Always	Self shelling	1		Yes	No	0	
Never	Always	Touching th	1		Yes	No	0	
Never	Always	After sun d	1		Yes	No	0	
Never	Always	During shel	1		Yes	No	0	
Never	Always	Self shelling	1		Yes	No	0	
Never	Always	Sound reco	1		Yes	No	0	
Never	Always	Sound reco	1		Yes	No	0	
Never	Always	Sound reco	1		Yes	Yes	1260	Good
Never	Always	Touching th	1	N/A	Yes	Yes	12600	Good
Never	Always	Hard when	1	N/A	Yes	Yes	1350	Good
Never	Always	Hard when	1	N/A	Yes	Yes	1350	Good
Never	Always	Touching th	1	N/A	Yes	Yes	1350	Good
Never	Always	Touching th	1	N/A	Yes	Yes	1350	Good
Never	Never	Sound reco	1		Yes	Yes	1350	Good
Never	Frequent	Sound reco	1		Yes	Yes	1350	Good
Never	Frequent	Sound reco	1		Yes	Yes	1350	Good
Never	Frequent	Sound reco	1		Yes	Yes	1350	Good
Never	Frequent	Sound reco	1		Yes	Yes	1350	Good
Never	Frequent	Hard when	1		Yes	Yes	1350	Good
Never	Always	Hard when	1		Yes	Yes	180	Good
Never	Always	Hard when	1	N/A	Yes	Yes	180	Good
Never	Always	Hard when	1		Yes	Yes	180	Good
Never	Frequent	Hard when	1		Yes	Yes	180	Good
Frequent	Frequent	Hard when	1		Yes	Yes	180	
Never	Always	Self shelling	1		Yes	Yes	180	Good
Never	Frequent	Hard when	1		Yes	Yes	1800	Good

Frequent	Frequent	Hard when	1		Yes	Yes	1800	Good
Never	Always	Touching tl	1	N/A	Yes	Yes	1800	Good
Never	Always	Hard when	1	N/A	Yes	Yes	1800	Good
Never	Frequent	Sound reco	1		Yes	Yes	1800	Good
Never	Frequent	Sound reco	1		Yes	Yes	1800	Good
Never	Frequent	Sound reco	1		Yes	Yes	1800	
One week	Always	Sound reco	1		Yes	Yes	1800	Fair
Never	Always	Hard when	1		Yes	Yes	1980	Good
Never	Always	Hard when	0	Poor sun h	Yes	Yes	2250	Good
Never	Frequent	Sound reco	1		Yes	Yes	2250	Good
Always	Frequent	Sound reco	1		Yes	Yes	270	Good
Never	Always	Touching tl	1		Yes	Yes	270	Good
Never	Always	Hard when	1	N/A	Yes	Yes	270	Good
Never	Frequent	Sound reco	1		Yes	Yes	270	Good
Never	Always	Hard when	1		Yes	Yes	270	Good
One month	Always	Hard when	1		Yes	Yes	270	Good
Never	Frequent	Sound reco	1		Yes	Yes	270	Good
Never	Frequent	Hard when	1		Yes	Yes	270	Good
Never	Frequent	Hard when	1		Yes	Yes	270	Good
Never	Always	Sound reco	1		Yes	Yes	270	Good
Frequent	Frequent	Hard when	1		Yes	Yes	2700	Good
Never	Frequent	Sound reco	1		Yes	Yes	2700	Good
Never	Always	Touching tl	1	N/A	Yes	Yes	2700	Good
Never	Frequent	Observe ch	1		Yes	Yes	2700	Good
Never	Always	Hard when	1	N/A	Yes	Yes	2700	Good
One week	Always	Hard when	1		Yes	Yes	2700	Good
One month	Always	Sound reco	1		Yes	Yes	3060	Poor
Never	Frequent	Hard when	1		Yes	Yes	3060	Good
Never	Always	During shel	1		Yes	Yes	34	Good
Never	Always	Touching tl	1		Yes	Yes	360	Good
Never	Always	Touching tl	1		Yes	Yes	360	Good
Never	Frequent	Hard when	1		Yes	Yes	360	Good
Never	Frequent	Sound reco	1		Yes	Yes	360	Good
Never	Always	Sound reco	1		Yes	Yes	360	
Never	Always	Touching tl	1	N/A	Yes	Yes	3600	Good
Never	Never	Sound reco	1		Yes	Yes	3600	Good
One week	Always	Hard when	1		Yes	Yes	3600	Good
Never	Never	Sound reco	1		Yes	Yes	450	Good
Never	Never	Sound reco	1		Yes	Yes	450	Good
Never	Frequent	Observe ch	1		Yes	Yes	450	Good
Frequent	Frequent	Hard when	1		Yes	Yes	450	Good
Never	Always	Hard when	1	N/A	Yes	Yes	450	Good
Never	Always	Touching tl	1	N/A	Yes	Yes	450	Good
Never	Always	Touching tl	1	N/A	Yes	Yes	450	Good
Never	Frequent	Sound reco	1		Yes	Yes	450	Good
Never	Frequent	Hard when	1		Yes	Yes	450	Good
Never	Frequent	Sound reco	1		Yes	Yes	450	Good

Never	Frequent	Hard when	1		Yes	Yes	450	Good
Never	Always	Hard when	1		Yes	Yes	450	Poor
One week	Always	Hard when	1		Yes	Yes	450	Good
Never	Always	Sound reco	1		Yes	Yes	450	Good
Never	Always	Hard when	1	N/A	Yes	Yes	540	Good
Never	Frequent	Sound reco	1		Yes	Yes	540	Good
Never	Always	Hard when	1		Yes	Yes	6	Good
Always	Always	Hard when	1		Yes	Yes	630	Good
Never	Always	Hard when	1	N/A	Yes	Yes	630	Good
Always	Always	Hard when	1		Yes	Yes	630	Good
Always	Always	Hard when	1		Yes	Yes	630	Good
Never	Frequent	Sound reco	1		Yes	Yes	720	Good
Never	Frequent	Sound reco	1		Yes	Yes	720	Good
Never	Frequent	Sound reco	0	Seeds was	Yes	Yes	90	Good
One week	Always	Hard when	1		Yes	Yes	90	Poor
Never	Always	Touching th	1		Yes	Yes	90	Good
Never	Frequent	Sound reco	1		Yes	Yes	90	Good
Never	Always	Observe ch	1		Yes	Yes	90	
Never	Frequent	Sound reco	1		Yes	Yes	90	
Never	Frequent	Self shellin	1		Yes	Yes	90	Good
Never	Always	Hard when	1		Yes	Yes	90	Good
Never	Frequent	Observe ch	0	Poor sprea	Yes	Yes	900	Good
Never	Frequent	Observe ch	1		Yes	Yes	900	Good
Never	Frequent	Hard when	1		Yes	Yes	900	Good
Never	Always	Observe ch	1	N/A	Yes	Yes	900	Good
Frequent	Frequent	Touching th	1	N/A	Yes	Yes	900	Good
Frequent	Always	Hard when	1	N/A	Yes	Yes	900	Good
Frequent	Frequent	Touching th	1	N/A	Yes	Yes	900	Good
Never	Always	Touching th	1	N/A	Yes	Yes	900	Good
Never	Always	Hard when	1		Yes	Yes	900	Good
Never	Always	Hard when	1		Yes	Yes	900	Good
Frequent	Frequent	Hard when	1		Yes	Yes	900	Good
Frequent	Frequent	Observe ch	1		Yes	Yes	900	Good

avarage_pr	buyer_larg	much_disc	poor_soil	wet_weath	wetness_pi	dampness	earlier_har	pests_stora
			Yes	Yes	Yes	Yes	Yes	Yes
			Yes	Yes	Yes	Yes	Yes	Yes
			No	Yes	No	Yes	No	No
			No	Yes	Yes	Yes	Yes	No
			No	Yes	No	Yes	Yes	Yes
			No	Yes	Yes	Yes	No	No
			Yes	Yes	Yes	Yes	Yes	Yes
			Yes	Yes	Yes	Yes	Yes	Yes
			Yes	Yes	Yes	Yes	Yes	Yes
			No	Yes	Yes	Yes	Yes	No
			Yes	Yes	Yes	Yes	Yes	Yes
			No	Yes	Yes	Yes	Yes	Yes
			No	Yes	Yes	Yes	No	No
			No	Yes	Yes	Yes	No	Yes
			No	Yes	No	Yes	Yes	Yes
			No	Yes	Yes	No	No	Yes
			No	Yes	Yes	Yes	Yes	Yes
			Yes	Yes	Yes	Yes	No	Yes
			No	Yes	Yes	Yes	Yes	Yes
			No	Yes	Yes	Yes	No	Yes
			Yes	Yes	Yes	Yes	Yes	Yes
			No	Yes	Yes	Yes	Yes	Yes
			No	Yes	Yes	Yes	Yes	Yes
			Yes	Yes	Yes	Yes	Yes	Yes
			Yes	Yes	Yes	Yes	Yes	Yes
			No	Yes	Yes	Yes	Yes	No
	N/A		Yes	Yes	No	Yes	Yes	Yes
			No	Yes	Yes	Yes	No	No
			No	Yes	Yes	Yes	No	No
			No	Yes	Yes	Yes	Yes	No
			No	Yes	Yes	Yes	Yes	No
			Yes	Yes	Yes	Yes	Yes	Yes
			Yes	Yes	Yes	Yes	Yes	Yes
			No	Yes	Yes	Yes	Yes	No
			No	Yes	Yes	Yes	Yes	Yes
			No	Yes	Yes	Yes	Yes	Yes
			Yes	Yes	Yes	Yes	Yes	Yes
			Yes	Yes	Yes	Yes	Yes	Yes
			Yes	Yes	No	Yes	No	No
			Yes	Yes	Yes	Yes	Yes	Yes
			Yes	Yes	Yes	Yes	Yes	Yes
			Yes	Yes	Yes	Yes	Yes	Yes
			No	Yes	Yes	Yes	Yes	Yes
			Yes	Yes	Yes	Yes	Yes	Yes
			Yes	Yes	Yes	Yes	Yes	Yes
			Yes	Yes	Yes	Yes	No	Yes

No	Yes	Yes	Yes	Yes	Yes
Yes	No	Yes	Yes	Yes	No
No	Yes	Yes	Yes	Yes	No
No	Yes	Yes	Yes	Yes	Yes
No	Yes	Yes	Yes	Yes	Yes
No	Yes	Yes	Yes	Yes	Yes
No	Yes	Yes	Yes	Yes	Yes
Yes	Yes	Yes	Yes	Yes	Yes
No	Yes	Yes	Yes	No	Yes
No	Yes	Yes	Yes	Yes	Yes
Yes	Yes	Yes	Yes	Yes	No
No	Yes	Yes	Yes	Yes	No
No	Yes	Yes	Yes	Yes	No
No	Yes	Yes	Yes	Yes	No
No	Yes	Yes	Yes	Yes	No
No	Yes	Yes	Yes	Yes	No
Yes	Yes	Yes	Yes	Yes	Yes
Yes	Yes	Yes	Yes	Yes	Yes
Yes	Yes	Yes	Yes	Yes	Yes
Yes	Yes	Yes	Yes	Yes	Yes
No	Yes	Yes	Yes	Yes	No
Yes	Yes	Yes	Yes	Yes	Yes
Yes	Yes	Yes	Yes	Yes	Yes
No	Yes	Yes	Yes	Yes	No
No	Yes	Yes	Yes	Yes	Yes
Yes	Yes	Yes	Yes	Yes	Yes
Yes	Yes	Yes	Yes	Yes	Yes
Yes	Yes	Yes	Yes	Yes	Yes
Yes	Yes	Yes	Yes	Yes	Yes
No	Yes	Yes	Yes	Yes	Yes
No	Yes	Yes	Yes	Yes	Yes
No	Yes	Yes	Yes	Yes	No
No	Yes	Yes	Yes	Yes	No
Yes	Yes	Yes	Yes	Yes	No
No	Yes	Yes	Yes	Yes	Yes
Yes	Yes	Yes	Yes	Yes	No
No	Yes	Yes	Yes	Yes	No
No	Yes	Yes	Yes	Yes	No
Yes	Yes	Yes	Yes	Yes	Yes
No	Yes	Yes	Yes	Yes	Yes
No	Yes	Yes	Yes	Yes	No
No	Yes	Yes	Yes	Yes	Yes
No	Yes	Yes	Yes	Yes	No
No	Yes	Yes	Yes	Yes	No
No	Yes	Yes	Yes	Yes	No
No	No	No	No	No	Yes
No	Yes	Yes	Yes	Yes	Yes

No	Yes	Yes	Yes	Yes	No
No	Yes	No	Yes	Yes	No
No	No	No	No	No	No
Yes	Yes	Yes	Yes	Yes	Yes
No	Yes	Yes	Yes	No	No
Yes	Yes	Yes	Yes	Yes	No
No	Yes	Yes	Yes	Yes	Yes
No	Yes	Yes	Yes	Yes	No
No	Yes	Yes	Yes	Yes	No
No	Yes	Yes	Yes	Yes	Yes
No	Yes	Yes	Yes	Yes	No
No	Yes	Yes	Yes	Yes	Yes
No	Yes	Yes	Yes	Yes	Yes
No	Yes	Yes	Yes	Yes	Yes
No	Yes	Yes	Yes	Yes	Yes
No	Yes	Yes	Yes	Yes	Yes
No	Yes	Yes	Yes	Yes	Yes
No	Yes	Yes	Yes	Yes	Yes
No	Yes	Yes	Yes	Yes	Yes
No	Yes	Yes	Yes	Yes	No
Yes	No	Yes	Yes	Yes	Yes
No	Yes	Yes	Yes	Yes	Yes
No	Yes	Yes	Yes	Yes	Yes
Yes	Yes	Yes	No	Yes	No
Yes	Yes	Yes	Yes	Yes	No
No	Yes	Yes	Yes	Yes	No
Yes	Yes	Yes	Yes	Yes	Yes
Yes	No	Yes	No	No	No
Yes	Yes	Yes	Yes	Yes	No
Yes	Yes	Yes	Yes	Yes	No
No	Yes	Yes	Yes	Yes	No
No	Yes	Yes	Yes	Yes	No
Yes	Yes	Yes	Yes	Yes	Yes
No	Yes	Yes	Yes	Yes	No
No	Yes	Yes	Yes	Yes	No
No	Yes	Yes	Yes	Yes	No
No	Yes	Yes	Yes	Yes	No
Yes	No	Yes	No	Yes	No
No	No	No	No	No	No
No	No	No	No	No	No
No	No	No	No	No	No
No	No	No	No	No	No
No	Yes	Yes	No	No	No
No	No	No	No	No	No
No	Yes	No	No	No	No
No	No	No	No	No	No
No	No	No	No	No	No

No	Yes	Yes	Yes	No	Yes
No	Yes	Yes	Yes	Yes	No
No	Yes	Yes	Yes	Yes	No
No	Yes	Yes	Yes	Yes	No
No	Yes	Yes	Yes	Yes	Yes
No	Yes	Yes	Yes	Yes	No
No	Yes	Yes	Yes	No	No
Yes	No	Yes	Yes	No	No
No	No	No	Yes	No	No
No	Yes	Yes	Yes	No	Yes
No	Yes	Yes	Yes	Yes	Yes
No	Yes	Yes	Yes	Yes	Yes
No	Yes	Yes	Yes	No	No
No	No	No	No	No	No
Yes	Yes	Yes	Yes	Yes	Yes
No	No	No	No	No	No
No	Yes	Yes	Yes	Yes	No
No	Yes	Yes	Yes	Yes	Yes
Yes	Yes	Yes	Yes	Yes	Yes
No	Yes	Yes	Yes	Yes	Yes
No	Yes	Yes	Yes	Yes	Yes
No	Yes	Yes	Yes	Yes	Yes
No	Yes	Yes	Yes	Yes	Yes
No	Yes	Yes	Yes	Yes	Yes
No	Yes	Yes	Yes	Yes	Yes
No	Yes	Yes	Yes	Yes	Yes
No	Yes	Yes	Yes	Yes	Yes
No	Yes	Yes	Yes	Yes	No
No	Yes	Yes	Yes	Yes	No
No	Yes	Yes	Yes	Yes	No
No	Yes	Yes	Yes	Yes	Yes
No	Yes	Yes	Yes	Yes	No
No	Yes	Yes	Yes	Yes	No
Yes	Yes	Yes	Yes	Yes	Yes
Yes	Yes	Yes	Yes	Yes	Yes
No	Yes	Yes	Yes	Yes	No
No	Yes	Yes	Yes	Yes	No
No	Yes	Yes	Yes	Yes	No
No	No	No	No	No	No
No	Yes	Yes	Yes	Yes	Yes
No	Yes	Yes	Yes	No	Yes
No	Yes	Yes	Yes	Yes	Yes
No	Yes	Yes	Yes	Yes	No
Yes	Yes	Yes	Yes	Yes	No
No	Yes	Yes	Yes	Yes	No
Yes	Yes	Yes	Yes	Yes	No
No	Yes	Yes	Yes	Yes	No

			Yes	Yes	Yes	Yes	No	No
			Yes	Yes	Yes	Yes	Yes	Yes
			No	No	No	Yes	Yes	Yes
			No	Yes	Yes	Yes	Yes	No
			Yes	Yes	Yes	Yes	Yes	No
			Yes	Yes	Yes	Yes	Yes	No
			Yes	Yes	Yes	Yes	Yes	No
			Yes	Yes	No	No	No	No
			Yes	Yes	Yes	Yes	Yes	Yes
			Yes	Yes	Yes	Yes	Yes	No
			Yes	Yes	Yes	Yes	Yes	No
			Yes	Yes	No	No	No	No
			Yes	Yes	Yes	Yes	Yes	No
			No	Yes	Yes	Yes	Yes	No
			No	No	No	Yes	No	No
			No	No	No	Yes	Yes	No
			No	Yes	Yes	Yes	Yes	Yes
			No	Yes	Yes	Yes	Yes	No
			No	Yes	Yes	Yes	Yes	No
			No	Yes	Yes	Yes	Yes	Yes
			Yes	Yes	Yes	Yes	Yes	No
			No	No	Yes	Yes	Yes	Yes
			No	No	No	No	Yes	No
			No	Yes	Yes	Yes	Yes	Yes
			No	No	Yes	Yes	Yes	Yes
			Yes	Yes	Yes	Yes	Yes	No
			No	Yes	Yes	Yes	No	No
			No	Yes	Yes	Yes	No	No
4500	Business man		No	Yes	Yes	Yes	Yes	No
3000	Business m	45	No	Yes	Yes	Yes	Yes	Yes
2000	Local Consumers		No	No	No	No	No	No
1400	Cereal		No	No	Yes	Yes	Yes	Yes
2300	Cereal		No	Yes	Yes	Yes	Yes	Yes
2000	Cereal		No	Yes	Yes	Yes	Yes	Yes
3000	Business man		No	Yes	Yes	Yes	Yes	No
2500	Business man		Yes	Yes	Yes	Yes	No	No
2500	Business man		No	Yes	Yes	Yes	Yes	No
2200	Business man		No	Yes	Yes	Yes	Yes	No
2200	Business man		No	Yes	Yes	Yes	Yes	No
3800	Business man		No	Yes	Yes	Yes	Yes	No
4000	School		Yes	Yes	Yes	Yes	Yes	Yes
2200	Business m	2	Yes	Yes	Yes	Yes	Yes	Yes
4000	Business m	23	No	Yes	Yes	Yes	Yes	Yes
2300	Business man		No	Yes	Yes	Yes	Yes	No
2500	Local Consumers		No	Yes	Yes	Yes	Yes	No
3600	Business man		Yes	Yes	Yes	Yes	Yes	Yes
2500	Business man		Yes	Yes	Yes	Yes	Yes	Yes

2700	Business m	2	No	Yes	Yes	Yes	No	No
2100	Local Consumers		No	No	Yes	Yes	Yes	Yes
2400	Business m	2	Yes	Yes	Yes	Yes	Yes	Yes
2000	Business man		No	Yes	Yes	Yes	Yes	No
2500	Business man		No	Yes	Yes	Yes	Yes	No
2000	Business man		No	Yes	Yes	Yes	Yes	No
2500	Business man		No	Yes	Yes	Yes	Yes	Yes
3000	Business m	180	No	No	Yes	No	Yes	No
1660	Cereal	23	Yes	Yes	Yes	Yes	Yes	Yes
2000	Business man		No	Yes	Yes	Yes	Yes	No
2500	Business man		No	Yes	Yes	No	No	No
4000	School		No	Yes	Yes	Yes	Yes	Yes
2300	Business man		No	Yes	Yes	Yes	Yes	Yes
6600	Business man		No	Yes	Yes	Yes	Yes	No
1700	Business man		Yes	Yes	Yes	Yes	Yes	Yes
1600	Business man		No	Yes	Yes	Yes	Yes	No
2700	Business man		Yes	Yes	Yes	Yes	Yes	No
3000	Business man		No	Yes	Yes	Yes	Yes	No
7500	Business man		Yes	Yes	Yes	Yes	Yes	Yes
6700	Business man		No	Yes	Yes	Yes	No	No
3600	Business m	22	No	Yes	Yes	Yes	No	No
2500	Local Consumers		Yes	Yes	Yes	Yes	Yes	Yes
2350	Local Consumers		Yes	Yes	Yes	Yes	No	Yes
2500	Business man		Yes	Yes	Yes	Yes	Yes	Yes
2000	Business man		No	Yes	Yes	Yes	Yes	Yes
1800	Business man		Yes	Yes	Yes	Yes	Yes	Yes
3000	Business man		No	Yes	Yes	Yes	Yes	No
2000	Business m	23	No	Yes	Yes	Yes	Yes	No
2000	Business man		Yes	Yes	Yes	Yes	Yes	Yes
4000	School		No	Yes	Yes	Yes	Yes	Yes
4000	Business man		Yes	Yes	Yes	Yes	Yes	Yes
5000	Business man		No	Yes	Yes	Yes	Yes	No
2300	Business man		No	Yes	Yes	Yes	Yes	No
3500	Business man		No	Yes	Yes	Yes	No	No
5000	Local Consu	23	No	Yes	Yes	Yes	Yes	Yes
2500	Business man		No	Yes	Yes	Yes	Yes	No
2000	Business man		No	Yes	Yes	Yes	Yes	No
2500	Business man		Yes	Yes	Yes	Yes	No	No
2500	Business man		No	No	No	Yes	No	No
3000	Business man		No	No	Yes	Yes	Yes	Yes
2700	Business man		No	Yes	Yes	Yes	No	No
2100	Local Consumers		Yes	Yes	Yes	Yes	Yes	Yes
2100	Cereal		No	Yes	Yes	Yes	Yes	Yes
2200	Business man		No	Yes	Yes	Yes	Yes	Yes
2000	Business man		Yes	Yes	No	Yes	Yes	Yes
2500	Business man		No	No	No	No	No	Yes
2000	Business man		No	Yes	Yes	Yes	Yes	No

2500	Business man		No	Yes	Yes	Yes	Yes	Yes
2500	Cereal		Yes	Yes	Yes	Yes	Yes	Yes
2500	Local Consu	2	No	Yes	Yes	Yes	Yes	Yes
3000	Business man		Yes	Yes	Yes	Yes	Yes	Yes
2000	Local Consumers		No	Yes	Yes	Yes	Yes	Yes
2500	Business man		No	Yes	Yes	Yes	Yes	No
2500	Cereal		Yes	Yes	Yes	Yes	Yes	Yes
2500	Business man		No	Yes	Yes	Yes	No	No
2850	Local Consumers		Yes	Yes	Yes	Yes	Yes	Yes
2600	Business man		No	Yes	Yes	Yes	Yes	Yes
2600	Business man		No	Yes	Yes	Yes	Yes	Yes
3000	Business man		No	Yes	Yes	Yes	Yes	No
1000	Business man		No	Yes	Yes	Yes	Yes	Yes
2000	Business man		No	Yes	Yes	Yes	Yes	No
2000	Business m	12	Yes	Yes	Yes	Yes	Yes	Yes
2700	Business m	23	No	No	No	No	Yes	No
4500	Business man		No	Yes	Yes	Yes	Yes	No
2500	Business man		No	Yes	Yes	Yes	Yes	Yes
2700	Business man		Yes	Yes	Yes	Yes	Yes	No
2700	Local Consumers		No	Yes	Yes	Yes	Yes	No
1000	Local Consumers		Yes	Yes	Yes	Yes	No	Yes
2400	Business man		No	Yes	Yes	Yes	Yes	No
2500	Business man		No	Yes	Yes	Yes	Yes	No
2200	Business man		No	Yes	Yes	Yes	Yes	No
2100	Local Consumers		No	No	Yes	Yes	Yes	Yes
2000	Local Consumers		No	Yes	Yes	Yes	Yes	Yes
2100	Cereal		No	No	Yes	Yes	Yes	Yes
2200	Local Consumers		No	No	Yes	Yes	Yes	Yes
2000	Business man		No	Yes	Yes	Yes	Yes	Yes
2200	Business m	270	Yes	Yes	Yes	Yes	Yes	Yes
2800	Business man		Yes	Yes	Yes	Yes	Yes	Yes
2000	Business man		No	No	No	No	No	No
2300	Business man		Yes	Yes	Yes	Yes	Yes	No

drying_long	spreading_	completely	plastic_bag	plastic_con	metal_silo	clay_pot	source_fan	specify_sou
No	Yes	Yes	No	No	No	No	Salaried job	
No	Yes	Yes	No	No	No	No	Farmer	
No	Yes	Yes	No	No	No	No	Farmer	
No	No	Yes	No	No	No	No	Farmer	
No	No	Yes	No	No	No	No	Farmer	
No	Yes	Yes	No	No	No	No	Farmer	
No	Yes	Yes	Yes	No	No	No	Merchant	
No	Yes	Yes	Yes	No	No	No	Merchant	
No	Yes	Yes	Yes	No	Yes	No	Salaried job	
No	Yes	Yes	No	No	No	No	Merchant	
No	Yes	Yes	No	No	No	No	Farmer	
No	Yes	Yes	Yes	No	Yes	No	Salaried job	
No	Yes	Yes	No	No	No		Farmer	
No	Yes	Yes	Yes	No	No	No	Farmer	
No	Yes	Yes	No	No	No	No	Merchant	
No	Yes	Yes	No	No	No	No	Farmer	
No	Yes	Yes	No	No	No	No	Farmer	
No	No	Yes	No	No	No	No	Farmer	
No	Yes	Yes	No	No	No	No	Farmer	
No	Yes	Yes	No	No	No	No	Farmer	
No	Yes	Yes	No	No	No	No	Farmer	
No	Yes	Yes	No	No	No	No	Farmer	
No	Yes	Yes	No	No	No	No	Farmer	
No	Yes	Yes	Yes	No	Yes	No	Salaried job	
No	Yes	Yes	No	No	No	No	Salaried job	
No	Yes	Yes	No	No	Yes	No	Farmer	
No	Yes	Yes	No	No	Yes	Yes	Farmer	
No	No	Yes	No	No	Yes	Yes	Farmer	
No	No	Yes	No	No	No	No	Farmer	
No	Yes	Yes	No	No	No	No	Farmer	
No	No	Yes	No	No	No	No	Farmer	
Yes	Yes	Yes	No	No	No	Yes	Farmer	
Yes	Yes	Yes	No	No	No	Yes	Farmer	
No	No	Yes	No	No	No	No	Farmer	
No	Yes	Yes	No	No	No	Yes	Others	Motorcylist
No	Yes	Yes	No	No	No	No	Farmer	
No	Yes	Yes	No	No	No	No	Farmer	
No	Yes	Yes	Yes	No	No	No	Salaried job	
No	No	Yes	No	No	No	No	Farmer	
No	Yes	Yes	Yes	No	No	No	Merchant	
No	Yes	Yes	Yes	No	No	No	Salaried job	
No	Yes	Yes	Yes	No	No	No	Salaried job	
No	Yes	Yes	No	No	No	No	Merchant	
No	Yes	Yes	Yes	No	Yes	No	Salaried job	
No	Yes	Yes	No	No	Yes	No	Salaried job	
No	Yes	Yes	No	No	Yes	No	Salaried job	

Yes	Yes	Yes	Yes	No	No	No	Farmer	
No	Yes	Yes	No	No	No	No	Farmer	
Yes	Yes	Yes	Yes	No	No	No	Farmer	
No	Yes	Yes	No	No	No	No	Salaried job	
No	Yes	Yes	No	No	No	No	Farmer	
No	Yes	Yes	No	No	No	No	Salaried job	
No	Yes	Yes	No	No	No	No	Merchant	
No	Yes	Yes	No	No	No	No	Farmer	
No	Yes	Yes	No	No	Yes	No	Others	
No	Yes	Yes	Yes	Yes	Yes	Yes	Farmer	
No	Yes	Yes	No	No	Yes	Yes	Farmer	
No	No	Yes	No	No	No	No	Salaried job	
No	No	Yes	No	No	No	No	Farmer	
No	No	Yes	No	No	No	No	Farmer	
No	No	Yes	No	No	No	No	Farmer	
No	No	Yes	No	No	No	No	Merchant	
Yes	Yes	No	No	No	No	No	Farmer	
Yes	Yes	Yes	No	No	No	Yes	Farmer	
Yes	Yes	Yes	No	No	No	Yes	Farmer	
Yes	Yes	Yes	No	No	No	Yes	Farmer	
No	No	Yes	No	No	No	Yes	Farmer	
Yes	Yes	Yes	No	No	No	Yes	Farmer	
Yes	No	Yes	No	No	No	Yes	Farmer	
No	No	Yes	No	No	No	No	Farmer	
No	Yes	Yes	No	No	Yes	Yes	Salaried job	
No	Yes	Yes	No	No	Yes	No	Farmer	
No	Yes	Yes	Yes	No	No	No	Salaried job	
No	Yes	Yes	Yes	No	No	No	Farmer	
No	Yes	Yes	No	No	Yes	No	Farmer	
No	Yes	Yes	No	No	Yes	Yes	Salaried job	
No	Yes	Yes	No	No	Yes	Yes	Farmer	
Yes	No	No	No	No	No	No	Farmer	
No	No	Yes	No	No	No	No	Others	Dependant
No	Yes	No	No	No	No	No	Others	
No	No	Yes	No	No	No	No	Salaried job	
No	No	Yes	No	No	No	No	Farmer	
No	No	Yes	No	No	No	No	Farmer	
No	Yes	Yes	No	No	No	No	Salaried job	
Yes	Yes	Yes	Yes	Yes	Yes	Yes	Farmer	
No	No	Yes	Yes	Yes	Yes	Yes	Farmer	
No	Yes	Yes	No	No	No	No	Farmer	
No	Yes	Yes	Yes	Yes	Yes	Yes	Farmer	
Yes	Yes	Yes	Yes	No	No	Yes	Farmer	
No	No	Yes	No	No	No	No	Farmer	
No	No	Yes	No	No	No	No	Farmer	
No	No	Yes	No	No	No	No	Farmer	
No	No	No	No	No	No	No	Others	

No	Yes	Yes	No	No	No	No	Farmer	Surport by
No	No	Yes	No	No	No	No	Farmer	
No	Yes	Yes	Yes	Yes	Yes	No	Farmer	
No	Yes	Yes	Yes	Yes	Yes	No	Farmer	
No	No	Yes	No	No	No	No	Farmer	
No	No	Yes	No	No	No	No	Others	Business m
No	Yes	Yes	Yes	Yes	Yes	Yes	Farmer	
No	No	Yes	Yes	Yes	Yes	Yes	Farmer	
No	No	Yes	No	No	No	Yes	Farmer	
Yes	No	Yes	Yes	Yes	Yes	Yes	Farmer	
No	Yes	Yes	No	No	No	Yes	Farmer	
No	Yes	Yes	No	No	No	No	Salaried job	
Yes	Yes	Yes	Yes	Yes	Yes	Yes	Farmer	
Yes	Yes	Yes	Yes	Yes	Yes	Yes	Farmer	
Yes	Yes	Yes	Yes	Yes	No	Yes	Farmer	
Yes	Yes	Yes	Yes	No	Yes	Yes	Farmer	
Yes	Yes	Yes	Yes	Yes	Yes	Yes	Farmer	
Yes	No	Yes	No	No	Yes	Yes	Farmer	
No	No	Yes	No	No	Yes	Yes	Farmer	
No	No	No	No	No	Yes	Yes	Salaried job	
No	Yes	Yes	No	No	No	Yes	Farmer	
No	No	Yes	No	No	Yes	Yes	Farmer	
No	No	Yes	No	No	Yes	Yes	Farmer	
No	No	Yes	No	No	No	No	Farmer	
No	Yes	Yes	Yes	No	No	Yes	Others	Casual labc
No	No	Yes	No	No	No	No	Farmer	
Yes	Yes	Yes	No	No	No	No	Farmer	
No	Yes	Yes	No	No	No	No	Salaried job	
Yes	No	Yes	No	No	Yes	No	Farmer	
No	No	Yes	Yes	Yes	No	No	Farmer	
No	Yes	Yes	No	No	No	Yes	Farmer	
No	No	Yes	No	No	No	No	Farmer	
No	Yes	Yes	Yes	Yes	No	No	Salaried job	
No	Yes	Yes	Yes	No	Yes	No	Farmer	
No	No	Yes	Yes	No	No	Yes	Farmer	
No	Yes	Yes	Yes	No	No	Yes	Farmer	
No	Yes	Yes	Yes	No	No	Yes	Farmer	
No	No	Yes	No	No	No	No	Salaried job	
No	No	No	No	No	No	No	Farmer	
Yes	No	Yes	No	No	No	No	Farmer	
No	No	Yes	No	No	Yes	No	Farmer	
Yes	No	No	No	No	No	No	Farmer	
No	No	Yes	No	No	No	No	Farmer	
No	No	Yes	No	No	No	No	Farmer	
Yes	No	Yes	No	No	No	No	Farmer	
No	No	Yes	No	No	No	No	Farmer	
No	No	No	No	No	No	No	Farmer	

No	Yes	Yes	Yes	No	Yes	No	Farmer	
No	Yes	Yes	Yes	No	No	No	Farmer	
No	Yes	Yes	Yes	Yes	No	No	Farmer	
No	Yes	Yes	Yes	Yes	No	No	Farmer	
No	Yes	Yes	Yes	No	No	No	Farmer	
No	Yes	Yes	Yes	Yes	No	No	Farmer	
No	Yes	Yes	Yes	No	No	Yes	Farmer	
No	Yes	No	Yes	Yes	Yes	No	Farmer	
No	Yes	Yes	No	No	No	No	Farmer	
No	No	Yes	Yes	Yes	No	No	Farmer	
No	Yes	Yes	No	No	No	No	Salaried job	
No	No	No	No	No	No	No	Farmer	
No	No	Yes	No	Yes	No	No	Pension	
No	No	Yes	No	No	No	No	Farmer	
Yes	Yes	Yes	No	No	No	No	Farmer	Salaried jol
Yes	No	Yes	No	No	No	No	Farmer	
No	No	Yes	No	No	No	No	Farmer	
No	Yes	Yes	Yes	Yes	Yes	Yes	Farmer	
No	Yes	Yes	No	No	Yes	Yes	Farmer	
No	No	Yes	Yes	Yes	Yes	Yes	Farmer	
Yes	No	Yes	Yes	Yes	Yes	Yes	Farmer	
No	No	Yes	Yes	Yes	Yes	Yes	Farmer	
Yes	No	Yes	Yes	Yes	Yes	Yes	Farmer	
No	No	Yes	Yes	Yes	Yes	Yes	Farmer	
No	Yes	Yes	Yes	Yes	No	Yes	Salaried job	
No	No	Yes	Yes	Yes	Yes	Yes	Farmer	
No	Yes	Yes	Yes	Yes	Yes	Yes	Farmer	
No	Yes	Yes	No	No	No	Yes	Farmer	
No	Yes	No	No	No	No	Yes	Pension	
No	No	Yes	No	No	No	No	Farmer	
No	No	Yes	No	No	No	Yes	Farmer	
No	No	Yes	No	No	No	Yes	Salaried job	
No	No	Yes	No	No	No	No	Farmer	
Yes	Yes	Yes	Yes	Yes	Yes	Yes	Farmer	
Yes	Yes	Yes	Yes	Yes	Yes	Yes	Farmer	
No	No	Yes	No	No	Yes	No	Farmer	
No	No	Yes	No	No	No	No	Others	Artisan
No	No	Yes	No	No	No	Yes	Salaried job	
No	No	Yes	No	No	No	No	Salaried job	
No	No	No	No	No	Yes	Yes	Farmer	
No	No	Yes	No	No	Yes	Yes	Farmer	
No	No	Yes	No	No	No	No	Farmer	
No	No	Yes	No	No	No	No	Salaried job	
No	Yes	Yes	No	No	No	No	Salaried job	
No	Yes	No	No	No	No	No	Farmer	
No	Yes	Yes	No	No	No	No	Pension	
No	Yes	Yes	No	No	No	No	Farmer	

No	Yes	Yes	No	No	No	No	Farmer	
No	Yes	Yes	No	No	No	Yes	Farmer	
No	No	Yes	No	No	No	No	Farmer	
No	No	Yes	Yes	No	No	No	Salaried job	
No	Yes	Yes	No	No	Yes	Yes	Farmer	
No	Yes	Yes	No	No	No	No	Salaried job	
No	Yes	Yes	No	No	No	No	Farmer	
No	Yes	Yes	No	No	No	No	Others	Dry cleaner
No	Yes	Yes	No	No	Yes	Yes	Others	Casual labo
No	Yes	Yes	No	No	No	Yes	Others	Casual labo
No	Yes	Yes	No	No	Yes	No	Salaried job	
No	Yes	Yes	No	No	No	No	Salaried job	
No	No	Yes	Yes	Yes	Yes	Yes	Salaried job	
No	No	No	No	No	No	No	Others	Business m
No	No	Yes	No	No	No	Yes	Salaried job	
No	Yes	Yes	No	No	No	No	Farmer	
No	Yes	Yes	No	No	No	No	Farmer	
No	Yes	Yes	No	No	No	Yes	Farmer	
No	Yes	Yes	No	No	No	No	Farmer	
No	Yes	Yes	No	No	No	No	Farmer	
No	No	Yes	No	No	No	No	Farmer	
No	No	Yes	No	No	No	No	Farmer	
No	No	No	No	No	No	No	Others	Dependant
No	Yes	No	No	No	No	No	Farmer	
No	No	Yes	No	No	No	No	Farmer	
No	Yes	Yes	Yes	Yes	Yes	Yes	Farmer	
No	No	Yes	No	No	No	No	Farmer	
No	No	Yes	No	No	No	No	Farmer	
No	Yes	Yes	Yes	No	No	Yes	Farmer	
No	Yes	Yes	No	No	Yes	Yes	Farmer	
No	Yes	Yes	No	No	Yes	No	Farmer	
Yes	Yes	Yes	No	No	Yes	Yes	Farmer	
No	Yes	Yes	No	No	Yes	Yes	Farmer	
No	Yes	Yes	No	No	Yes	Yes	Farmer	
No	No	Yes	No	No	No	No	Farmer	
No	No	Yes	No	No	No	No	Farmer	
No	No	Yes	No	No	No	No	Farmer	
No	No	Yes	No	No	No	No	Farmer	
No	No	Yes	No	No	No	No	Farmer	
No	Yes	Yes	Yes	No	No	No	Farmer	
No	Yes	Yes	No	No	No	No	Farmer	
No	Yes	Yes	No	No	Yes	Yes	Farmer	
No	Yes	Yes	No	No	Yes	Yes	Farmer	
No	No	Yes	No	No	No	No	Farmer	
No	Yes	Yes	No	No	No	No	Farmer	
Yes	Yes	Yes	Yes	Yes	No	Yes	Others	Business m
No	No	No	No	No	No	No	Farmer	

No	No	Yes	No	No	No	No	Farmer
Yes	Yes	Yes	No	No	Yes	Yes	Salaried job
No	Yes	Yes	No	No	Yes	Yes	Pension
No	No	Yes	No	No	No	No	Salaried job
No	No	Yes	No	No	No	No	Farmer
No	No	Yes	No	No	No	No	Farmer
Yes	No	Yes	No	No	No	No	Salaried job
Yes	Yes	Yes	No	No	No	No	Farmer
No	Yes	Yes	No	No	Yes	Yes	Farmer
No	No	Yes	No	No	No	No	Pension
No	No	Yes	No	No	No	No	Farmer
No	Yes	Yes	No	No	No	No	Farmer
No	Yes	Yes	No	No	Yes	Yes	Farmer
No	No	Yes	No	No	No	No	Farmer
Yes	Yes	Yes	No	No	No	Yes	Farmer
No	No	Yes	No	No	No	Yes	Salaried job
No	No	Yes	No	No	No	No	Salaried job
No	Yes	Yes	No	No	No	Yes	Farmer
No	No	Yes	No	No	No	No	Farmer
No	Yes	Yes	No	No	Yes	No	Farmer
No	No	Yes	No	No	No	No	Farmer
No	Yes	Yes	No	No	Yes	Yes	Salaried jol Farmer
No	Yes	Yes	No	No	Yes	Yes	Farmer
No	Yes	Yes	No	No	No	No	Farmer
No	Yes	Yes	No	No	Yes	Yes	Farmer
No	No	Yes	No	Yes	No	No	Farmer
No	Yes	Yes	No	No	No	No	Farmer
Yes	No	Yes	Yes	Yes	No	No	Farmer
No	Yes	Yes	No	No	No	Yes	Farmer
No	Yes	Yes	Yes	No	No	No	Merchant
No	Yes	Yes	Yes	No	Yes	No	Salaried job
No	Yes	Yes	No	No	Yes	No	Farmer
Yes	Yes	Yes	No	No	Yes	No	Farmer
No	No	Yes	No	No	Yes	No	Farmer
No	Yes	Yes	No	No	Yes	Yes	Farmer
No	No	Yes	No	No	No	No	Farmer
No	No	Yes	No	No	No	No	Farmer
No	Yes	Yes	No	No	No	No	Farmer
No	Yes	Yes	No	No	No	No	Farmer
No	No	No	No	No	No	No	Farmer
No	No	Yes	No	No	No	No	Farmer
Yes	Yes	Yes	Yes	Yes	Yes	Yes	Farmer
Yes	Yes	Yes	Yes	Yes	Yes	Yes	Farmer
No	Yes	Yes	No	No	Yes	Yes	Farmer
No	No	Yes	No	No	No	No	Farmer
Yes	Yes	Yes	No	No	No	No	Farmer
No	No	Yes	No	No	No	No	Farmer

No	No	Yes	No	No	No	No	Salaried job	
Yes	Yes	Yes	No	No	No	Yes	Farmer	
No	Yes	No	No	No	No	No	Farmer	
No	Yes	Yes	No	No	Yes	Yes	Farmer	
Yes	Yes	Yes	Yes	No	Yes	Yes	Farmer	
No	No	Yes	No	No	No	No	Farmer	
Yes	Yes	Yes	No	No	Yes	No	Farmer	
No	No	Yes	No	No	No	No	Farmer	
Yes	Yes	Yes	No	No	Yes	Yes	Others	Business m
No	No	Yes	No	No	No	Yes	Farmer	
No	No	Yes	No	No	Yes	No	Farmer	
No	No	Yes	No	No	No	No	Farmer	
Yes	No	Yes	Yes	No	Yes	Yes	Farmer	
No	No	Yes	No	No	No	No	Farmer	
No	Yes	Yes	No	No	No	No	Farmer	
No	Yes	Yes	No	No	No	No	Farmer	
No	No	Yes	No	No	Yes	Yes	Farmer	
No	No	Yes	No	No	Yes	Yes	Salaried job	
No	Yes	No	No	No	No	No	Salaried job	
No	Yes	Yes	No	No	No	No	Farmer	
No	Yes	Yes	No	No	Yes	No	Farmer	
No	No	Yes	No	No	Yes	No	Merchant	
No	Yes	Yes	No	No	No	No	Farmer	
No	No	Yes	No	No	No	No	Farmer	
No	Yes	Yes	No	No	Yes	Yes	Farmer	
Yes	Yes	Yes	No	No	Yes	Yes	Farmer	
Yes	Yes	Yes	No	No	Yes	Yes	Others	Business m
Yes	Yes	Yes	No	No	Yes	Yes	Farmer	
No	Yes	Yes	No	No	Yes	Yes	Farmer	
Yes	Yes	No	No	No	No	Yes	Salaried job	
Yes	Yes	Yes	No	No	No	Yes	Others	Business m
Yes	Yes	Yes	No	No	No	No	Farmer	
No	Yes	Yes	No	No	No	No	Salaried job	

cattle	sheep	goats	chicken	bicycle	radio	tv	specify_oth	monthly_in
1	3	4	30	0	1	1		Over 1500
3	0	3	25	0	1	0		0-5000
0	0	0	0	0	0	0		0-5000
2	0	2	4	0	1	1		0-5000
6	0	5	13	1	1	0		0-5000
1	0	0	10	0	0	0		0-5000
3	2	0	10	0	1	0		5001-1000
0	0	2	9	0	1	0		0-5000
0	0	0	0	0	0	0		Over 1500
5	0	8	13	0	1	0		0-5000
2	0	0	10	0	1	0		0-5000
3	0	4	9	1	1	0		0-5000
0	1	0	6	0	1	0		0-5000
8	10	0	16	0	1	0		0-5000
1	4	0	8	0	1	0		0-5000
2	6	0	12	0	0	0		0-5000
0	0	4	9	0	0	0		0-5000
2	0	0	3	0	1	0		0-5000
2	0	0	10	0	1	0		0-5000
1	0	0	0	0	1	0		0-5000
6	14	0	18	1	1	0		0-5000
4	0	0	18	0	1	0		0-5000
3	6	0	11	0	0	0		0-5000
0	4	11	18	0	1	0		0-5000
4	2	10	18	0	1	1		0-5000
0	2	0	11	0	0	0		0-5000
2	0	0	7	0	0	0		0-5000
2	0	0	5	0	0	0		0-5000
0	2	0	0	0	1	0		0-5000
0	0	0	0	0	0	1		0-5000
0	0	0	2	0	1	0		0-5000
2	0	0	0	1	0	0		5001-1000
4	1	0	0	1	1	0		0-5000
2	0	0	6	1	1	1		5001-1000
3	6	0	7	0	1	1	Motorbike	0-5000
0	0	0	0	0	1	0		5001-1000
0	0	0	20	1	1	1		5001-1000
8	0	0	20	1	1	0		5001-1000
6	0	10	14	0	1	0		0-5000
8	4	0	20	1	1	1		5001-1000
8	18	0	12	1	1	1		10001-150
4	18	12	11	1	1	1		Over 1500
0	0	0	0	0	1	0		0-5000
5	15	0	18	0	1	1		Over 1500
4	3	0	10	0	1	1		Over 1500
4	9	6	18	1	1	1		5001-1000

6	0	0	14	0	1	0		0-5000
2	0	8	12	0	1	0		0-5000
4	16	0	14	0	1	0		0-5000
0	0	0	8	0	1	0		5001-1000
1	0	0	12	0	0	0		0-5000
12	0	0	18	0	1	1		Over 1500
2	6	0	12	0	0	0		0-5000
1	0	0	4	0	1	0		0-5000
1	2	5	10	0	1	0		0-5000
2	3	0	0	0	0	0		5001-1000
0	1	0	7	0	0	0		0-5000
2	1	0	5	0	1	1		5001-1000
0	0	0	0	0	0	0		0-5000
1	0	0	0	0	1	0		0-5000
0	0	0	0	0	0	0		5001-1000
5	0	0	2	0	1	0		5001-1000
2	3	0	0	1	1	0		0-5000
1	0	0	0	0	0	0		0-5000
1	3	0	3	0	0	0		0-5000
1	2	0	0	0	1	0		0-5000
2	4	1	18		1	1		0-5000
2	0	0	10	1	1	1		0-5000
5	0	0	29	0	1	1		10001-150
1	2	0	5	0	1	1	Car	Over 1500
0	0	1	6	0	1	0		0-5000
2	1	10	15	1	1	1		0-5000
1	3	0	20	0	1	1		Over 1500
3	2	5	26	0	0	1		0-5000
3	4	9	16	1	1	1		5001-1000
2	0	3	18	1	1	1		5001-1000
4	1	4	18	1	1	1		Over 1500
0	2	0	20	0	1	1		Over 1500
0	0	0	3	0	1	0		0-5000
3	60	0	4	0	1	0		0-5000
2	0	0	10	0	1	1		5001-1000
1	2	1	0	0	1	0		0-5000
3	0	0	0	0	1	0		0-5000
2	2	0	20	1	1	1		0-5000
1	0	1	2	0	0	0		0-5000
0	0	2	7	0	1	0		0-5000
0	0	0	13	0	0	0		0-5000
0	0	1	1	0	0	0		0-5000
0	0	0	0	0	2	0		0-5000
1	0	0	10	0	1	0		0-5000
1	0	0	8	0	1	0		0-5000
0	0	0	0	1	1	1		0-5000
0	0	0	0	1	1	1		0-5000

0	0	1	6	0	0	1		0-5000
2	0	0	12	0	1	1		0-5000
0	0	0	7	0	1	1		0-5000
1	0	0	1	1	0	0		0-5000
1	1	0	5	0	0	0		0-5000
2	0	0	20	1	1	1		0-5000
0	0	2	4	0	0	0		0-5000
2	0	4	16	1	1	0		0-5000
0	0	5	11	0	1	0		0-5000
2	1	1	2	0	0	0		0-5000
2	0	8	20	1	1	0		0-5000
10	3	15	10	2	1	1		5001-1000
2	0	5	10	1	1	1		5001-1000
0	0	6	8	1	1	0		5001-1000
5	0	10	20	0	0	0		5001-1000
3	0	25	10	0	1	0	Car	5001-1000
2	0	7	10	1	1	1		5001-1000
1	0	1	0	0	1	0		0-5000
0	0	3	4	0	1	0		0-5000
7	0	5	8	1	1	1		5001-1000
0	1	1	0	0	1	0		0-5000
2	0	0	3	0	0	0		0-5000
0	0	0	1	1	0	0		0-5000
0	0	0	6	1	0	0		0-5000
0	0	0	0	0	0	0		0-5000
2	0	10	20	0	1	0		0-5000
0	0	0	0	0	0	0		0-5000
0	0	5	15	0	1	1		0-5000
0	0	7	10	1	1	0		0-5000
4	0	2	20	1	2	1		0-5000
1	0	2	10	0	1	0		0-5000
0	0	2	6	0	1	0		0-5000
3	0	0	35	7	2	1		5001-1000
0	0	0	5	0	2	0		0-5000
8	0	2	15	1	1	1		5001-1000
0	3	4	2	1	0	0		0-5000
1	0	0	9	2	1	1		0-5000
0	0	1	0	1	1	0		0-5000
0	0	1	3	0	0	0		0-5000
4	0	4	2	0	3	1		0-5000
2	2	0	10	0	1	1		0-5000
0	0	0	0	0	0	0		0-5000
4	0	1	10	1	3	2		0-5000
0	0	5	0	0	1	0		0-5000
2	0	4	7	0	1	1		0-5000
0	0	1	6	0	1	0		0-5000
3	0	3	10	0	1	1		0-5000

2	1	0	2	0	1	0	0-5000
2	0	0	10	2	1	0	0-5000
2	0	1	10	0	1	1	Over 1500(
2	3	3	5	0	0	0	0-5000
1	0	0	2	0	1	0	0-5000
0	0	3	1	0	1	1	0-5000
0	0	0	0	1	0	0	0-5000
2	0	3	30	0	1	1	Over 1500(
0	0	2	6	0	1	0	0-5000
2	0	1	20	0	1	1	Over 1500(
0	0	0	15	0	1	1	0-5000
2	0	0	7	0	1	0	0-5000
2	0	0	0	0	1	1	0-5000
0	0	0	0	0	0	1	0-5000
0	0	0	1	0	1	0	5001-1000(
0	0	0	0	0	1	1	0-5000
0	0	0	15	0	1	0	0-5000
0	0	6	0	0	0	0	0-5000
2	0	2	5	0	0	0	0-5000
2	1	1	0	0	0	0	0-5000
2	1	1	1	0	0	0	0-5000
1	1	2	0	0	0	0	0-5000
1	2	1	1	0	0	0	0-5000
0	0	2	1	0	0	0	0-5000
2	0	0	11	0	1	0	0-5000
0	0	2	3	0	0	0	0-5000
0	1	2	1	1	0	0	0-5000
0	0	4	6	0	0	0	0-5000
6	0	22	18	1	1	0	5001-1000(
5	0	1	20	1	1	0	0-5000
0	0	4	12	1	0	0	0-5000
0	0	2	6	0	1	0	5001-1000(
0	0	7	12	1	2	0	0-5000
2	1	10	5	0	1	0	5001-1000(
3	2	5	1	0	1	0	5001-1000(
0	5	10	0	1	0	1	0-5000
0	0	10	10	1	1	0	0-5000
0	0	4	5	0	1	0	Over 1500(
5	0	3	6	0	1	0	10001-150(
8	2	14	20	2	3	0	5001-1000(
1	2	0	5	0	0	0	0-5000
2	0	3	4	0	1	0	0-5000
2	0	3	20	1	1	1	5001-1000(
0	0	8	7	1	1	0	5001-1000(
8	0	0	25	0	0	1	5001-1000(
7	0	1	10	1	2	1	5001-1000(
8	0	1	6	2	1	0	10001-150(

2	3	3	5	1	1	0		0-5000
2	0	3	18	0	1	0		0-5000
0	0	4	0	0	1	0		0-5000
1	0	0	0	1	1	1		0-5000
3	0	11	15	0	0	0		0-5000
3	0	1	12	1	1	0		5001-1000(
1	0	5	6	1	1	0		0-5000
0	0	3	0	0	1	0		0-5000
2	0	2	5	0	1	0		10001-150(
0	0	0	2	0	0	0		0-5000
0	0	0	40	0	1	1		Over 1500(
4	5	7	23	1	1	1		Over 1500(
0	0	0	4	0	1	1		10001-150(
4	3	0	4	0	0	0		0-5000
2	0	0	10	0	1	0		Over 1500(
2	0	0	20	1	1	0		0-5000
2	0	10	6	0	1	0		0-5000
1	0	10	6	0	1	0		0-5000
0	0	2	6	0	1	0		0-5000
1	0	3	16	0	1	0		0-5000
1	0	6	7	0	0	0		0-5000
2	0	2	25	1	1	0		0-5000
2	0	4	15	1	1	0		0-5000
4	1	2	25	0	1	1		10001-150(
0	2	5	10	0	1	0		5001-1000(
1	2	3	100	1	1	1		Over 1500(
0	0	2	25	0	1	0		0-5000
0	0	0	10	1	1	1		0-5000
0	0	4	6	0	1	0		0-5000
3	6	0	20	0	1	1	Tractor	10001-150(
3	4	0	10	1	1	1		5001-1000(
4	4	0	10	0	0	0		5001-1000(
8	4	1	10	0	1	1		10001-150(
3	7	0	12	1	0	0		5001-1000(
1	0	0	2	0	0	0		0-5000
1	0	0	5	0	0	0		0-5000
2	0	0	5	1	1	0		5001-1000(
8	0	0	15	1	1	1		5001-1000(
3	0	0	5	0	1	1		0-5000
5	0	0	40	3	4	1		Over 1500(
3	0	6	14	1	1	0		0-5000
2	3	7	13	1	1	0		5001-1000(
2	0	4	5	0	1	0		0-5000
7	0	0	25	1	1	1		Over 1500(
5	11	0	11	1	1	1		Over 1500(
0	0	4	10	0	1	1		0-5000
2	2	0	10	0	1	0		0-5000

1	2	6	0	0	0	1		5001-1000
3	6	4	20	2	1	1		Over 1500
4	11	0	9	0	1	1		10001-150
3	0	0	5	0	1	1		Over 1500
0	0	0	0	0	0	0		0-5000
5	2	0	10	1	1	1		10001-150
2	0	2	25	1	1	1		5001-1000
3	4	0	12	1	1	0		5001-1000
4	7	0	9	0	1	1		5001-1000
5	0	0	15	0	0	0		0-5000
0	0	0	7	0	0	0		0-5000
3	0	2	8	0	0	0		0-5000
5	7	3	11	0	1	1		5001-1000
3	0	0	0	0	0	0		0-5000
1	0	0	3	1	1	0		0-5000
1	1	10	3	1	1	1	Motorbike	5001-1000
3	3	0	5	0	1	1		Over 1500
2	2	0	3	0	1	0		0-5000
1	0	0	10	0	1	1		0-5000
3	0	1	5	1	2	0		0-5000
6	1	0	0	0	1	1		10001-150
6	0	0	5	1	1	1		Over 1500
0	0	4	14	1	1	0		5001-1000
4	5	0	40	1	1	1		10001-150
1	0	0	5	0	0	0		0-5000
1	1	1	6	1	1	1		5001-1000
8	7	0	30	1	1	1		Over 1500
1	2	4	5	0	1	1		0-5000
0	0	0	0	0	0	0		0-5000
6	4	3	13	1	1	0		0-5000
4	0	0	18	0	1	1		Over 1500
2	3	1	3	0	0	0		0-5000
10	7	2	15	2	1	1		0-5000
3	0	7	10	1	2	1		5001-1000
7	3	6	4	0	1	1		0-5000
5	0	0	10	0	0	0		0-5000
3	2	0	5	1	1	0		Over 1500
5	0	0	20	0	0	0		0-5000
3	0	0	0	0	0	0		0-5000
0	0	0	2	0	1	0		0-5000
3	0	0	10	0	1	0		5001-1000
2	2	0	10	0	0	0		10001-150
2	4	0	12	0	1	0		5001-1000
3	5	4	14	1	0	1		5001-1000
0	2	0	8	0	0	0		0-5000
2	0	0	20	0	1	0		0-5000
3	0	0	10	1	1	0		5001-1000

2	0	0	10	0	1	1	10001-150
0	0	0	0	0	0	0	0-5000
6	12	1	32	0	1	1	5001-1000
6	7	23	110	1	1	1	Over 1500
3	4	0	0	0	0	0	5001-1000
3	0	0	12	0	1	1	5001-1000
6	0	0	1	2	1	0	0-5000
5	1	0	12	1	1	1	10001-150
1	0	0	0	0	1	1	10001-150
0	0	0	20	0	1	1	5001-1000
2	0	2	18	1	1	1	0-5000
3	0	0	20	0	1	1	0-5000
0	0	1	3	0	1	0	0-5000
0	0	0	5	0	0	0	0-5000
1	2	2	10	0	1	0	0-5000
8	1	7	15	1	1	0	0-5000
5	3	1	10	1	1	1	0-5000
0	0	0	2	0	1	1	Over 1500
0	0	0	0	0	0	1	0-5000
0	0	1	3	0	1	0	0-5000
4	0	5	10	0	1	0	0-5000
1	0	0	2	0	1	0	0-5000
0	1	0	0	0	1	0	0-5000
3	0	1	6	1	1	0	5001-1000
2	0	0	0	0	0	0	5001-1000
2	3	0	10	0	0	0	5001-1000
3	5	0	10	0	0	0	10001-150
0	0	0	0	0	0	0	5001-1000
2	5	3	20	0	1	1	10001-150
1	0	0	10	1	0	1	10001-150
1	0	0	10	1	1	1	10001-150
0	0	0	7	1	1	0	0-5000
10	8	0	40	1	1	0	Over 1500

own_land	residence	type_of_hc	electricity	solar_pane	lighting_ke	specify_otl	firewood	gas
1	Self owned	Permanent	Yes	No	No	N/A	Yes	No
1	Self owned	Permanent	No	Yes	No	N/A	Yes	No
1	Self owned	Semi- perm	No	No	Yes	N/A	Yes	No
1	Self owned	Semi- perm	No	Yes	No		Yes	No
1	Self owned	Temporary	No	Yes	No	N/A	Yes	No
1	Self owned	Temporary	No	No	Yes	N/A	Yes	No
1	Self owned	Semi- perm	No	Yes	No	N/A	Yes	No
1	Self owned	Temporary	No	Yes	No	N/A	Yes	No
1	Self owned	Semi- perm	No	Yes	No	N/A	Yes	No
1	Self owned	Temporary	No	No	Yes	N/A	Yes	No
1	Self owned	Temporary	No	No	Yes	N/A	Yes	No
1	Self owned	Semi- perm	No	Yes	No	N/A	Yes	No
1	Self owned	Semi- perm	No	Yes	No	N/A	Yes	No
1	Self owned	Semi- perm	No	Yes	No	N/A	Yes	No
1	Self owned	Semi- perm	No	Yes	No	N/A	Yes	No
1	Self owned	Temporary	No	No	Yes	N/A	Yes	No
1	Self owned	Temporary	No	Yes	No	N/A	Yes	No
1	Self owned	Semi- perm	No	Yes	No	N/A	Yes	No
1	Self owned	Semi- perm	No	Yes	No	N/A	Yes	No
1	Self owned	Semi- perm	No	No	Yes	N/A	Yes	No
1	Self owned	Semi- perm	No	Yes	No	N/A	Yes	No
1	Self owned	Semi- perm	No	Yes	No	N/A	Yes	No
1	Self owned	Semi- perm	No	No	Yes	N/A	Yes	No
1	Self owned	Semi- perm	No	Yes	No	N/A	Yes	No
1	Self owned	Semi- perm	No	Yes	No	N/A	Yes	No
1	Self owned	Temporary	No	No	Yes	N/A	Yes	No
1	Self owned	Semi- perm	No	Yes	No	N/A	Yes	No
1	Self owned	Semi- perm	No	Yes	No	N/A	Yes	No
1	Self owned	Semi- perm	No	No	Yes	N/A	Yes	No
1	Self owned	Semi- perm	No	No	Yes	N/A	Yes	No
1	Self owned	Semi- perm	No	Yes	No	N/A	Yes	No
1	Self owned	Semi- perm	No	Yes	No	N/A	Yes	No
1	Self owned	Semi- perm	No	No	Yes	N/A	Yes	No
1	Self owned	Semi- perm	No	No	Yes	N/A	Yes	No
1	Self owned	Semi- perm	No	Yes	No	N/A	Yes	No
1	Self owned	Semi- perm	No	No	Yes	N/A	Yes	No
1	Self owned	Permanent	Yes	No	No	N/A	Yes	No
1	Self owned	Semi- perm	No	Yes	No	N/A	Yes	No
1	Self owned	Temporary	No	No	Yes	N/A	Yes	No
1	Self owned	Semi- perm	No	Yes	No	N/A	Yes	No
1	Self owned	Semi- perm	No	Yes	No	N/A	Yes	No
1	Self owned	Semi- perm	No	Yes	No	N/A	Yes	No
1	Self owned	Temporary	No	Yes	No	N/A	Yes	No
1	Self owned	Semi- perm	No	Yes	No	N/A	Yes	No
1	Self owned	Semi- perm	No	Yes	No	N/A	Yes	No
1	Self owned	Semi- perm	No	Yes	No	N/A	Yes	No

1	Self owned	Semi- perm	No	Yes	No	N/A	Yes	No
1	Self owned	Temporary	No	Yes	No	N/A	Yes	No
1	Self owned	Semi- perm	No	Yes	No	N/A	Yes	No
1	Self owned	Temporary	No	Yes	No	N/A	Yes	No
1	Self owned	Temporary	No	No	Yes	N/A	Yes	No
1	Self owned	Semi- perm	No	Yes	No	N/A	Yes	No
1	Self owned	Semi- perm	No	No	No	N/A	Yes	No
1	Self owned	Semi- perm	No	Yes	No	N/A	Yes	No
1	Self owned	Semi- perm	No	No	No	N/A	Yes	No
1	Self owned	Semi- perm	No	Yes	No	N/A	Yes	No
1	Self owned	Semi- perm	No	Yes	No	N/A	Yes	No
1	Self owned	Semi- perm	No	Yes	No	N/A	Yes	No
1	Self owned	Semi- perm	No	Yes	No	N/A	Yes	No
1	Self owned	Permanent	No	Yes	No	N/A	Yes	No
1	Self owned	Semi- perm	No	Yes	No	N/A	Yes	No
1	Self owned	Permanent	Yes	No	No	N/A	No	Yes
1	Self owned	Semi- perm	No	Yes	No	N/A	Yes	No
1	Self owned	Semi- perm	No	No	Yes	N/A	Yes	No
1	Self owned	Semi- perm	No	No	Yes	N/A	Yes	No
1	Self owned	Semi- perm	No	No	Yes	N/A	Yes	No
1	Self owned	Permanent	Yes	No	No	N/A	Yes	No
1	Self owned	Semi- perm	No	Yes	No	N/A	Yes	No
1	Self owned	Permanent	Yes	No	No	N/A	No	Yes
1	Self owned	Permanent	Yes	No	No	N/A	Yes	Yes
1	Self owned	Permanent	No	Yes	No	N/A	Yes	No
1	Self owned	Permanent	No	No	Yes	N/A	Yes	No
1	Self owned	Permanent	Yes	No	No	N/A	Yes	No
1	Self owned	Semi- perm	No	No	Yes	N/A	Yes	No
1	Self owned	Permanent	No	No	Yes	N/A	Yes	No
1	Self owned	Permanent	No	Yes	No	N/A	Yes	No
1	Self owned	Permanent	Yes	No	No	N/A	Yes	No
1	Self owned	Semi- perm	Yes	No	No	N/A	Yes	No
1	Self owned	Semi- perm	No	No	Yes	N/A	Yes	No
1	Self owned	Semi- perm	No	No	Yes	N/A	Yes	No
1	Self owned	Semi- perm	No	Yes	No	N/A	Yes	No
1	Self owned	Semi- perm	No	No	No	N/A	No	No
1	Self owned	Semi- perm	No	No	Yes	N/A	Yes	No
1	Self owned	Semi- perm	No	No	Yes	N/A	Yes	No
1	Self owned	Semi- perm	No	No	Yes	N/A	Yes	No
1	Self owned	Semi- perm	No	No	Yes	N/A	Yes	No
1	Self owned	Temporary	No	No	Yes	N/A	Yes	No
1	Self owned	Temporary	No	No	Yes	N/A	Yes	No
1	Self owned	Permanent	Yes	No	No	N/A	Yes	No
1	Self owned	Semi- perm	No	No	Yes	N/A	Yes	No
1	Self owned	Semi- perm	Yes	No	No	N/A	Yes	No
1	Self owned	Semi- perm	No	No	Yes	N/A	Yes	No
1	Self owned	Permanent	No	Yes	No	N/A	Yes	No

1	Self owned	Semi- perm	Yes	No	No	N/A	Yes	No
1	Self owned	Permanent	Yes	No	No	N/A	Yes	No
1	Self owned	Semi- perm	Yes	No	No	N/A	Yes	No
1	Self owned	Semi- perm	Yes	No	No	N/A	Yes	No
0	Self owned	Semi- perm	Yes	No	No	N/A	Yes	No
1	Self owned	Permanent	Yes	No	No	N/A	Yes	No
1	Self owned	Semi- perm	No	Yes	No	N/A	Yes	No
1	Self owned	Semi- perm	No	Yes	No	N/A	Yes	No
1	Self owned	Semi- perm	No	No	Yes	N/A	Yes	No
1	Self owned	Semi- perm	No	No	Yes	N/A	Yes	No
1	Self owned	Semi- perm	No	No	Yes	N/A	Yes	No
1	Self owned	Semi- perm	No	No	Yes	N/A	Yes	No
1	Self owned	Semi- perm	No	Yes	No	N/A	Yes	No
1	Self owned	Semi- perm	No	No	Yes	N/A	Yes	No
1	Self owned	Semi- perm	No	No	Yes	N/A	Yes	No
1	Self owned	Permanent	No	Yes	No	N/A	Yes	No
1	Self owned	Semi- perm	No	No	Yes	N/A	Yes	No
1	Self owned	Permanent	No	No	Yes	N/A	Yes	No
1	Self owned	Semi- perm	No	No	Yes	N/A	Yes	No
1	Self owned	Permanent	No	Yes	No	N/A	Yes	No
1	Self owned	Permanent	No	Yes	No	N/A	Yes	No
1	Self owned	Temporary	No	No	Yes	N/A	Yes	No
1	Self owned	Temporary	No	No	Yes	N/A	Yes	No
1	Self owned	Permanent	No	No	Yes	N/A	Yes	No
1	Self owned	Permanent	No	No	Yes	N/A	Yes	No
1	Self owned	Permanent	No	Yes	No	N/A	Yes	No
1	Self owned	Semi- perm	No	No	Yes	N/A	Yes	No
1	Self owned	Permanent	No	Yes	No	N/A	Yes	No
1	Self owned	Permanent	No	Yes	No	N/A	Yes	No
1	Self owned	Semi- perm	Yes	No	No	N/A	Yes	No
1	Self owned	Semi- perm	No	No	Yes	N/A	Yes	No
1	Self owned	Permanent	No	No	Yes	N/A	Yes	No
1	Self owned	Permanent	No	No	Yes	N/A	Yes	Yes
1	Self owned	Permanent	No	No	Yes	N/A	Yes	No
1	Self owned	Permanent	No	Yes	No	N/A	Yes	Yes
1	Self owned	Permanent	Yes	No	No	N/A	Yes	No
1	Self owned	Permanent	Yes	No	No	N/A	Yes	Yes
1	Self owned	Semi- perm	No	No	Yes	N/A	Yes	No
1	Self owned	Semi- perm	No	No	Yes	N/A	Yes	No
1	Self owned	Permanent	No	No	Yes	N/A	Yes	No
1	Self owned	Permanent	Yes	No	No	N/A	Yes	No
1	Self owned	Permanent	Yes	No	No	N/A	Yes	No
1	Self owned	Permanent	Yes	No	No	N/A	Yes	No
1	Self owned	Temporary	No	No	Yes	N/A	Yes	No
1	Self owned	Permanent	Yes	No	No	N/A	Yes	No
1	Self owned	Permanent	No	No	Yes	N/A	Yes	No
1	Self owned	Permanent	Yes	No	No	N/A	Yes	No

1	Self owned	Permanent	No	No	Yes	N/A	Yes	No
1	Self owned	Permanent	Yes	No	No	N/A	Yes	No
1	Self owned	Semi- perm	No	No	Yes	N/A	Yes	No
1	Self owned	Semi- perm	No	No	Yes	N/A	Yes	No
1	Self owned	Permanent	Yes	No	No	N/A	Yes	No
1	Self owned	Permanent	Yes	No	No	N/A	Yes	No
1	Self owned	Permanent	Yes	No	No	N/A	Yes	No
1	Self owned	Semi- perm	Yes	No	No	N/A	Yes	No
1	Self owned	Semi- perm	No	No	Yes	N/A	Yes	No
1	Self owned	Permanent	Yes	No	No	N/A	Yes	No
1	Self owned	Permanent	Yes	No	No	N/A	Yes	Yes
1	Self owned	Permanent	Yes	No	No	N/A	Yes	No
1	Self owned	Permanent	Yes	No	No	N/A	Yes	No
1	Self owned	Permanent	Yes	No	No	N/A	Yes	No
1	Self owned	Semi- perm	No	No	Yes	N/A	Yes	No
1	Self owned	Semi- perm	No	No	Yes	N/A	Yes	No
1	Self owned	Semi- perm	No	No	Yes	N/A	Yes	No
1	Self owned	Temporary	No	No	Yes	N/A	Yes	No
1	Self owned	Permanent	No	No	Yes	N/A	Yes	No
1	Self owned	Semi- perm	No	No	Yes	N/A	Yes	No
1	Self owned	Semi- perm	No	No	Yes	N/A	Yes	No
1	Self owned	Semi- perm	No	No	Yes	N/A	Yes	No
1	Self owned	Semi- perm	No	No	Yes	N/A	Yes	No
1	Self owned	Semi- perm	No	No	Yes	N/A	Yes	No
1	Self owned	Semi- perm	No	Yes	No	N/A	Yes	No
1	Self owned	Temporary	No	No	Yes	N/A	Yes	No
1	Self owned	Semi- perm	No	No	Yes	N/A	Yes	No
1	Self owned	Temporary	No	No	Yes	N/A	Yes	No
1	Self owned	Permanent	No	Yes	No	N/A	Yes	No
1	Self owned	Permanent	No	Yes	No	N/A	Yes	No
1	Self owned	Semi- perm	No	Yes	No	N/A	Yes	No
1	Self owned	Semi- perm	No	Yes	No	N/A	Yes	No
1	Self owned	Semi- perm	No	Yes	No	N/A	Yes	No
1	Self owned	Semi- perm	No	No	Yes	N/A	Yes	No
1	Self owned	Semi- perm	No	No	Yes	N/A	Yes	No
1	Self owned	Semi- perm	No	Yes	No	N/A	Yes	No
1	Self owned	Semi- perm	No	Yes	No	N/A	Yes	No
1	Self owned	Permanent	No	Yes	No	N/A	Yes	No
1	Self owned	Semi- perm	No	No	Yes	N/A	Yes	No
1	Self owned	Permanent	No	Yes	No	N/A	Yes	No
1	Self owned	Semi- perm	No	No	Yes	N/A	Yes	No
1	Self owned	Semi- perm	No	No	Yes	N/A	Yes	No
1	Self owned	Permanent	No	Yes	No	N/A	No	No
1	Self owned	Permanent	No	Yes	No	N/A	Yes	No
1	Self owned	Permanent	No	Yes	No	N/A	Yes	No
1	Self owned	Permanent	No	Yes	No	N/A	Yes	No
1	Self owned	Permanent	No	Yes	No	N/A	No	No

1	Self owned	Permanent	No	Yes	No	N/A	Yes	No	
1	Self owned	Permanent	No	Yes	No	N/A	Yes	No	
1	Self owned	Permanent	No	Yes	No	N/A	Yes	No	
1	Self owned	Permanent	No	Yes	No	N/A	No	Yes	
1	Self owned	Permanent	No	Yes	No	N/A	Yes	No	
1	Self owned	Permanent	No	Yes	No	N/A	Yes	No	
1	Self owned	Permanent	No	No	Yes	N/A	Yes	No	
1	Self owned	Permanent	No	Yes	No	N/A	Yes	No	
1	Self owned	Permanent	No	Yes	No	N/A	Yes	No	
1	Self owned	Permanent	No	Yes	No	N/A	Yes	No	
1	Self owned	Permanent	Yes	No	No	N/A	No	Yes	
1	Self owned	Permanent	No	Yes	No	N/A	Yes	No	
1	Self owned	Permanent	Yes	No	No	N/A	No	Yes	
1	Self owned	Semi- perm	No	Yes	No	N/A	Yes	No	
1	Self owned	Permanent	No	Yes	No	N/A	No	No	
1	Self owned	Permanent	No	Yes	No	N/A	Yes	No	
1	Self owned	Semi- perm	Yes	No	No	N/A	Yes	No	
1	Self owned	Semi- perm	No	No	Yes	N/A	Yes	No	
1	Self owned	Permanent	No	No	Yes	N/A	Yes	No	
1	Self owned	Permanent	No	No	Yes	N/A	Yes	No	
1	Self owned	Semi- perm	No	No	Yes	N/A	Yes	No	
1	Self owned	Semi- perm	No	Yes	No	N/A	Yes	No	
1	Self owned	Semi- perm	No	No	Yes	N/A	Yes	No	
1	Self owned	Permanent	No	Yes	No	N/A	Yes	Yes	
1	Self owned	Permanent	No	Yes	No	N/A	Yes	No	
1	Self owned	Permanent	No	Yes	No	N/A	Yes	No	
0	From famil	Semi- perm	No	No	Yes	N/A	Yes	No	
1	Self owned	Permanent	No	Yes	No	N/A	No	Yes	
1	Self owned	Semi- perm	Yes	No	No	N/A	No	No	
1	Self owned	Permanent	Yes	No	No	N/A	Yes	No	
1	Self owned	Semi- perm	No	Yes	No	N/A	Yes	No	
1	Self owned	Semi- perm	No	No	Yes	N/A	Yes	No	
1	Self owned	Permanent	No	Yes	No	N/A	Yes	No	
1	Self owned	Semi- perm	No	No	Yes	N/A	Yes	No	
1	Self owned	Semi- perm	Yes	No	No	N/A	Yes	No	
1	Self owned	Semi- perm	No	Yes	No	N/A	Yes	No	
1	Self owned	Semi- perm	Yes	No	No	N/A	Yes	Yes	
1	Self owned	Permanent	Yes	No	No	N/A	Yes	Yes	
1	Self owned	Permanent	Yes	No	No	N/A	Yes	No	
1	Self owned	Permanent	Yes	No	No	N/A	Yes	Yes	
1	Self owned	Semi- perm	No	Yes	No	N/A	Yes	No	
1	Self owned	Temporary	No	No	Yes	N/A	Yes	No	
1	Self owned	Temporary	No	No	Yes	N/A	Yes	No	
1	Self owned	Permanent	No	Yes	No	N/A	Yes	No	
1	Self owned	Semi- perm	No	No	No	N/A	Yes	No	
1	Self owned	Semi- perm	No	No	Yes	N/A	Yes	No	
1	Self owned	Semi- perm	No	Yes	No	N/A	Yes	No	

1	Self owned	Semi- perm	Yes	No	Yes	N/A	Yes	No
1	Self owned	Permanent	Yes	No	No	N/A	Yes	No
1	Self owned	Permanent	Yes	No	No	N/A	Yes	Yes
1	Self owned	Permanent	Yes	No	No	N/A	Yes	No
1	Self owned	Permanent	No	Yes	No	N/A	Yes	No
1	Self owned	Permanent	Yes	No	No	N/A	Yes	No
1	Self owned	Permanent	Yes	No	No	N/A	Yes	Yes
1	Self owned	Semi- perm	Yes	No	No	N/A	Yes	No
1	Self owned	Permanent	No	Yes	No	N/A	Yes	No
1	Self owned	Permanent	Yes	No	No	N/A	Yes	No
1	Self owned	Semi- perm	No	No	Yes	N/A	Yes	No
1	Self owned	Temporary	No	No	Yes	N/A	Yes	No
0	Rented	Permanent	Yes	No	No	N/A	No	No
1	Self owned	Semi- perm	No	No	Yes	N/A	Yes	No
1	Self owned	Semi- perm	No	No	Yes	N/A	Yes	No
1	Self owned	Permanent	Yes	No	No	N/A	Yes	No
1	Self owned	Permanent	Yes	No	No	N/A	No	No
1	Self owned	Semi- perm	No	No	Yes	N/A	Yes	No
1	Self owned	Permanent	Yes	No	No	N/A	Yes	No
1	Self owned	Permanent	No	Yes	No	N/A	Yes	No
1	Self owned	Semi- perm	Yes	No	No	N/A	Yes	No
1	Self owned	Permanent	Yes	No	No	N/A	Yes	No
0	Rented	Semi- perm	No	No	Yes	N/A	Yes	No
1	Self owned	Semi- perm	Yes	No	No	N/A	Yes	No
1	Self owned	Temporary	No	No	Yes	N/A	Yes	No
1	Self owned	Semi- perm	No	No	Yes	N/A	Yes	No
1	Self owned	Permanent	Yes	No	No	N/A	Yes	No
1	Self owned	Permanent	Yes	No	No	N/A	Yes	No
1	Self owned	Semi- perm	Yes	No	No	N/A	Yes	No
1	Self owned	Semi- perm	No	Yes	No	N/A	Yes	No
1	Self owned	Semi- perm	No	Yes	No	N/A	Yes	No
1	Self owned	Permanent	Yes	No	No	N/A	Yes	No
1	Self owned	Permanent	Yes	No	No	N/A	Yes	No
1	Self owned	Permanent	No	Yes	No	N/A	Yes	No
1	Self owned	Semi- perm	Yes	No	No	N/A	No	Yes
1	Self owned	Semi- perm	No	Yes	No	N/A	Yes	No
1	Self owned	Semi- perm	No	Yes	No	N/A	Yes	No
1	Self owned	Permanent	Yes	No	No	N/A	Yes	No
1	Self owned	Semi- perm	No	Yes	No	N/A	Yes	No
0	Rented	Semi- perm	No	Yes	No	N/A	Yes	No
1	Self owned	Semi- perm	Yes	No	No	N/A	Yes	No
1	Self owned	Semi- perm	No	Yes	Yes	N/A	Yes	No
1	Self owned	Semi- perm	No	No	Yes	N/A	Yes	No
1	Self owned	Semi- perm	No	Yes	No	N/A	Yes	No
1	Self owned	Semi- perm	No	Yes	No	N/A	Yes	No
1	Self owned	Semi- perm	No	No	Yes	N/A	Yes	No
1	Self owned	Permanent	Yes	No	No	N/A	Yes	No

1	Self owned	Permanent	Yes	No	No	N/A	Yes	Yes	
1	Self owned	Semi- perm	No	No	Yes	N/A	Yes	No	
1	Self owned	Semi- perm	Yes	No	No	N/A	Yes	No	
1	Self owned	Permanent	No	Yes	No	N/A	Yes	Yes	
1	Self owned	Semi- perm	No	Yes	Yes	N/A	Yes	No	
1	Self owned	Permanent	Yes	No	No	N/A	Yes	No	
1	Self owned	Semi- perm	No	Yes	No	N/A	Yes	No	
1	Self owned	Semi- perm	Yes	No	No	N/A	No	No	
1	Self owned	Semi- perm	No	Yes	Yes	N/A	Yes	No	
1	Self owned	Semi- perm	Yes	No	No	N/A	Yes	No	
1	Self owned	Semi- perm	Yes	No	No	N/A	Yes	No	
1	Self owned	Permanent	Yes	No	No	N/A	Yes	No	
1	Self owned	Permanent	Yes	No	No	N/A	Yes	No	
1	Self owned	Semi- perm	No	Yes	No	N/A	Yes	No	
1	Self owned	Semi- perm	No	No	Yes	N/A	Yes	No	
1	Self owned	Semi- perm	No	No	Yes	N/A	Yes	No	
1	Self owned	Permanent	Yes	No	No	N/A	Yes	Yes	
1	Self owned	Permanent	No	Yes	No	N/A	Yes	No	
1	Self owned	Permanent	No	Yes	No	N/A	Yes	No	
1	Self owned	Permanent	No	Yes	No	N/A	Yes	No	
1	Self owned	Permanent	No	Yes	Yes	N/A	Yes	No	
1	Self owned	Semi- perm	No	No	No	N/A	No	No	
1	Self owned	Semi- perm	No	No	No	N/A	No	No	
1	Self owned	Semi- perm	Yes	No	No	N/A	Yes	No	
1	Self owned	Semi- perm	No	Yes	Yes	N/A	Yes	No	
1	Self owned	Semi- perm	No	No	Yes	N/A	Yes	No	
1	Self owned	Semi- perm	No	Yes	Yes	N/A	Yes	No	
1	Self owned	Semi- perm	No	Yes	No	N/A	Yes	No	
1	Self owned	Temporary	No	No	Yes	N/A	Yes	No	
1	Self owned	Semi- perm	Yes	No	No	N/A	Yes	No	
1	Self owned	Semi- perm	Yes	No	No	N/A	Yes	No	
1	Self owned	Semi- perm	No	No	Yes	N/A	Yes	No	
1	Self owned	Semi- perm	No	Yes	No	N/A	Yes	No	

charcoal	cooking_kerosene
No	No
No	No
No	No
No	No
No	No
No	No
No	No
No	No
No	No
No	No
No	No
No	No
No	No
No	No
No	No
No	No
No	No
No	No
No	No
No	No
No	No
No	No
No	No
No	No
No	No
No	No
No	No
No	No
No	No
No	No
No	No
No	No
No	No
No	No
No	No
No	No
No	No
No	No
No	No
No	No
No	No
No	No
No	No
No	No
No	No
No	No

No	No
No	No
No	No
No	No
No	No
No	No
No	No
No	No
No	No
Yes	No
No	No
No	No
No	No
No	No
No	No
Yes	No
No	No
No	No
No	No
No	No
No	No
No	No
No	No
Yes	No
No	No
No	No
No	No
No	No
No	No
No	No
No	No
No	No
No	No
No	No
No	No
No	No
No	No
No	No
No	No
No	No
No	No
No	No
No	No
No	No
No	No
No	No
No	No

No	No
Yes	No
No	No
No	No
No	No
No	No
No	No
No	No
No	No
No	No
No	No
No	No
No	No
No	No
No	No
No	No
No	No
No	No
No	No
No	No
No	No
No	No
No	No
No	No
No	No
No	No
No	No
Yes	No
No	No
No	No
No	No
No	No
No	No
No	No
No	No
No	No
No	No
No	Yes
No	No
No	No
No	No
No	Yes
No	No
No	No
No	No
No	No
No	No

No	No
No	No
No	No
No	No
No	No
No	No
No	No
No	No
No	No
No	No
No	No
No	No
No	No
No	No
No	No
No	No
No	No
No	No
No	No
No	No
No	No
No	No
No	No
No	No
No	No
No	No
No	No
No	No
No	No
No	No
No	No
No	No
No	No
No	No
No	No
Yes	No
No	No
No	No
No	No
No	No
No	No
No	No
Yes	No
No	No
No	No
No	No
Yes	No

No	No
No	No
No	No
No	No
No	No
No	No
No	No
No	No
No	No
No	No
No	No
No	No
No	No
No	No
Yes	No
No	No
No	No
No	No
No	No
No	No
No	No
No	No
Yes	No
No	No
No	No
No	No
Yes	No
Yes	No
Yes	No
No	No
Yes	No
Yes	No
Yes	No
No	No
No	No
No	No
No	No
No	No
No	No
No	No
No	No
Yes	No
No	No
No	No
No	No
No	No
No	No

No	No
Yes	No
No	No
No	No
No	No
No	No
No	No
No	No
Yes	No
No	No
No	No
No	No
Yes	No
No	No
No	No
No	No
Yes	No
No	No
No	No
No	No
No	No
No	No
No	No
No	No
No	No
No	No
No	No
Yes	No
No	No
No	No
No	No
No	Yes
No	Yes
No	No
No	No
No	No
No	No
No	No
No	No
No	No
No	No
Yes	No
Yes	No
Yes	No
No	No
No	No
No	No

No	No
No	No
No	No
Yes	No
Yes	No
Yes	No
No	No
Yes	No
Yes	No
No	No
No	No
No	No
No	No
No	No
No	No
No	No
No	No
No	No
No	No
No	No
No	No
No	No
No	No
No	No
Yes	No
Yes	No
Yes	No
No	No
No	No
No	No
No	No
No	No
No	No
